# Differences in socioeconomic position, lifestyle and health-related pregnancy characteristics between Pakistani and White British women in the Born in Bradford prospective cohort study: the influence of the woman's, her partner's and their parents’ place of birth

**DOI:** 10.1136/bmjopen-2014-004805

**Published:** 2014-06-19

**Authors:** J West, D A Lawlor, L Fairley, J Wright

**Affiliations:** 1 Bradford Institute for Health Research, Bradford Royal Infirmary, Bradford UK; 2 Leeds Institute of Health Sciences, University of Leeds, Leeds, UK; 3 MRC Integrative Epidemiology Unit at the University of Bristol, Bristol, UK; 4 School of Social and Community Medicine, University of Bristol, Bristol, UK

**Keywords:** Epidemiology, Ethnicity, Lifestyle

## Abstract

**Objective:**

To examine differences between Pakistani and White British women in relation to socioeconomic position, lifestyle and health-related pregnancy characteristics, and to determine whether these differences vary depending on the woman's, her partner's and both of their parents’ place of birth.

**Design:**

Prospective cohort study.

**Setting:**

Bradford, UK

**Participants:**

3656 Pakistani and 3503 White British women recruited to the Born in Bradford study.

**Main outcome measures:**

Socioeconomic position (employment status; level of education; receipt of benefits; housing tenure), lifestyle characteristics (body mass index (BMI) at the start of pregnancy; smoking during pregnancy) and health-related pregnancy characteristics (hypertensive disorders of pregnancy; gestational diabetes; fasting glucose, postload glucose and fasting insulin at ∼27 weeks gestation).

**Results:**

Fewer Pakistani women were employed (OR 0.17, 95% CI 0.15 to 0.19), the difference being markedly less for UK born women. UK born Pakistani women were more likely, and South Asian born less likely, to be educated post 16 than White British women. Smoking was uncommon among Pakistani women, though the difference comparing UK born Pakistani women to White British women was less than for other groups. BMI was lower among Pakistani compared to White British women (adjusted mean difference −1.12, 95% CI −1.43 to −0.81), the difference being greatest when partners were UK born irrespective of the woman’s place of birth. Pakistani women had higher fasting and postload glucose (mean difference 0.20 mmol/L, 95% CI 0.17 to 0.24; 0.37, 95% CI 0.28 to 0.45), higher fasting insulin and were more likely to have gestational diabetes (GDM).

**Conclusions:**

Our results suggest that some socioeconomic, lifestyle and pregnancy characteristics could be beginning to change in response to migration to the UK, with generally beneficial changes, that is, improving education and employment prospects, lower BMI and no evidence that being UK born has further increased the risk of GDM, but some negative, that is, slight increases in smoking.

**Strengths and limitations of this study**

- The strengths of this study include a large sample size and a range of outcomes including oral glucose tolerance test data and detailed ancestry information.
- We have, for the first time, been able to examine ethnic differences in socioeconomic, lifestyle and pregnancy characteristics using information on the place of birth of women and their partners. We had also set out to explore differences based on all four grandparents, but once we began analysing data it was apparent that for the majority of Pakistani women and their partners, all four of their parents were South Asian born. This limited our ability to explore differences across two generations, but highlights the persistence of strong family links in this community that has lived in Bradford for over 6 decades.
- A potential limitation is that our results may not be generalisable to other South Asian populations and further work will be important to track these differences over future generations of UK South Asian migrants.

## Introduction

Migration of South Asian populations to high-income countries is generally thought to offer socioeconomic advantages in the form of improved education and employment opportunities, better housing and access to healthcare. However, improvements in environmental circumstances do not necessarily translate into improvements in health outcomes. Indeed, South Asian migrant populations to the UK experience an increased risk of maternal^1^ and infant mortality^2^ and some chronic diseases^3^ compared with the UK population as a whole. This may reflect previous disadvantage associated with the country of origin which could persist over several generations, or could be a consequence of poor socioeconomic status within the host country. For example, UK South Asian communities are on average very poor.^4^ That is, it could be that in comparison to those who do not migrate, there are improved health outcomes, but these remain poorer in comparison to the indigenous population. A further explanation is that the adoption of unhealthy and sedentary lifestyles associated with acculturation or Westernisation, often characterised by low levels of physical activity,^5^ consumption of high calorie, energy-rich diets^6^ and cigarette smoking,^7^ ^8^ counteracts any potential health advantage of living in a higher income country. This may vary across different migrant communities, but where this is the case the adoption of such lifestyles may be particularly harmful to South Asian individuals who, for a given body mass index (BMI), have greater total and central adiposity and are known to be at greater risk of type 2 diabetes and cardiovascular disease than European adults.^8–11^

Ethnic differences in socioeconomic position and lifestyle that might impact on health during pregnancy could contribute to some of the known ethnic differences in pregnancy complications and perinatal outcomes. For example, they could contribute to the established greater risk of gestational diabetes (GDM)^12^ ^13^ and small for gestational age (SGA)^14–16^ in South Asian compared to White British women. They could also drive ethnic differences in future generations either through intrauterine effects of maternal behaviours on these or as a result of the adoption of parental lifestyles by offspring and a lack of social migration. Previous studies have reported ethnic differences in socioeconomic and lifestyle characteristics between South Asian and White British women during pregnancy. Findings from the Millennium Cohort Study suggest that South Asian women, in particular those originating from Pakistan and Bangladesh, are less likely to have formal educational qualifications, more likely to belong to lower socioeconomic groups and more likely to have never worked or be long-term unemployed.^7^ ^16^ Marked differences in smoking and alcohol consumption between South Asian and White British women have also been reported.^7^ ^17^ While outside pregnancy BMI is reportedly higher among South Asian women compared to White British women,^18^ we have previously reported that BMI is lower among Pakistani origin pregnant women in the Born in Bradford (BiB) cohort.^17^ Much less is known about maternal blood glucose and insulin, in particular whether there are differences in these outcomes across generations of UK South Asian migrants. To the best of our knowledge, no previous studies have examined ethnic differences in all these characteristics (socioeconomic, lifestyle, pregnancy) collectively, which is important to identify areas where South Asian women may have better outcomes and those where European women may have better outcomes. This knowledge could support the delivery of appropriate antenatal care aimed at maximising maternal and child health in both White British and South Asian groups.

Furthermore, previous studies have not explored whether any identified ethnic differences during pregnancy are consistent when the mother's, her partner's and both of their parents’ country of origin are taken into account. In a previous study, using data from the BiB cohort, which is used in this paper, we showed that birthweight was lower, but that birth fatness (assessed using skinfold thickness and cord blood leptin) was greater in Pakistani compared to White British infants.^17^ We further showed that these differences did not differ by whether both the mother and her partner and all four of their parents were born in the UK, all were born in South Asia or there was a mixed pattern between these two extremes.^17^ We extend that work to look at a range of socioeconomic position, lifestyle and pregnancy-related outcomes, in order to understand whether in the context of place of birth of women and her closest family relatives, there are some ethnic differences that are reduced or some that are enhanced, and if so whether these would be beneficial or detrimental to health.

The aim of this study was to examine differences between Pakistani women and White British women in relation to socioeconomic position (employment status; level of education; receipt of means tested benefits; housing tenure), lifestyle characteristics (BMI at the start of pregnancy; smoking during pregnancy) and health-related pregnancy characteristics (hypertensive disorders of pregnancy (HDP); GDM; fasting glucose, postload glucose and fasting insulin at ∼27 weeks gestation) and to determine whether these differences vary depending upon the woman's, her partner's and both of their parents’ place of birth.

## Methods

### Participants

The BiB study is a largely bi-ethnic prospective birth cohort study that recruited women during pregnancy and has followed them, their infants and their partners into the child's infancy. To be eligible for the study, women had to attend a booking clinic between March 2007 and December 2010 and be booked to give birth in the city of Bradford. Full details of the study methodology have been previously reported.^18^ Women were recruited at their oral glucose tolerance test (OGTT) appointment; all women booked for delivery in Bradford are offered a 75 g OGTT (comprising fasting and 2 h postload samples) at around 26–28 weeks gestation. Women who attended this appointment and agreed to take part in the study consented to the use of their obstetric medical records, had their height and weight recorded and completed an interviewer administered questionnaire. The questionnaire included questions relating to ethnicity, social and economic circumstances, smoking, alcohol, diet, education and employment and collected place of birth information for both parents and all four grandparents. Interviews were conducted in a range of South Asian languages (including Mirpuri, Bengali, Punjabi). Mirpuri is the most commonly spoken Asian language in Bradford but has no written script; therefore, questionnaires were transliterated, that is translated verbally to Mirpuri and then written phonetically, precisely as spoken to ensure that all interpreters translated it in the same way. Details of the language used to conduct the questionnaire were recorded. Ethics approval for the study was provided by the Bradford Local Research Ethics Committee (ref 06/Q1202/48). Data were available for 11 113 women recruited to the BiB cohort. We excluded stillbirths (n=64) and infants born to parents of ethnic origin other than White British or Pakistani (n=1598). Of the remaining 9451 participants, 7159 had complete data for all variables included in all models; thus, 3656 Pakistani and 3503 White British women are included in these analyses. Women with existing diabetes (0.5% of the BiB cohort) are not invited to attend for the glucose tolerance test as they are treated from the start of their pregnancy by an endocrine physician. As a result, these women were not recruited at the same time as other participants and do not have some data, including parental place of birth. These women are therefore not included in these complete case analyses.

### Woman's family member's place of birth

Ethnicity was self-reported at the interview, with participants given response options based on the UK Office of National Statistics guidance.^19^ Women completed a detailed ancestry interview, which included details of the place of birth of themselves, their partner and all four parents of themselves and their partner. Family place of birth groups of the Pakistani infants were derived from these data as previously reported.^17^ In the previous report, since our outcome of interest was infant birth size, the groups were defined in terms of ‘parents’ and ‘grandparents’. As our outcomes here are in pregnant women, we have described them in relation to her, but the groups are essentially the same as in the previous paper. Our aim in that previous paper, as here, was to examine differences across all possible groups based on place of birth of the woman, her partner and all four parents. Thus, we began by determining numbers in all 64 possible combinations of these six family members. Having done that, it was apparent that for almost all women, the four parents of the woman and her partner were South Asian born, meaning that the analyses were based primarily on the woman's and her partner's place of birth. Overall, 90% of women fell into one of four main categories:

1. Woman and her partner UK born and all four of their parents South Asian born;
2. Woman UK born, partner and all four of their parents South Asian born;
3. Partner UK born, woman and all four parents South Asian born;
4. Woman, her partner and four parents all South Asian born.

The remaining 11% (n=345), including those with one or more of the woman's or her partner's parents being UK born or where their parents’ place of birth was unknown, was combined to form one ‘other’ group.

### Outcome measures

#### Socioeconomic

Information on socioeconomic indicators (employment, education, receipt of benefits, housing tenure) was obtained from the interview with the woman at recruitment. We equivalised the mother's highest educational qualifications (based on the qualification received and the country obtained) into one of several categories using UK NARIC (http://www.ecctis.co.uk/naric/default.aspx): *<5 GCSE equivalent,*≥*5 GCSE equivalent, ‘A’ level equivalent, Higher than A-level equivalent, Other qualifications (eg, City and Guilds, RSA/OCR, BTEC), Don't know, Foreign Unknown.* Don't know relates to the mother responding ‘don't know’ during interview. Foreign Unknown relates to a qualification listed in the free text response but no level of qualification is given, or the qualification listed cannot be equivalised to one of the above categories. For these analyses, women were categorised as having been educated beyond the age of 16 or not (ie, *Higher than A-level equivalent, Other qualifications (eg, City and Guilds, RSA/OCR, BTEC).* Receipt of means tested benefits was based on the mother or her household receiving any of: income support, job seekers allowance, working tax credit or housing benefit. Housing tenure was categorised according to whether the woman lived in a household where the home was either part-owned (ie, mortgaged) or owned outright or not (ie, rented).

#### Lifestyle

BMI is used in these analyses as a proxy marker of lifestyle as it is an outcome that can potentially be influenced by changes or differences in lifestyle (in particular, dietary choices and levels of physical activity). At recruitment, women were weighed and their height measured (unshod and in light clothing) using SECA digital scales and a Leicester Height Measure, respectively. Weight at first antenatal clinic assessment when women were around 12 weeks gestation (median 12 weeks, IQR 11, 14) was abstracted from the antenatal records and this weight, together with height measured at recruitment, was used to calculate the woman's BMI so that this reflected early pregnancy BMI before substantial contribution from pregnancy and the growing fetus. Information on smoking was obtained at the questionnaire interview, with women categorised as having smoked cigarettes at any stage of their pregnancy or not. As none of the Pakistani origin women reported drinking alcohol, we were unable to include alcohol consumption as an outcome.

#### Health-related pregnancy characteristics

Women were classified as hypertensive in pregnancy if they had a systolic measure ≥140 and a diastolic ≥90 mm Hg on two or more occasions after 20 weeks gestation; information on this was obtained from the antenatal records. Fasting and postload glucose and fasting insulin were obtained from the OGTT plasma samples, which were assayed immediately after sampling at the biochemistry department of Bradford Royal Infirmary using the glucose oxidase method on Siemen's Advia 2400 chemistry autoanalysers. GDM was defined using the fasting and postload glucose according to WHO criteria^20^ at the time these women were pregnant as either a fasting glucose ≥6.1 mmol/L or a 2 h postload glucose ≥7.8 mmol/L. Women with existing diabetes prior to pregnancy did not complete an OGTT and are not included in this sample.

### Statistical analyses

All analyses were performed using Stata (V.12.1). We used univariable regression to examine the association of ethnicity and family place of the birth group with outcomes. Included predictor variables were decided a priori based on existing evidence and knowledge. Logistic regression was used for binary outcomes and linear regression for continuous outcomes, with the White British group used as the reference for all analyses, that is, we compared outcomes in ‘all’ Pakistani women and then each of the five family place of birth subgroups of Pakistani women to outcomes in White British women. The rationale for this is because our aim is primarily to compare all Pakistani origin women with White British women and then to compare subgroups based on place of birth with the same reference group of White British women to see if place of birth of the Pakistani women influences the extent to which they differ or not from the indigenous population. In all adjusted analyses, we adjusted for maternal age and parity (model 1). For the lifestyle outcomes (early pregnancy BMI; smoking), we also adjusted for each of the indicators of socioeconomic position in order to explore the extent to which any differences in these lifestyles might reflect ethnic differences in socioeconomic position (model 2). For the health-related pregnancy characteristics, we also adjusted for socioeconomic indicators (model 2) and also for the lifestyle characteristics (BMI; smoking) (model 3), to explore whether these explained any of the differences. When age and BMI were included in models as covariables, they were used as continuous variables. The existing literature supports their linear associations with outcomes and we confirmed this graphically. For all multivariable models, we examined the residuals and these were all found to be approximately normal. Further, we checked potential problems with collinearity in each model by assessing variance inflation and found that this was lower than 2 for all independent variables in all models.

## Results

The characteristics of White British and Pakistani origin women are shown in table 1. There was little difference between the two ethnic groups in mean gestation, premature births and infant sex. As reported in our previous paper,^17^ birth weight of their infant was markedly lower in Pakistani compared to White British women when all Pakistani origin women were combined and also when compared by subgroups based on place of birth. On average, Pakistani origin women were slightly older, in particular when both parents were South Asian born, markedly more likely to be married and have lived within larger households than White British women. These differences were similar across all generation groups. Pakistani women were shorter than White British women, but the difference was less when they were UK born. There were also some differences in parity across Pakistani generation groups; for example, parity was on average lowest when both parents were UK born and highest when both parents were born in South Asia.

**Table 1:** Characteristics of women and infants (N=9450) by ethnic and generation group

	White British (UK and Ireland)	All Pakistani births	Pakistani subgroups defined by place of birth of parents				
			Pakistani: Woman and partner UK born*	Pakistani: Woman UK born, partner SA born*	Pakistani: Partner UK born, woman SA born*	Pakistani: Woman and partner SA born*	Pakistani: other
Number	3503	3656	383	992	876	1060	345
Gestation at delivery (weeks) Mean (SD)	39.0 (1.9)	39.0 (1.8)	39.0 (1.8)	38.9 (1.9)	39.0 (1.9)	39.1 (1.7)	39.1 (1.6)
Births before 37 weeks N (%)	209 (6.0)	204 (5.6)	22 (5.7)	63 (6.4)	50 (5.7)	52 (4.9)	17 (4.9)
Mean birth weight in g (SD)	3346 (568)	3124 (540)	3114 (538)	3100 (549)	3101 (537)	3160 (547)	3158 (497)
Sex N (%)							
** **Male	1808 (52)	1851 (51)	200 (52)	504 (51)	420 (48)	535 (51)	192 (56)
** **Female	1695 (48)	1805 (49)	183 (48)	488 (49)	456 (52)	525 (49)	153 (44)
Maternal age Mean (SD)	27 (6)	28 (5)	28 (5)	28 (5)	27 (5)	30 (5)	26 (5)
Maternal height (m)	1.64	1.60	1.61	1.60	1.59	1.59	1.61
Mean (SD)	(0.06)	(0.06)	(0.05)	(0.06)	(0.05)	(0.05)	(0.06)
Parity N (%)							
0	1688 (48)	1157 (32)	155 (40)	331 (33)	253 (29)	254 (24)	164 (47)
1	1122 (32)	986 (26)	105 (27)	261 (26)	253 (29)	265 (25)	102 (30)
2	454 (13)	754 (21)	76 (20)	194 (20)	199 (23)	233 (22)	52 (15)
3	139 (4)	462 (13	34 (9)	125 (13)	111 (12)	178 (17)	14 (4)
4 or more	100 (3)	297 (8)	13 (4)	81 (8)	60 (7)	130 (12)	13 (4)
Married N (%)	1149 (33)	3571 (98)	364 (95)	974 (98)	862 (98)	1051 (99)	320 (93)
Living with a partner N (%)	2518 (72)	4702 (93)	352 (92)	898 (91)	852 (97)	1001 (95)	303 (88)
Consumed alcohol during pregnancy N (%)	266 (8)	0 (0)	0 (0)	0 (0)	0 (0)	0 (0)	0 (0)
Total number of household members Mean (SD)	3 (1)	5 (3)	5 (3)	5 (2)	6 (3)	5 (2)	5 (3)

*All four parents of the woman and her partner South Asian (SA) born.

The odds of being in employment for Pakistani women were 83% lower than for White British women (adjusted OR 0.17, 95% CI 0.15 to 0.19%), but there were differences by family place of birth (table 2). These odds were 94% less for those who were South Asian born, but this difference reduced to 60% for Pakistani women when both they and their partner were UK born. Following adjustment for maternal age and parity, Pakistani women as a whole were more likely to be educated beyond the age of 16 than White British women (OR 1.15, 95% CI 1.04 to 1.27%); however, there were marked differences across family place of birth groups with women who were South Asian born being less likely, and those who were UK born being more likely compared to White British women, to be educated beyond 16 years. Being in receipt of means tested benefits was similar in both ethnic groups when Pakistani women were assessed as a whole (adjusted OR 0.97, 95% CI 0.87 to 1.09%), although for Pakistani women who were UK born with a South Asian partner there were increased odds of receiving benefits. Compared to White British women, Pakistani women were considerably more likely to own or part-own their home (adjusted OR 2.30, 95% CI 2.07 to 2.56%), and this was consistent across all family place of birth groups.

**Table 2:** Unadjusted and adjusted* ORs (95% CI) for socioeconomic characteristics for ethnic and generation groups

	White British N=3503	All Pakistani births N=3656	Pakistani subgroups defined by place of birth of parents				
			Pakistani: Woman and partner UK born† N=383	Pakistani: Woman UK born, partner SA born† N=992	Pakistani: Partner UK born, woman SA born† N=876	Pakistani: Woman and partner SA born† N=1060	Pakistani: other N=345
In employment							
Number (%)	2272 (65)	881 (24)	175 (46)	388 (39)	81 (9)	132 (12)	105 (30)
Unadjusted OR	1	0.17 (0.16 to 0.19)	0.46 (0.37 to 0.56)	0.35 (0.30 to 0.40)	0.06 0.04 to 0.07)	0.08 (0.06 to 0.09)	0.24 (0.19 to 0.30)
Adjusted OR*	1	0.17 (0.15 to 0.19)	0.40 (0.32 to 0.51)	0.38 (0.32 to 0.44)	0.06 (0.04 to 0.07)	0.06 (0.05 to 0.08)	0.25 (0.19 to 0.32)
Educated post 16							
Number (%)	1601 (46)	1578 (43)	235 (1)	473 (48)	306 (35)	401 (38)	163 (47)
Unadjusted OR	1	0.90 (0.82 to 0.99)	1.89 (1.52 to 2.34)	1.08 (0.94 to 1.25)	0.64 (0.55 to 0.74)	0.72 (0.63 to 0.83)	1.06 (0.85 to 1.33)
Adjusted OR*	1	1.15 (1.04 to 1.27)	2.14 (1.70 to 2.68)	1.37 (1.18 to 1.59)	0.86 (0.73 to 1.02)	0.88 (0.75 to 1.03)	1.39 (1.11 to 1.76)
In receipt of means tested benefits‡							
Number (%)	1334 (38)	1742 (48)	163 (43)	534 (54)	387 (44)	523 (49)	135 (39)
Unadjusted OR	1	1.48 (1.35 to 1.63)	1.20 (0.97 to 1.49)	1.90 (1.64 to 2.19)	1.29 (1.11 to 1.49)	1.58 (1.38 to 1.89)	1.05 (0.83 to 1.31)
Adjusted OR*	1	0.97 (0.87 to 1.09)	1.02 (0.79 to 1.30)	1.42 (1.20 to 1.67)	0.71 (0.60 to 0.84)	0.91 (0.78 to 1.08)	0.84 (0.65 to 1.09)
Housing tenure: owns/part-owns (mortgage)							
Number (%)	1875 (54)	2600 (71)	283 (74)	730 (74)	669 (76)	698 (66)	220 (64)
Unadjusted OR	1	2.14 (1.94 to 2.36)	2.46 (1.94 to 3.12)	2.42 (2.07 to 2.83)	2.81 (2.37 to 3.32)	1.67 (1.45 to 1.93)	1.53 (1.21 to 1.92)
Adjusted OR*	1	2.30 (2.07 to 2.56)	2.49 (1.95 to 3.18)	2.60 (2.20 to 3.06)	3.35 (2.80 to 3.99)	1.55 (1.32 to 1.80)	2.02 (1.60 to 2.57)

*Adjusted for maternal age; parity.

†All four parents of the woman and her partner South Asian (SA) born.

‡Any of: income support; job seekers allowance; working tax credit; housing benefits.

**Figure 1:**
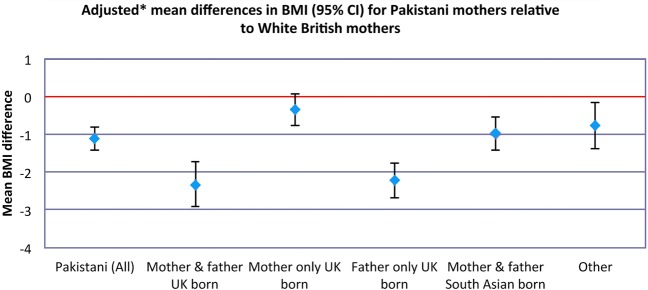
Adjusted mean differences in body mass index for Pakistani women relative to White British women. *Model 2: Adjusted for maternal age; parity; employment; post-16 education; receipt of means tested benefits; housing tenure.

Table 3 shows the unadjusted and adjusted (models 1 and 2) ethnic difference in lifestyle characteristics. Pakistani women had a lower BMI than White British women (adjusted (model 2) mean difference −1.12 95% CI −1.43 to −0.81%), but the difference was much greater when the woman's partner was UK born, irrespective of where the woman herself was born (figure 1). The odds of smoking for Pakistani women were around 94% less and this was similar across generation groups other than when both the woman and her partner were UK born, in which case they were 85% less. None of the Pakistani women reported drinking any alcohol during pregnancy (0%), whereas 8% of White British women drank during pregnancy.

**Table 3:** Unadjusted and adjusted* mean difference/ORs (95% CI) for lifestyle characteristics for ethnic and generation groups

	White British N=3503	All Pakistani births N=3656	Pakistani subgroups defined by place of birth of parents				
			Pakistani: Woman and partner UK born† N=383	Pakistani: Woman UK born, partner SA born† N=992	Pakistani: Partner UK born, woman SA born† N=876	Pakistani: Woman and partner SA born† N=1060	Pakistani: other N=345
BMI at start of pregnancy							
Mean (SD)	**26.8** (**5.9)**	**25.7** (**5.4)**	24.3 (4.6)	26.7 (5.7)	24.4 (4.7)	26.4 (5.6)	25.4 (5.3)
Unadjusted mean difference	**0**	**−1.15** (**−1.41** to **−0.88)**	−2.53 (−3.13 to −1.94)	−0.15 (−0.55 to 0.25)	−2.44 (−2.86 to −2.02)	−0.43 (−0.82 to −0.04)	−1.40 (−2.02 to −0.77)
Adjusted mean difference: model 1*	**0**	**−1.75** (**−2.01** to **−1.49)**	−2.84 (−3.41 to −2.26)	−0.73 (−1.12 to −0.34)	−2.95 (−3.36 to −2.54)	−1.49 (−1.88 to −1.10)	−1.22 (−1.83 to −0.62)
Adjusted mean difference: model 2‡	**0**	**−1.12** (**−1.43** to **−0.81)**	−2.32 (−2.92 to −1.72)	−0.35 (−0.76 to 0.07)	−2.22 (−2.69 to −1.75)	−0.99 (−1.43 to −0.57)	−0.77 (−1.39 to −0.15)
Smoked during pregnancy							
Number (%)	**1183** (**34)**	123 (3)	25 (7)	47 (5)	7 (0.8)	18 (2)	26 (8)
Unadjusted OR	**1**	**0.07** (**0.06** to **0.08)**	0.14 (0.09 to 0.21)	0.09 (0.07 to 0.13)	0.02 (0.01 to 0.03)	0.03 (0.02 to 0.05)	0.16 (0.11 to 0.24)
Adjusted OR: model 1*	**1**	**0.06** (**0.05** to **0.07)**	0.13 (0.09 to 0.20)	0.09 (0.06 to 0.12)	0.01 (0.01 to 0.03)	0.03 (0.02 to 0.05)	0.12 (0.08 to 0.19)
Adjusted OR: model 2‡	**1**	**0.06** (**0.05** to **0.08)**	0.15 (0.09 to 0.23)	0.09 (0.07 to 0.13)	0.01 (0.01 to 0.03)	0.03 (0.02 to 0.05)	0.13 (0.08 to 0.19)

*Adjusted for maternal age; parity.

†All four parents of the woman and her partner South Asian (SA) born.

‡Adjusted for maternal age; parity; employment status; level of education, receipt of means tested benefits; housing tenure.

**Figure 2:**
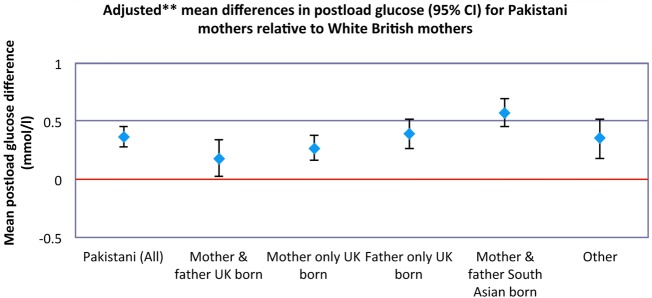
Adjusted mean differences in postload glucose for Pakistani women relative to White British women. **Model 3: Adjusted for maternal age; parity; employment; post-16 education; receipt of means tested benefits; housing tenure; early pregnancy body mass index; smoking in pregnancy.

In table 4, the unadjusted and adjusted (models 1–3) ethnic difference in pregnancy characteristics is shown. Fewer Pakistani women in general had HDP (adjusted (model 3) OR 0.87, 95% CI 0.67 to 1.13%), although this result was imprecisely estimated with wide CIs that included the null. This was not consistent across all family place of birth groups; for example, women who were South Asian born were slightly more likely to have HDP than White British women, and this was the case in all three adjusted models. Pakistani women were more likely to have GDM and higher fasting and postload glucose and fasting insulin than White British women, and these differences were broadly similar across all three models of adjustment. There were some differences by family place of birth group; for example, the difference in postload glucose between Pakistani and White British women was far greater when the woman and her partner were born in South Asia than when both were UK born (adjusted mean difference (model 3) 0.57, 95% CI 0.45 to 0.69% and 0.18, 95% CI 0.02 to 0.34%, respectively, and figure 2).

**Table 4:** Unadjusted and adjusted* mean difference/ORs (95% CI) for health-related pregnancy characteristics for ethnic and generation groups

	White British N=3503	All Pakistani births N=3656	Pakistani subgroups defined by place of birth of parents				
			Pakistani: Woman and partner UK born† N=383	Pakistani: Woman UK born, partner SA born† N=992	Pakistani: Partner UK born, woman SA born† N=876	Pakistani: Woman and partner SA born† N=1060	Pakistani: other N=345
Hypertensive disorders of pregnancy							
Number (%)	**239** (**7)**	**188** (**5)**	16 (4)	51 (5)	43 (5)	66 (6)	12 (3)
Unadjusted OR	**1**	**0.74** (**0.61** to **0.90)**	0.59 (0.35 to 0.99)	0.74 (0.54 to 1.01)	0.70 (0.51 to 0.98)	0.91 (0.68 to 1.20)	0.49 (0.27 to 0.89)
Adjusted OR: model 1*	**1**	**0.82** (**0.67** to **1.01)**	0.62 (0.37 to 1.04)	0.82 (0.59 to 1.12)	0.85 (0.61 to 1.19)	0.99 (0.74 to 1.33)	0.56 (0.31 to 1.01)
Adjusted OR: model 2‡	**1**	**0.82** (**0.64** to **1.04)**	0.62 (0.36 to 1.06)	0.81 (0.58 to 1.13)	0.87 (0.59 to 1.29)	1.01 (0.73 to 1.40)	0.56 (0.31 to 1.03)
Adjusted OR: model 3§	**1**	**0.87** (**0.67** to **1.13)**	0.78 (0.45 to 1.35)	0.80 (0.56 to 1.14)	1.06 (0.70 to 1.61)	1.06 (0.75 to 1.49)	0.57 (0.31 to 1.06)
Gestational diabetes							
Number (%)	**172** (**5)**	406 (11)	30 (8)	96 (10)	92 (11)	159 (15)	29 (8)
Unadjusted OR	**1**	2.42 (2.01 to 2.91)	1.65 (1.09 to 2.46)	2.07 (1.59 to 2.69)	2.27 (1.74 to 2.96)	3.42 (2.72 to 4.29)	1.78 (1.18 to 2.68)
Adjusted OR: model 1*	**1**	2.41 (1.98 to 2.94)	1.66 (1.10 to 2.49)	2.07 (1.58 to 2.71)	2.54 (1.92 to 3.35)	3.01 (2.36 to 3.83)	2.24 (1.47 to 3.41)
Adjusted OR: model 2‡	**1**	2.28 (1.82 to 2.86)	1.66 (1.09 to 2.53)	1.98 (1.49 to 2.64)	2.47 (1.79 to 3.39)	2.89 (2.20 to 3.82)	2.21 (1.44 to 3.40)
Adjusted OR: model 3§	**1**	2.38 (1.86 to 3.03)	1.89 (1.23 to 2.92)	1.98 (1.46 to 2.67)	2.82 (2.01 to 3.97)	3.04 (2.27 to 4.08)	2.29 (1.47 to 3.56)
Fasting glucose Mean (SD)	**4.41** (**0.41)**	**4.62** (**0.62)**	4.54 (0.47)	4.58 (0.64)	4.54 (0.48)	4.73 (0.76)	4.60 (0.53)
Unadjusted mean difference	**0**	**0.20** (**0.18** to **0.23)**	0.13 (0.08 to 0.19)	0.17 (0.14 to 0.21)	0.13 (0.09 to 0.17)	0.32 (0.29 to 0.36)	0.19 (0.13 to 0.25)
Adjusted mean difference: model 1*	**0**	**0.18** (**0.16** to **0.21)**	0.12 (0.06 to 0.17)	0.15 (0.11 to 0.19)	0.12 (0.09 to 0.16)	0.27 (0.24 to 0.31)	0.22 (0.16 to 0.27)
Adjusted mean difference: model 2‡	**0**	**0.18** (**0.15** to **0.21)**	0.12 (0.06 to 0.18)	0.15 (0.11 to 0.19)	0.12 (0.07 to 0.16)	0.27 (0.23 to 0.31)	0.22 (0.16 to 0.27)
Adjusted mean difference: model 3§	**0**	**0.20** (**0.17** to **0.24)**	0.17 (0.12 to 0.23)	0.16 (0.12 to 0.19)	0.17 (0.12 to 0.21)	0.29 (0.25 to 0.33)	0.23 (0.17 to 0.29)
Postload glucose							
Mean (SD)	**5.47** (**1.30)**	**5.89** (**1.68)**	5.59 (1.35)	5.81 (1.58)	5.82 (1.50)	6.12 (2.02)	5.73 (1.45)
Unadjusted mean difference	**0**	**0.42** (**0.35** to **0.49)**	0.12 (−0.04 to 0.28)	0.34 (0.23 to 0.45)	0.35 (0.24 to 0.46)	0.72 (0.62 to 0.83)	0.26 (0.09 to 0.42)
Adjusted mean difference: model 1*	**0**	**0.37** (**0.29** to **0.44)**	0.08 (−0.07 to 0.24)	0.29 (0.18 to 0.39)	0.35 (0.24 to 0.46)	0.58 (0.48 to 0.69)	0.35 (0.19 to 0.51)
Adjusted mean difference: model 2‡	**0**	**0.35** (**0.27** to **0.43)**	0.10 (−0.06 to 0.26)	0.28 (0.17 to 0.39)	0.33 (0.20 to 0.46)	0.56 (0.44 to 0.68)	0.34 (0.18 to 0.51)
Adjusted mean difference: model 3§	**0**	**0.37** (**0.28** to **0.45)**	0.18 (0.02 to 0.34)	0.27 (0.16 to 0.38)	0.39 (0.26 to 0.52)	0.57 (0.45 to 0.69)	0.35 (0.18 to 0.52)
Fasting insulin Mean (SD)	**81.40** (**46.72)**	**100.28** (**62.76)**	92.66 (65.59)	100.76 (56.46)	91.75 (49.04)	106.11 (68.84)	111.09 (81.89)
Unadjusted mean difference	**0**	**18.88** (**16.31** to **21.45)**	11.26 (5.42 to 17.09)	19.36 (15.46 to 23.26)	10.36 (6.26 to 14.45)	24.71 (20.91 to 28.51)	29.69 (23.58 to 35.81)
Adjusted mean difference: model 1*	**0**	**18.08** (**15.42** to **20.74)**	10.98 (5.13 to 16.82)	18.59 (14.64 to 22.54)	9.67 (5.51 to 13.83)	23.36 (19.43 to 27.30)	29.69 (23.55 to 35.82)
Adjusted mean difference: model 2‡	**0**	**21.29** (**18.13** to **24.45)**	14.01 (7.95 to 20.08)	20.62 (16.40 to 24.83)	13.53 (8.73 to 18.34)	25.24 (20.89 to 29.59)	32.01 (25.72 to 38.31)
Adjusted mean difference: model 3§	**0**	**25.71** (**22.73** to **28.69)**	24.44 (19.03 to 29.86)	21.29 (17.47 to 25.13)	23.27 (18.86 to 27.68)	29.03 (25.04 to 33.02)	34.79 (29.18 to 40.39)

*Adjusted for maternal age; parity.

†All four parents of the woman and her partner South Asian (SA) born.

‡Adjusted for maternal age; parity; employment status; level of education, receipt of means tested benefits; housing tenure.

§Adjusted for maternal age; parity; employment status; level of education, receipt of means tested benefits; housing tenure; early pregnancy body mass index; smoking in pregnancy.

## Discussion

We have shown differences across a range of socioeconomic, lifestyle and pregnancy characteristics between Pakistani and White British origin women, and these vary depending on whether Pakistani women are born in the UK or South Asia. We have, for the first time, been able to consider not only the woman's place of birth, but also her partner’s and both of their parents’ place of birth, though after preliminary analyses it was clear that for the majority of women and their partners, all four of their parents were South Asian born. This provides important information about how these differences might be reduced or even enhanced with greater acculturation over generations. For example, the odds of Pakistani women as a whole being in employment were 83% less than in White British women, but across generation groups this difference varied from 60%, when both the woman and her partner were born in the UK, to 94% when both the woman and her partner were South Asian born. Likewise, we found interesting differences in education attainment between Pakistani and White British women. Overall, Pakistani women were slightly more likely to have been educated beyond the age of 16, but this was driven by UK born Pakistani women, especially those with a UK born partner, who were twice as likely as White British women to have completed education beyond age 16. In contrast, South Asian born Pakistani women, irrespective of their partner’s place of birth, were less likely than White British women to have been educated beyond the age of 16. This could reflect a positive effect of migration and acculturation on social mobility, which most likely plays a part in the employment differences described above and is consistent with previous reports.^7^ ^21^ While differences in employment and education by place of birth suggest the adoption of some British lifestyle characteristics, the tendency of Pakistani women to live within larger households and to be more likely to own or part-own their own home suggests that the traditional culture of living within extended families has been maintained across all place of birth subgroups of Pakistani women. Living with an extended family could have considerable benefits for the mother and her offspring, such as childcare support and greater social capital, but could also result in overcrowding and potential detrimental impacts of this on health.^22^ Early analyses using data from BiB suggests that living with more family members does not lead to greater family social capital (Cabieses B, unpublished data 2013). Pakistani women who were born in the UK but had a South Asian born partner were more likely to claim benefits, compared to White British women, than those who were South Asian born, which is surprising given that they tend to be more likely to be in employment. This might reflect a tendency for South Asian born partners to be in lower paid employment reducing total household income, or that poorer command of the English language (more likely among those Pakistani women who were South Asian born and were less likely to claim benefits compared to White British women) is a barrier to accessing services and social support.

Greater social migration, for example, coming to the UK for social reasons, has been associated with increased uptake of lifestyle characteristics of the host country such as smoking and alcohol consumption.^7^ We report a similar trend in that UK born Pakistani origin women were more likely to smoke than South Asian born women, but smoking was still uncommon among all Pakistani women compared to White British women and none of them reported any alcohol consumption during pregnancy. Thus, the increase in these harmful health behaviours over generations in some migrant groups, while showing some signs of change, appears to be minimal among Pakistani women. This may reflect persisting cultural or religious influences^23^ ^24^ and could be related to the fact that for the majority of women, both of their parents and their partners’ parents were South Asian born, We found BMI to be slightly lower among Pakistani origin women compared to White British women, although there were interesting differences across family place of birth groups. The finding that the difference in BMI between Pakistani and White British women was markedly greater for Pakistani women with a UK born partner, irrespective of their own place of birth, than for women with a South Asian born partner is particularly striking. One possible explanation is that within this population, partners/husbands have a particularly dominant role.^25^ Thus, the lifestyle choices of the family or household will be driven mostly by the social norms and habits of the partner. In the case of men born in the UK, these are likely to be influenced by western culture, which promotes a lower BMI as both healthy and attractive. Similarly, having been brought up and educated in the UK, they may be more likely to participate in organised physical activity and also may be more receptive to UK public health campaigns.

Health-related pregnancy characteristics may be most important to the long-term health of South Asian migrants in the UK, particularly in relation to the association of these characteristics with cardiovascular disease and type 2 diabetes.^26^ We report a number of differences between Pakistani women and White British women in HDP, glucose tolerance, fasting insulin and GDM. Pakistani women as a whole group were less likely to have HDP, although this was not consistent across family subgroups, but they were more than twice as likely to have GDM. Consistent with these higher rates of GDM, Pakistani women had higher fasting and postload glucose and higher fasting insulin than White British women. These findings are similar to those from previous studies showing that South Asian women are more likely to have GDM than White European women.^12^ ^13^ They are also consistent with considerable evidence that adult non-pregnant women and men have a higher risk of insulin resistance and type 2 diabetes.^9–11^ ^26^ We found that the increased likelihood of Pakistani women having GDM compared to White British women was greatest for South Asian born women. We also found that the mean difference in fasting and postload glucose and fasting insulin relative to White British women was substantively greater when the woman and her partner were both born in South Asia. This is somewhat surprising as evidence suggests that the increased risk of insulin resistance and type 2 diabetes in South Asian adults compared to White Europeans is largely among those in urban (rather than rural) areas of South Asia^27^ or in those who have migrated to Western countries.^9^ ^28^ We might therefore have expected the increase to be greater among those who were UK born. The difference between our findings and these previous studies of non-pregnant migrants^9^ ^26^ ^27^ might be explained by differences in the population studied, with many of these previous studies being of Indian, or mixed rather than Pakistani origin. Pakistani migrants in general tend to be poorer, shorter and weigh less, and the Pakistani women in this study have lower BMI than the White British women. For religious and cultural reasons, Pakistani women remain unlikely to smoke or drink alcohol, which could influence their glucose tolerance, although smoking is related to lower BMI and therefore would be expected to reduce glucose tolerance.^29^ It might also be that while insulin resistance and diabetes in the general population are enhanced in those who migrate and particularly with a greater duration of migration, in pregnancy the impact of place of birth or time since migration differs. We are not aware of other studies with equivalent data to explore this further, but it would be interesting to see if this finding is replicated.

The key strengths of this study are the large sample size, range of outcomes we have been able to examine, including OGTT data, and the detailed information on place of birth. To the best of our knowledge, this is the first study to examine differences between Pakistani and White British women in relation to socioeconomic, lifestyle and pregnancy characteristics using detailed information on the place of birth of women and their partners. We had hoped to explore three generations of Pakistani migrants to Bradford, but for almost all the women in this study, their parents and the parents of their partner were born in South Asia. However, this is in itself an interesting finding and useful for meeting future health needs in the city. It might also explain some of our findings in relation to the persistence of some characteristics across family place of birth subgroups. A potential limitation of our study was the inability to include other South Asian groups in our analyses (Indian and Bangladeshi) due to the small numbers within our cohort. On the one hand, examining a specific South Asian population (Pakistani) reduces the problem of heterogeneity between South Asian groups, but at the same time it may limit the generalisability of our results to other South Asian populations. Our analyses have not accounted for South Asians who migrate to the UK in childhood and may be resident in the UK for much of their development and education, which could potentially dilute any differences between the Pakistani place of birth groups. Within BiB information, regarding the age at which an individual migrated to the UK is only available for women (not their partner or parents); therefore, we were not able to account for this in our family place of birth groups. We were not able to validate the self-report of smoking or alcohol consumption in pregnancy for either the Pakistani or White British women. If reporting bias, which might occur because of the stigma associated with these behaviours in pregnancy, is similar in each ethnic group, it should not bias the comparisons that are the main focus of this paper. Many of the researchers who collected the interview data were of Pakistani origin, and it is possible that this may have resulted in greater under-reporting in Pakistani origin women. However, the prevalence of these behaviours in this study is similar to those in other studies of Pakistani women.^7^

In summary, we have found some evidence that the difference in some of these characteristics between Pakistani and White British women may be changing in response to migration to the UK, in that differences were seen most often in those where the woman or her partner were UK born. Several of these differences would be beneficial to health and well-being. For example, Pakistani women born in the UK were more likely than White British women to be educated beyond age 16. UK born Pakistani women were also more similar to White British women in terms of employment and there was no evidence that being UK born increased their risk of GDM or glucose intolerance. On the other hand, while the overall prevalence of smoking in Pakistani women in all groups was very small, the difference between them and White British women was least when they were UK born. We have also identified differences that vary according to the woman's partner's place of birth; for example, BMI is lower among Pakistani women with a UK born partner. Further work is needed that continues to track these important ethnic differences over future generations to support the delivery of appropriate antenatal care.

## Supplementary Material

Author's manuscript

## References

[1] Cantwell R Clutton-Brock T Cooper G Saving Mothers’ Lives: Reviewing maternal deaths to make motherhood safer: 2006–2008. BJOG 2011;118 (Suppl 1):1–203 21356004 10.1111/j.1471-0528.2010.02847.x

[2] Office for National Statistics (ONS). Gestation-specific infant mortality in England and Wales, 2010. London: ONS, 2012 http://www.ons.gov.uk/ons/dcp171778_282579.pdf (accessed on 18 Mar 2014).

[3] Bhala N Zaman MS Preventing premature mortality in chronic diseases for South Asians in the UK and beyond. J R Soc Med 2009;102:459–63 19875534 10.1258/jrsm.2009.090112 PMC2770360

[4] Nazroo J Ethnicity, class and health. London: Policy Studies Institute, 2001

[5] Williams ED Stamatakis E Chandola T Assessment of physical activity levels in South Asians in the UK: findings from the Health Survey for England. J Epidemiol Community Health 2011;65:517–21 20525752 10.1136/jech.2009.102509

[6] Patel JV Vyas A Cruickshank JK Impact of migration on coronary heart disease risk factors: comparison of Gujaratis in Britain and their contemporaries in villages of origin in India. Atherosclerosis 2006;185:297–306 16005463 10.1016/j.atherosclerosis.2005.06.005

[7] Hawkins SS Lamb K Cole TJ Influence of moving to the UK on maternal health behaviours: prospective cohort study. BMJ 2008;336:1052 18403500 10.1136/bmj.39532.688877.25 PMC2375984

[8] Sproston K Mindell JE Health Survey for England 2004. The health of minority ethnic groups. HSE 2006. London: Information Centre, 2006.

[9] Barnett AH Dixon AN Bellary S Type 2 diabetes and cardiovascular risk in the UK south Asian community. Diabetologia 2006;49:2234–46 16847701 10.1007/s00125-006-0325-1

[10] Mather HM Keen H The Southall Diabetes survey: prevalence of known diabetes in Asians and Europeans. BMJ 1985;291:1081–4 3931804 10.1136/bmj.291.6502.1081 PMC1417018

[11] Gholap N Davies M Patel K Type 2 diabetes and cardiovascular disease in South Asians. Prim Care Diabetes 2011;5:45–56 20869934 10.1016/j.pcd.2010.08.002

[12] Dornhurst A Pateson CM Nicholls JS High prevalence of gestational diabetes mellitus in women from ethnic minority groups. Diabet Med 1992;9:820–5 1473322 10.1111/j.1464-5491.1992.tb01900.x

[13] Makgoba M Savvidou MD Steer PJ An analysis of the interrelationship between maternal age, body mass index and racial origin in the development of gestational diabetes mellitus. BJOG 2012;119:276–82 22044452 10.1111/j.1471-0528.2011.03156.x

[14] Margetts BM Mohd Yusof S Al Dallal Z Persistence of lower birth weight in second generation South Asian babies born in the United Kingdom. J Epidemiol Community Health 2002;56:684–7 12177085 10.1136/jech.56.9.684 PMC1732245

[15] Leon DA Moser K Low birth weight persists in South Asian babies born in England & Wales regardless of maternal country of birth. Slow pace of acculteration, physiological constraint or both? Analysis of routine data. J Epidemiol Community Health 2012;66:544–51. 21118952 10.1136/jech.2010.112516

[16] Kelly Y Panico L Bartlet M Why does birthweight vary among ethnic groups in the UK? Findings from the Millennium Cohort Study. J Public Health (Oxf) 2008;31:131–7 18647751 10.1093/pubmed/fdn057

[17] West J Lawlor DA Fairley L UK born Pakistani origin infants are relatively more adipose than White British infants: findings from 8704 mother-offspring pairs in the Born in Bradford prospective birth cohort. J Epidemiol Community Health 2013;67:544–51. 23592862 10.1136/jech-2012-201891 PMC3859677

[18] Wright J Small N Raynor P Cohort profile: the Born in Bradford multi-ethnic family cohort study. Int J Epidemiol 2013;42:978–91 23064411 10.1093/ije/dys112

[19] Office for National Statistics (ONS). Ethnic group statistics: a guide for the collection and classification of ethnicity data. London: Stationary Office, 2003

[20] World Health Organisation. Definition, diagnosis and classification of diabetes mellitus and its complications. Report of a WHO consultation. Part 1: diagnosis and classification of diabetes mellitus. WHO, 1999 http://whqlibdoc.who.int/hq/1999/who_ncd_ncs_99.2.pdf (accessed on 18 Mar 2014).

[21] Berthaud R Family formulation in multicultural Britain: diversity and change. In: Loury GC Modood T Teles SM eds Ethnicity, social mobility and public policy. Cambridge: Cambridge University Press, 2005:222–54

[22] Galobardes B Shaw M Lawlor DA Indicators of socioeconomic position (Part 1). J Epidemiol Community Health 2006;60:7–12 16361448 10.1136/jech.2004.023531 PMC2465546

[23] Bradby H Williams R Is religion or culture the key feature in changes in substance use after leaving school? Young Punjabis and a comparison group in Glasgow. Ethn and Health 2006;11:307–24 10.1080/13557850600628372 16774880

[24] Hurcombe R Bayley M Goodman A Ethnicity and alcohol. A review of the UK literature. Joseph Rowntree Foundation, 2010

[25] Beishon S Modood T Virdee S Ethnic minority families. London: Policy Studies Institute, 1998

[26] McKeigue PM Pierpoint T Ferrie JE Relationship of glucose intolerance and hyperinsulinaemia to body fat pattern in South Asians and Europeans. Diabetologia 1992;35:785–91 1511807 10.1007/BF00429101

[27] Ebrahim S Kinra S Bowen L The effect of rural-to-urban migration on obesity and diabetes in India: a cross-sectional study. PLoS Med 2010;7:e1000268 20436961 10.1371/journal.pmed.1000268 PMC2860494

[28] Landman J Cruickshank JK A review of ethnicity, health and nutrition-related diseases in relation to migration in the United Kingdom. Public Health Nutr 2001;4:647–57 11683557 10.1079/phn2001148

[29] Freathy RM Kazeem GR Morris RW Genetic variation at *CHRNA5-CHRNA3-CHRNB4* interacts with smoking status to influence BMI*.* Int J Epidemiol 2011;40:1617–28 21593077 10.1093/ije/dyr077 PMC3235017

