# Supplementary material for: Differences in socioeconomic position, lifestyle and health-related pregnancy characteristics between Pakistani and White British women in the Born in Bradford prospective cohort study: the influence of the woman's, her partner's and their parents’ place of birth
Source: BMJ Open. 2014 Jun 19;4(6):e004805. doi: 10.1136/bmjopen-2014-004805 (PMC4067825; doi:10.1136/bmjopen-2014-004805)
Supplement: Author's manuscript [file bmjopen-2014-004805.draft_revisions.pdf]

Differences in socioeconomic position, lifestyles and health related pregnancy characteristics between Pakistani and White British women: the influence of the woman's, her partner's and their parents' place of birth.

Journal:	*BMJ Open*
Manuscript ID:	bmjopen-2014-004805
Article Type:	Research
Date Submitted by the Author:	06-Jan-2014
Complete List of Authors:	West, Jane; Bradford Teaching Hospitals NHS Foundation Trust, Bradford Institute for Health Research<br>Lawlor, Debbie; Department of Social Medicine, University of Bristol, Department of Social Medicine<br>fairley, lesley; Bradford Institute for Health Research, Data management<br>Wright, John; Department of Epidemiology and Public Health, Royal Infirmary Bradford
**Primary Subject Heading**:	Epidemiology
Secondary Subject Heading:	Public health
Keywords:	EPIDEMIOLOGY, ETHNICITY, LIFESTYLE

SCHOLARONE™ Manuscripts

For peer review only

Differences in socioeconomic position, lifestyles and health related pregnancy characteristics between Pakistani and White British women: the influence of the woman's, her partner's and their parents' place of birth.

West J *Specialty Registrar in Public Health*[1], **Lawlor DA** *professor of epidemiology* [2], **Fairley L** *statistician*[1], **Wright J** *professor of clinical epidemiology*[1]

[1] Bradford Institute for Health Research, Bradford Royal Infirmary, Bradford, BD9 6RJ, UK

[2] MRC Integrative Epidemiology Unit at the University of Bristol, UK

Correspondence to jane.west@bthft.nhs.uk

For peer review only

For peer review only - http://bmjopen.bmj.com/site/about/guidelines.xhtml

ABSTRACT

Objective

To examine differences between Pakistani and White British women in relation to socioeconomic position, lifestyle characteristics and health related pregnancy characteristics, and to determine whether these differences vary depending on the woman's, her partner's and both of their parents' place of birth.

Design

Prospective cohort study.

Setting

Bradford, UK

Participants

5038 Pakistani and 4412 White British women recruited to the Born in Bradford cohort study.

Main outcome measures

Socioeconomic position (employment status; level of education; receipt of benefits; housing tenure), lifestyle characteristics (BMI at the start of pregnancy; smoking during pregnancy) and health related pregnancy characteristics (hypertensive disorders of pregnancy; gestational diabetes; fasting glucose, postload glucose and fasting insulin at ~27 weeks gestation).

Results

Pakistani women were less likely to be employed (OR 0.17 95% CI 0.15, 0.19), the difference being markedly less for UK born women. UK born Pakistani women were more likely, and South Asian born less likely, to be educated post 16 than White British women. Smoking was uncommon among Pakistani women. BMI was lower among Pakistani compared to White British women (mean difference -1.12 95% CI -1.43, -0.81) the difference greatest when partners were UK born irrespective of the woman's place of birth. Pakistani women had higher fasting and postload glucose (mean difference 0.20 95% CI 0.17, 0.24; 0.37 95% CI 0.28, 0.45).

Conclusions

Our results suggest that some socioeconomic, lifestyle and pregnancy characteristics could be beginning to change in response to migration to the UK, with potentially beneficial (e.g. greater education in women who were UK born and more adverse glucose / insulin outcome

For peer review only - http://bmjopen.bmj.com/site/about/guidelines.xhtml

For peer review only

for women whose partners were South Asian born) and detrimental (e.g. BMI levels closer to those of the White British women for those whose partners were UK born) changes.

Strengths & limitations of this study

We explored differences in socioeconomic, lifestyle and pregnancy characteristics between UK Pakistani and White British women.

We found that some differences are reduced and some are enhanced in UK born Pakistani women suggesting both positive and negative changes in response to migration.

Place of birth of both women and their partners may be important to lifestyle choices in this population.

These results may not be generalizable to other South Asian populations and further work will be important to track these differences over future generations of UK South Asian migrants.

For peer review only

For peer review only - http://bmjopen.bmj.com/site/about/guidelines.xhtml

INTRODUCTION

Migration of South Asian populations to high income countries is generally thought to offer socioeconomic advantages in the form of improved education and employment opportunities, better housing and access to health care. However, improvements in environmental circumstances do not necessarily translate into improvements in health outcomes. Indeed, South Asian migrant populations to the UK often experience significantly poorer health outcomes than the UK population as a whole[1]. This may reflect the effects of previous disadvantage associated with the country of origin which could persist over several generations, or could be a consequence of poor socioeconomic status within the host country, UK South Asian communities are on average very poor[2]. That is, it could be that in comparison to those who do not migrate, there are improved health outcomes, but these remain poorer in comparison to the indigenous population. A further explanation is that the adoption of the unhealthy and sedentary lifestyles associated with acculturation or Westernisation, characterised by lower levels of physical activity, consumption of high calorie energy rich diets and cigarette smoking, counteracts any potential health advantage of living in a higher income country. If this is the case, adoption of such lifestyles may be particularly harmful to South Asian individuals who for a given body mass index (BMI), have greater total and central adiposity and are known to be at greater risk of type 2 diabetes and cardiovascular disease than European adults[3-5].

Ethnic differences in socioeconomic position and lifestyle that might impact health during pregnancy could contribute to some of the known ethnic differences in pregnancy complications and perinatal outcomes. For example, they could contribute to the established greater risk of gestational diabetes (GDM)[6,7] and small for gestational age (SGA)[8-10] in South Asian compared to White British women[11]. They could also drive ethnic differences in future generations either through intrauterine effects of maternal behaviours on these or as a result of the adoption of parental lifestyles by offspring and a lack of social migration. Previous studies have reported ethnic differences in socioeconomic and lifestyle characteristics between South Asian and White British women during pregnancy. Findings from the Millennium Cohort Study suggest South Asian women, in particular those originating from Pakistan and Bangladesh, are less likely to have formal educational qualifications, more likely to belong to lower socioeconomic groups and more likely to have never worked or be long term unemployed[10,12]. Marked differences in smoking and alcohol consumption between South Asian and White British women have also been reported[12,13]. Whilst outside pregnancy BMI is reportedly higher among South Asian women compared to White British women[14], we have previously that BMI is lower among Pakistani origin pregnant women in the Born in Bradford (BiB) cohort[13]. Much less is known about maternal blood glucose and

For peer review only - http://bmjopen.bmj.com/site/about/guidelines.xhtml

insulin in particular whether there are differences in these outcomes across generations of UK South Asian migrants. To our knowledge, no previous studies have examined ethnic differences in all these characteristics (socioeconomic, lifestyle, pregnancy) collectively which is important to identify areas where South Asian women may have better outcomes and those where European women may have better outcomes. This knowledge could support the delivery of appropriate antenatal care aimed at maximising maternal and child health in both White British and South Asian groups.

Furthermore, previous studies have not explored whether any identified ethnic differences during pregnancy are consistent when the mother's, her partner's and both of their parents' country of origin are taken into account. In a previous study, using data from the Born in Bradford cohort, which is used in this paper, we showed that birthweight was lower, but that birth fatness (assessed using skinfold thickness and cord blood leptin) was greater in Pakistani compared to White British infants[13]. We further showed that these differences did not differ by whether both the mother and her partner and all four of their parents were born in the UK, all born in South Asia or there was a mixed pattern between these two extremes[13]. Here, we extend that work to look at a range of socioeconomic position, lifestyle and pregnancy related outcomes, in order to understand whether in the context of place of birth of women and her closest family relatives, there are some ethnic differences that are reduced or some that are enhanced, and if so whether these would be beneficial or detrimental to health.

The aim of this study is to examine differences between Pakistani women and White British women in relation to socioeconomic position (employment status; level of education; receipt of means tested benefits; housing tenure), lifestyle characteristics (BMI at the start of pregnancy; smoking during pregnancy) and health related pregnancy characteristics (hypertensive disorders of pregnancy (HDP); gestational diabetes; fasting glucose, postload glucose and fasting insulin at ~27 weeks gestation), and to determine whether these differences vary depending upon the woman's, her partner's and both of their parents' place of birth.

METHODS

Participants

The Born in Bradford (BiB) study is a largely bi-ethnic prospective birth cohort study that recruited women during pregnancy and has followed them, their infants and their partners into the child's infancy. To be eligible for the study women had to attend booking clinic between March 2007 and December 2010 and be booked to give birth in the city of

For peer review only - http://bmjopen.bmj.com/site/about/guidelines.xhtml

Bradford. Full details of the study methodology have been previously reported[15]. Women were recruited at their oral glucose tolerance test (OGTT) appointment; all women booked for delivery in Bradford are offered a 75g OGTT (comprising fasting and 2 hour postload samples) at around 26 – 28 weeks gestation. Women who attended this appointment and agreed to take part in the study consented to the use of their obstetric medical records, had their height and weight recorded and completed an interviewer administered questionnaire. The questionnaire included questions relating to ethnicity, social and economic circumstances, smoking, alcohol, diet, education and employment and collected place of birth information for both parents and all four grandparents. Interviews were conducted in a range of South Asian languages (including Mirpuri, Bengali, Punjabi). Mirpuri is the most commonly spoken Asian language in Bradford but has no written script therefore questionnaires were transliterated, that is translated verbally to Mirpuri and then written phonetically, precisely as spoken to ensure that all interpreters translated it in the same way. Details of the language used to conduct the questionnaire were recorded. Ethics approval for the study was provided by Bradford Local Research Ethics Committee (ref 06/Q1202/48). Data were available for 11,113 women recruited to the BiB cohort. We excluded stillbirths (n=64) and infants born to parents of ethnic origin other than White British or Pakistani (n=1605). Of the remaining 9451 participants 7159 had complete data for all variables included in all models thus 3656 Pakistani and 3503 White British women are included in these analyses.

Woman's family member's place of birth

Ethnicity was self-reported at interview, with participants given response options based on UK Office of National Statistics guidance[16]. Women completed a detailed ancestry interview, which included details of the place of birth of themselves, their partner and all four parents of themselves and their partner. Family place of birth groups of the Pakistani infants were derived from these data as previously reported[13]. In the previous report, since our outcome of interest was infant birth size the groups were defined in terms of 'parents' and 'grandparents'. As our outcomes here are in pregnant women we have described them in relation to her, but the groups are essentially the same as the previous paper. Briefly, for almost all women, the four parents of the woman and her partner were South Asian born meaning that the groups were based primarily on the woman's and her partner's place of birth. Overall, 90% of women fell into one of four main categories:

1. Woman and her partner UK born and all four of their parents South Asian born
2. Woman UK born, partner and all four of their parents South Asian born
3. Partner UK born, woman and all four parents South Asian born

For peer review only - http://bmjopen.bmj.com/site/about/guidelines.xhtml

4. Woman, her partner and four parents all South Asian born

The remaining 10%, including those with one or more of the woman's or her partner's parents being UK born or where their parents' place of birth was unknown, was combined to form one 'other' group.

Outcome measures

Socioeconomic

Information on socioeconomic indicators (employment, education, receipt of benefits, housing tenure) was obtained from the interview with the woman at recruitment. We equivalised the mother's highest educational qualifications (based on the qualification received and the country obtained) into one of several categories using UK NARIC (http://www.ecctis.co.uk/naric/default.aspx): *<5 GCSE equivalent, ≥5 GCSE equivalent, 'A' level equivalent, Higher than A-level equivalent, Other qualifications (e.g. City and Guilds, RSA/OCR, BTEC), Don't know, Foreign Unknown.* Don't know relates to the mother responding "don't know" during interview. Foreign Unknown relates to a qualification listed in the free text response but no level of qualification is given or the qualification listed cannot be equivalised to one of the above categories. For these analyses, women were categorised as having been educated beyond the age of 16 or not (i.e. *Higher than A-level equivalent, Other qualifications (e.g. City and Guilds, RSA/OCR, BTEC). Information* Receipt of means tested benefits was based on the mother or her household receiving any of: Income Support, Job Seekers Allowance, Working Tax Credit or Housing Benefit. Housing tenure was categorised according to whether the woman lived in a household where the home was either part-owned (i.e. mortgaged) or owned outright, or not (i.e. rented).

Lifestyle

At recruitment, women were weighed and their height measured (unshod and in light clothing) using SECA digital scales and a Leicester Height Measure respectively. Weight at first antenatal clinic assessment when women were around 12 weeks gestation (median 12 weeks, IQR 11, 14), was abstracted from the antenatal records and this weight together with height measured at recruitment, was used to calculate the woman's BMI so that this reflected early pregnancy BMI before substantial contribution from pregnancy and the growing fetus. Information on smoking was obtained at the questionnaire interview, with women categorised as having smoked cigarettes at any stage of their pregnancy or not. As none of the Pakistani origin women reported drinking alcohol, we were unable to include alcohol consumption as an outcome.

For peer review only - http://bmjopen.bmj.com/site/about/guidelines.xhtml

Health related pregnancy characteristics

Women were classified as hypertensive in pregnancy if they had a systolic measure ≥140 and a diastolic ≥90 on 2 or more occasions after 20 weeks gestation; information on this was obtained from the antenatal records. Fasting and postload glucose and fasting insulin were obtained from the OGTT samples which were assayed immediately after sampling at the biochemistry department of Bradford Royal Infirmary using the glucose oxidase method on Siemen's Advia 2400 chemistry autoanalysers. GDM was defined using the fasting and postload glucose according to WHO criteria as either a fasting glucose ≥6.1mmol/L or a two-hour postload glucose ≥7.8mmol/L. Women with existing diabetes prior to pregnancy did not complete an OGTT and are not included in this sample.

Statistical analyses

All analyses were performed using STATA (version 12.1). We used univariable regression to examine the association of ethnicity and family place of birth group with outcomes. Logistic regression was used for binary outcomes and linear regression for continuous outcomes, with the White British group used as the reference for all analyses, i.e. we compared outcomes in 'all' Pakistani women and then each of the five family place of birth subgroups of Pakistani women to outcomes in White British women. In all adjusted analyses we adjusted for maternal age and parity (Model 1). In addition to the model adjusting for maternal age and parity, for the lifestyle outcomes (early pregnancy BMI; smoking) we also adjusted for each of the indicators of socioeconomic position in order to explore the extent of any differences in these lifestyles might reflect ethnic differences in socioeconomic position (Model 2). For the health related pregnancy characteristics we also adjusted for socioeconomic indicators (Model 2) and also for the lifestyle characteristics (BMI; smoking) (Model 3), to explore whether these explained any of the differences.

RESULTS

The characteristics of White British and Pakistani origin women are shown in Table 1. There was little difference between the two ethnic groups in mean gestation, premature births and infant sex. Pakistani origin women were on average slightly older, more likely to be married and on average they lived within larger households than White British women. These differences were similar across all generation groups. Pakistani women were shorter than White British women but the difference was less when women were UK born. There were also some differences in parity across Pakistani generation groups, for example, parity was on average lowest when both parents were UK born and highest when both parents were born in South Asia.

For peer review only - http://bmjopen.bmj.com/site/about/guidelines.xhtml

Pakistani women as a whole were 83% less likely to be employed (adjusted OR 0.17 95% CI 0.15, 0.19) than White British women, but there were differences by family place of birth. Those who were South Asian born were 94% less likely to be in employment but this difference reduced to 60% for Pakistani women when both they and their partner were UK born. Following adjustment for maternal age and parity, Pakistani women as a whole were more likely to be educated beyond the age of 16 than White British women (OR 1.15 95% CI 1.04, 1.27), however there were marked differences across family place of birth groups with women who were South Asian born being less likely, and those who were UK born being more likely, to be educated beyond 16 years. Being in receipt of means tested benefits was similar in both ethnic groups when Pakistani women were assessed as a whole (adjusted OR 0.97 95% CI 0.87, 1.09) although for Pakistani women were UK born there were increased odds of receiving benefits, especially when they were UK born but their partner and parents were born in South Asia (adjusted OR 1.42 95% CI 1.20, 1.67). Compared to White British women, Pakistani women were considerably more likely to own or part own their home (adjusted OR 2.30 95% CI 2.07, 2.56) and this was consistent across all family place of birth groups.

Table 3 shows the unadjusted and adjusted (Models 1 and 2) ethnic difference in lifestyle characteristics. Pakistani women had a lower BMI than White British women (adjusted [Model 2] mean difference -1.12 95% CI -1.43, -0.81) but the difference was much greater when the woman's partner was UK born irrespective of where the woman herself was born (Figure 1). Pakistani women were around 94% less likely to smoke and this was similar across generation groups other than when both the woman and her partner were UK born in which case women were 85% less likely to have smoked during pregnancy. None of the Pakistani women reported drinking any alcohol during pregnancy (0%), whereas 8% of White British women drank during pregnancy.

In Table 4 the unadjusted and adjusted (Models 1-3) ethnic difference in pregnancy characteristics is shown. Pakistani women in general were less likely to have HDP (adjusted [Model 3] OR 0.87 95% CI 0.67, 1.13). However, this was not consistent across all family place of birth groups for example, women who were South Asian born were slightly more likely to have HPD than White British women and this was the case in all 3 adjusted models. Pakistani women were more likely to have GDM and higher fasting and postload glucose and fasting insulin than White British women and these differences were broadly similar across all 3 models of adjustment. There were some differences by family place of birth group, for example, the difference in postload glucose between Pakistani and White British women was far greater when the woman and her partner were born in South Asia than when

For peer review only - http://bmjopen.bmj.com/site/about/guidelines.xhtml

both were UK born (adjusted mean difference [Model 3] 0.57 95% CI 0.45, 0.69 and 0.18 95% CI 0.02, 0.34 respectively and Figure 2).

DISCUSSION

We have shown a number of differences in socioeconomic, lifestyle and pregnancy characteristics between Pakistani and White British origin women. We have for the first time, been able to determine whether these differences vary depending on the woman's, her partner's and both of their parents' place of birth. This provides important information about how these differences might be reduced or even enhanced with greater acculturation over generations. For example, Pakistani women as a whole, were 83% less likely to be in employment than White British women, but across generation groups this difference varied from 60% when both the woman and her partner were born in the UK, to 94% when both the woman and her partner were South Asian born. Likewise, we found interesting differences in education attainment between Pakistani and White British women. Overall, Pakistani women were slightly more likely to have been educated beyond the age of 16, but this was driven by UK born women, especially those with a UK born partner who were twice as likely as White British women to have undertaken post 16 education. By contrast, South Asian born Pakistani women, irrespective of their partner's place of birth, were less likely to have been educated after the age of 16. This could reflect a positive effect of migration and acculturation on social mobility which likely plays a part in the employment differences described above and is consistent with previous reports[12,17]. Whilst differences in employment and education by place of birth suggest the adoption of British behaviours and lifestyle, the tendency of Pakistani women to live within larger households and to be more likely to own or part-own their own home, suggests that the traditional culture of living within extended families has been maintained in this population. Living with an extended family could have considerable benefits for the mother and her offspring, such as childcare support and greater social capital, but could also result in overcrowding and potential detrimental impacts of this on health[18]. Early analyses using data from BiB suggests that living with more family members does not lead to greater family social capital. Pakistani women who were born in the UK were more likely to claim benefits than those who were South Asian born which is surprising given that they tend to be more likely to be in employment; this might reflect greater education among those who were UK born or that poorer command of the English language (likely amongst those who were South Asian born) is a barrier to accessing services and social support.

Greater social migration has been associated with increased uptake of lifestyle characteristics of the host country such as smoking and alcohol consumption (Hawkins

For peer review only - http://bmjopen.bmj.com/site/about/guidelines.xhtml

2008). We report a similar trend in that UK born Pakistani origin women were slightly more likely to smoke than South Asian born women, but smoking was still uncommon among all Pakistani women and none of them reported any alcohol consumption during pregnancy. Thus, the increase in these harmful health behaviours over generations appears minimal among Pakistani women, which may reflect persisting cultural or religious influences[19,20]. We found BMI to be slightly lower among Pakistani origin women compared to White British women although there were interesting differences across family place of birth groups. The finding that the difference in BMI between Pakistani and White British women was markedly greater for Pakistani women with a UK born partner, irrespective of their own place of birth, than for women with a South Asian born partner is particularly striking. One possible explanation is that within this population, partners/husbands have a particularly dominant role[21]. Thus, the lifestyle choices of the family or household will be driven mostly by the social norms and habits of the partner. In the case of men born in the UK, these are likely to be influenced by western culture which promotes a lower BMI as both healthy and attractive. Similarly, having been brought up and educated in the UK, they may be more likely to participate in organised physical activity and also may be more receptive to UK public health campaigns.

For peer review only

Health related pregnancy characteristics may be the most important to the long-term health of South Asian migrants in the UK, particularly in relation to the association of these characteristics with cardiovascular disease and type 2 diabetes[22]. We report a number of differences between Pakistani women and White British women in HDP, glucose tolerance, insulin and GDM. Pakistani women were less likely to have HDP and consistent with previous studies[7], but were more than twice as likely to have GDM. Consistent with higher rates of GDM, Pakistani women had higher fasting and postload glucose and higher fasting insulin than White British women and these findings are consistent with previous studies showing South Asian women are more likely to have GDM than White European women[6,7] and considerable evidence that adult non-pregnant women and men have a higher risk of insulin resistance and type 2 diabetes[3-5,22]. We found that the increased likelihood of Pakistani women having GDM compared to White British women was greatest for South Asian born women. We also found that the mean difference in fasting and postload glucose and fasting insulin relative to White British women was substantively greater when the woman and her partner were both born in South Asia. This is somewhat surprising as evidence suggests that the increased risk of insulin resistance and type 2 diabetes in South Asian adults compared to White Europeans is largely amongst those in urban (rather than rural) areas of South Asia[23], or in those who have migrated to Western countries[3,24]. We might therefore have expected the increase to be greater amongst those who were UK born.

For peer review only - http://bmjopen.bmj.com/site/about/guidelines.xhtml

The difference between our findings and those of previous studies of non-pregnant migrants, could be because most of those previous studies are in Indian rather than Pakistani migrant groups. Pakistani migrants in general tend to be poorer, shorter and weigh less, and the Pakistani women in this study have lower BMI than the White British women. For religious and cultural reasons Pakistani women are particularly unlikely to smoke or drink alcohol which does not seem to be changing in relation to place of birth in our study. It might also be that whilst insulin resistance and diabetes in the general population are enhanced in those who migrate and particularly with greater duration of migration, in pregnancy the impact of place of birth or time since migration differs. We are not aware of other studies with equivalent data to explore this further, but it would be interesting to see if this finding does replicate.

To our knowledge this is the first study to examine differences between Pakistani and White British women in relation to socioeconomic, lifestyle and pregnancy characteristics using detailed information on the place of birth of women, their partners and all four of their parents. The key strengths of this study are the large sample size, range of outcomes we have been able to examine, including OGTT data, and the detailed information on place of birth. A potential limitation of our study was the inability to include other South Asian groups in our analyses (Indian and Bangladeshi) due to small numbers within our cohort. On the one hand examining a specific South Asian population (Pakistani) reduces the problem of heterogeneity between South Asian groups but at the same time it may limit the generalisability of our results to other South Asian populations. We had hoped to explore three generations of Pakistani migrants to Bradford, but for almost all the women in this study, their parents and the parents of their partner were born in South Asia, however, this is in itself an interesting finding and useful for meeting future health needs in the city. Our analyses have not accounted for South Asians who migrate to the UK in childhood and may be resident in the UK for much of their development and education, which could potentially dilute any differences between the Pakistani place of birth groups. Within BiB information regarding the age at which an individual migrated to the UK is only available for women (not their partner or parents) therefore we were not able to account for this in our family place of birth groups.

In summary, we have found some evidence that the difference in some of these characteristics between Pakistani and White British women reduces (for example being employed and being educated beyond the age of 16) or is enhanced (for example BMI and smoking) in Pakistani women who were born in the UK. This suggests firstly, that some of these characteristics are beginning to change in response to migration to the UK and

For peer review only - http://bmjopen.bmj.com/site/about/guidelines.xhtml

secondly, that these changes can be both positive i.e. improving education and employment prospects and no evidence that being UK born has further increased the risk of GDM, and negative i.e. BMI levels closer to the higher levels of White British women and slight increases in smoking. Further work is needed that continues to track these important ethnic differences over future generations to support the delivery of appropriate antenatal care.

Words: Abstract: 369; Main text: 4157

Acknowledgements

Born in Bradford is only possible because of the enthusiasm and commitment of the children and parents in Born in Bradford. We are grateful to all participants, health professionals and researchers who have made Born in Bradford happen.

Funding

J West was funded by a UK Medical Research Council (MRC) Special Training Fellowship in Health of the Public and Health Services Research (MRC GO601712). DA Lawlor works in a unit that receives UK MRC funding (MC_UU_12013/5). BiB receives core infrastructure funding from the Wellcome Trust (WT101597MA).

Competing interests

All authors declare no competing interests

Author contributions

J West, DA Lawlor and J Wright conceived the study idea, obtained funds, developed the statistical analysis plan, were involved in managing the data collection and wrote the initial drafts of the paper; J West undertook the main analysis with input from L Fairley and supervision from DA Lawlor and J Wright. J West acts as guarantor.

Declaration of transparency

J West affirms that the manuscript is an honest, accurate, and transparent account of the study being reported; that no important aspects of the study have been omitted; and that any discrepancies from the study as planned (and, if relevant, registered) have been explained.

This is an Open Access article distributed in accordance with the Creative Commons Attribution Non Commercial (CC BY-NC 3.0) license, which permits others to distribute, remix, adapt, build upon this work non-commercially, and license their derivative works on

For peer review only - http://bmjopen.bmj.com/site/about/guidelines.xhtml

For peer review only

different terms, provided the original work is properly cited and the use is non-commercial. See:http://creativecommons.org/licenses/by-nc/3.0/.

Data sharing

Scientists are encouraged and able to use BiB data. Data requests are made to the BiB executive using the form available from the study website www.borninbradford.nhs.uk (please click on "Science and Research" to access the form). Guidance for researchers and collaborators, the study protocol and the data collection schedule are all available via the website. All requests are carefully considered and accepted where possible.

References

1. Bhala N, Zaman MS. Preventing premature mortality in chronic diseases for South Asians in the UK and beyond. *J R Soc Med* 2009;102(11): 459–463.
2. Nazroo, J. Ethnicity, class and health. London, Policy Studies Institute, 2001.
3. Barnett AH, Dixon AN, Bellary S, Hanif MW, O'Hare JP, Raymond NT, et al. Type 2 diabetes and cardiovascular risk in the UK south Asian community. *Diabetologia* 2006;49(10):2234-46.
4. Mather HM, Keen H. The Southall Diabetes survey: prevalence of known diabetes in Asians and Europeans. *BMJ* 1985;291:1081-4.
5. Gholap N, Davies M, Patel K, Sattar N, Khunti K. Type 2 diabetes and cardiovascular disease in South Asians. *Prim Care Diabetes* 2011;5(1):45-56. Epub 2010/09/28.
6. Dornhurst A, Pateson CM, Nicholls JS, Wadsworth J, Chiu DC, Johnston DG, Beard RW. High prevalence of gestational diabetes mellitus in women from ethnic minority groups. *Diabet Med* 1992; Nov;9(9):820-5
7. Makgoba M, Savvidou MD, Steer PJ. An analysis of the interrelationship between maternal age, body mass index and racial origin in the development of gestational diabetes mellitus. *BJOG* 2012; Feb;119(3):276-82.
8. Margetts BM, Mohd Yusof S, Al Dallal Z, Jackson AA. Persistence of lower birth weight in second generation South Asian babies born in the United Kingdom. *J Epidemiol Community Health* 2002 Sep;56(9):684-7.
9. Leon DA, Moser K. Low birth weight persists in South Asian babies born in England & Wales regardless of maternal country of birth. Slow pace of acculteration, physiological constraint or both? Analysis of routine data. *J Epidemiol Community Health* 2010; Published online 30 Nov 2010. doi:10.1136/jech2010.112516
10. Kelly Y, Panico L, Bartlet M, Marmot M, Nazroo J, Sacker A. Why does birthweight vary among ethnic groups in the UK? Findings from the Millennium Cohort Study. *J Public Health (Oxf)* 2008;31(1):131-137.

For peer review only - http://bmjopen.bmj.com/site/about/guidelines.xhtml

11. United Nations Children's Fund WHO. Low birthweight: country, regional and global estimates. New York, Geneva: UNICEF, WHO; 2004.

12. Hawkins SS, Lamb K, Cole TJ, Law C. Influence of moving to the UK on maternal health behaviours: prospective cohort study. *BMJ* 2008; 336:1052

13. West J; Lawlor DA; Fairley L; Bhopal R; Cameron N; McKinney PA; Sattar N; Wright J. UK born Pakistani origin infants are relatively more adipose than White British infants: findings from 8704 mother-offspring pairs in the Born in Bradford prospective birth cohort. *J Epidemiol Community Health* 2013; doi:10.1136/jech-2012-20189.1

14. Health Survey for England 2004.The health of ethnic minority groups. London, The Information Centre 2006.

15. WrightJ, Small N, Raynor P, Tuffnell DJ, Bhopal R, Cameron N, Fairley L, Lawlor DA, Parslow R, Petherick ES, Pickett KP, Waiblinger D, West J. Cohort profile: the Born in Bradford multi-ethnic family cohort study. *Int J Epidemiol* 2013; Aug;42(4):978-91.

16. Office for National Statistics (ONS). Ethnic group statistics: a guide for the collection and classification of ethnicity data. London: Stationary Office; 2003.

17. Berthaud R. Family formation in multicultural Britain: diversity and change. In Ethnicity, social mobility and public policy, Loury GC, Modood T, Teles SM (Eds).Cambridge: Cambridge University Press; 2005.

18. Golobardes B, Shaw M, Lawlor DA, Lynch JW, Davey Smith G. Indicators of socioeconomic position (Part 1). *J Epidemiol Community Health* 2006; 60(1):7-12.

19. Bradby H, Williams R. Is religion or culture the key feature in changes in substance use after leaving school? Young Punjabis and a comparison group in Glasgow. *Ethn and Health* 2006;11(3):307-324.

20. Hurcombe R, Bayley M, Goodman A. Ethnicity and alcohol. A review of the UK literature. Joseph Rowntree Foundation 2010.

21. Beishon S, Modood T, Virdee S. Ethnic minority families. London : Policy Studies Institute, 1998.

22. McKeigue PM, Pierpoint T, Ferrie JE, Marmot MG. Relationship of glucose intolerance and hyperinsulinaemia to body fat pattern in South Asians and Europeans. *Diabetologia* 1992;35(8):785-91.

23. Ebrahim S, Kinra S, Bowen L, Andersen E, Ben-Shlomo Y, Lyngdoh T, Ramakrishnan L, Ahuja RC, Joshi P, Das SM, Mohan M, Davey Smith G, Prabhakaran D, Reddy KS. The effect of rural-to-urban migration on obesity and diabetes in India: a cross-sectional study. *PLoS Med* 2010 Apr 27;7(4).

24. Landman J, Cruickshank JK. A review of ethnicity, health and nutrition-related diseases in relation to migration in the United Kingdom. *Public Health Nutr* 2001;4 (2b):647-657.

Table 1 Characteristics of women and infants (N=9450) by ethnic and generation group

For peer review only - http://bmjopen.bmj.com/site/about/guidelines.xhtml

	White British (UK & Ireland)	All Pakistani births	Pakistani sub-groups defined by place of birth of parents				
			Pakistani: Woman & partner UK born, all 4 parents South Asian born	Pakistani: Woman UK born, partner & all 4 parents South Asian born	Pakistani: Partner UK born, woman & all 4 parents South Asian born	Pakistani: Woman, partner & all 4 parents South Asian born	Pakistani: Other
**Number**	3503	3656	383	992	876	1060	345
**Gestation at delivery (weeks)** Mean (sd)	39.0 (1.9)	39.0 (1.8)	39.0 (1.8)	38.9 (1.9)	39.0 (1.9)	39.1 (1.7)	39.1 (1.6)
**Births before 37 weeks N (%)**	209 (6.0)	204 (5.6)	22 (5.7)	63 (6.4)	50 (5.7)	52 (4.9)	17 (4.9)
**Mean birth weight in gm (sd)**	3346 (568)	3124(540)	3114 (538)	3100 (549)	3101 (537)	3160 (547)	3158 (497)
**Sex N (%)**							
**Male**	1808(52)	1851(51)	200(52)	504(51)	420(48)	535(51)	192(56)
**Female**	1695(48)	1805(49)	183(48)	488(49)	456(52)	525(49)	153(44)
**Maternal age** Mean (sd)	27 (6)	28 (5)	28 (5)	28 (5)	27 (5)	30 (5)	26 (5)
**Maternal height (m) Mean (sd)**	1.64 (0.06)	1.60 (0.06)	1.61 (0.05)	1.60 (0.06)	1.59 (0.05)	1.59 (0.05)	1.61 (0.06)
**Parity N (%)**							
0	1688 (48)	1157 (32)	155 (40)	331 (33)	253 (29)	254 (24)	164 (47)
1	1122 (32)	986 (26)	105 (27)	261 (26)	253 (29)	265 (25)	102 (30)
2	454 (13)	754 (21)	76 (20)	194 (20)	199 (23)	233 (22)	52 (15)
3	139 (4)	462 (13	34 (9)	125 (13)	111 (12)	178 (17)	14 (4)
4 or more	100 (3)	297 (8)	13 (4)	81 (8)	60 (7)	130 (12)	13 (4)
**Married N (%)**	1149 (33)	3571 (98)	364 (95)	974 (98)	862 (98)	1051 (99)	320 (93)
**Living with a partner N (%)**	2518 (72)	4702 (93)	352 (92)	898 (91)	852 (97)	1001 (95)	303 (88)
**Consumed alcohol during pregnancy N (%)**	266 (8)	0 (0)	0 (0)	0 (0)	0 (0)	0 (0)	0 (0)
**Total number of household members** Mean (sd)	3 (1)	5 (3)	5 (3)	5 (2)	6 (3)	5 (2)	5 (3)

Table 2 Unadjusted and adjusted* odds ratios (95% CI) for socioeconomic characteristics for ethnic and generation groups

	**White British N=3503**	**All Pakistani births N=3656**	**Pakistani sub-groups defined by place of birth of parents**				
			**Pakistani: Woman & partner UK born, all 4 parents South Asian born N=383**	**Pakistani: Woman UK born, partner & all 4 parents South Asian born N=992**	**Pakistani: Partner UK born, woman & all 4 parents South Asian born N=876**	**Pakistani: Woman, partner & all 4 parents South Asian born N=1060**	**Pakistani: Other N=345**
**In employment**							
Unadjusted OR	**1**	**0.17 (0.16, 0.19)**	0.46 (0.37, 0.56)	0.35 (0.30, 0.40)	0.06 0.04, 0.07)	0.08 (0.06, 0.09)	0.24 (0.19, 0.30)
Adjusted OR*	**1**	**0.17 (0.15, 0.19)**	0.40 (0.32, 0.51)	0.38 (0.32, 0.44)	0.06 (0.04, 0.07)	0.06 (0.05, 0.08)	0.25 (0.19, 0.32)
**Educated post 16**							
Unadjusted OR	1	**0.90 (0.82, 0.99)**	1.89 (1.52, 2.34)	1.08 (0.94, 1.25)	0.64 (0.55, 0.74)	0.72 (0.63, 0.83)	1.06 (0.85, 1.33)
Adjusted OR*	1	**1.15 (1.04, 1.27)**	2.14 (1.70, 2.68)	1.37 (1.18, 1.59)	0.86 (0.73, 1.02)	0.88 (0.75, 1.03)	1.39 (1.11, 1.76)
**In receipt of means tested benefits†**							
Unadjusted OR	**1**	**1.48 (1.35, 1.63)**	1.20 (0.97, 1.49)	1.90 (1.64, 2.19)	1.29 (1.11, 1.49)	1.58 (1.38, 1.89)	1.05 (0.83, 1.31)
Adjusted OR*	**1**	**0.97 (0.87, 1.09)**	1.02 (0.79, 1.30)	1.42 (1.20, 1.67)	0.71 (0.60, 0.84)	0.91 (0.78, 1.08)	0.84 (0.65, 1.09)
**Housing tenure: owns or part-owns (Mortgage)**							
Unadjusted OR	**1**	**2.14 (1.94, 2.36)**	2.46 (1.94, 3.12)	2.42 (2.07, 2.83)	2.81 (2.37, 3.32)	1.67 (1.45, 1.93)	1.53 (1.21, 1.92)
Adjusted OR*	**1**	**2.30 (2.07, 2.56)**	2.49 (1.95, 3.18)	2.60 (2.20, 3.06)	3.35 (2.80, 3.99)	1.55 (1.32, 1.80)	2.02 (1.60, 2.57)

*Adjusted for maternal age; parity
† Any of: Income Support; Job Seekers Allowance; Working Tax Credit; Housing Benefits

For peer review only - http://bmjopen.bmj.com/site/about/guidelines.xhtml

Table 3 Unadjusted and adjusted* mean difference / odds ratios (95% CI) for lifestyle characteristics for ethnic and generation groups

	White British N=3503	All Pakistani births N=3656	Pakistani sub-groups defined by place of birth of parents				
			**Pakistani: Woman & partner UK born, all 4 parents South Asian born N=383**	**Pakistani: Woman UK born, partner & all 4 parents South Asian born N=992**	**Pakistani: Partner UK born, woman & all 4 parents South Asian born N=876**	**Pakistani: Woman, partner & all 4 parents South Asian born N=1060**	**Pakistani: Other N=345**
**BMI at start of pregnancy**							
Unadjusted mean difference	**0**	**-1.15 (-1.41, -0.88)**	-2.53 (-3.13, -1.94)	-0.15 (-0.55, 0.25)	-2.44 (-2.86, -2.02)	-0.43 (-0.82, -0.04)	-1.40 (-2.02, -0.77)
Adjusted mean difference: Model 1*	**0**	**-1.75 (-2.01, -1.49)**	-2.84 (-3.41, -2.26)	-0.73 (-1.12, -0.34)	-2.95 (-3.36, -2.54)	-1.49 (-1.88, -1.10)	-1.22 (-1.83, -0.62)
Adjusted mean difference: Model 2**	**0**	**-1.12 (-1.43, -0.81)**	-2.32 (-2.92, -1.72)	-0.35 (-0.76, 0.07)	-2.22 (-2.69, -1.75)	-0.99 (-1.43, -0.57)	-0.77 (-1.39, -0.15)
**Smoked during pregnancy**							
Unadjusted OR	**1**	**0.07 (0.06, 0.08)**	0.14 (0.09, 0.21)	0.09 (0.07, 0.13)	0.02 (0.01, 0.03)	0.03 (0.02, 0.05)	0.16 (0.11, 0.24)
Adjusted OR: Model 1*	**1**	**0.06 (0.05, 0.07)**	0.13 (0.09, 0.20)	0.09 (0.06, 0.12)	0.01 (0.01, 0.03)	0.03 (0.02, 0.05)	0.12 (0.08, 0.19)
Adjusted OR: Model 2**	**1**	**0.06 (0.05, 0.08)**	0.15 (0.09, 0.23)	0.09 (0.07, 0.13)	0.01 (0.01, 0.03)	0.03 (0.02, 0.05)	0.13 (0.08, 0.19)

*Adjusted for maternal age; parity
**Adjusted for maternal age; parity; employment status; level of education, receipt of means tested benefits; housing tenure

For peer review only - http://bmjopen.bmj.com/site/about/guidelines.xhtml

Table 4 Unadjusted and adjusted* mean difference / odds ratios (95% CI) for health related pregnancy characteristics for ethnic and generation groups

	White British N=3503	All Pakistani births N=3656	Pakistani sub-groups defined by place of birth of parents				
			Pakistani: Woman & partner UK born, all 4 parents South Asian born N=383	Pakistani: Woman UK born, partner & all 4 parents South Asian born N=992	Pakistani: Partner UK born, woman & all 4 parents South Asian born N=876	Pakistani: Woman, partner & all 4 parents South Asian born N=1060	Pakistani: Other N=345
**Hypertensive disorders of pregnancy**							
Unadjusted OR	**1**	**0.74 (0.61, 0.90)**	0.59 (0.35, 0.99)	0.74 (0.54, 1.01)	0.70 (0.51, 0.98)	0.91 (0.68, 1.20)	0.49 (0.27, 0.89)
Adjusted OR: Model 1*	**1**	**0.82 (0.67, 1.01)**	0.62 (0.37, 1.04)	0.82 (0.59, 1.12)	0.85 (0.61, 1.19)	0.99 (0.74, 1.33)	0.56 (0.31, 1.01)
Adjusted OR: Model 2**	**1**	**0.82 (0.64, 1.04)**	0.62 (0.36, 1.06)	0.81 (0.58, 1.13)	0.87 (0.59, 1.29)	1.01 (0.73, 1.40)	0.56 (0.31, 1.03)
Adjusted OR: Model 3***	**1**	**0.87 (0.67, 1.13)**	0.78 (0.45, 1.35)	0.80 (0.56, 1.14)	1.06 (0.70, 1.61)	1.06 (0.75, 1.49)	0.57 (0.31, 1.06)
**Gestational diabetes**							
Unadjusted OR	**1**	2.42 (2.01, 2.91)	1.65 (1.09, 2.46)	2.07 (1.59, 2.69)	2.27 (1.74, 2.96)	3.42 (2.72, 4.29)	1.78 (1.18, 2.68)
Adjusted OR: Model 1*	**1**	2.41 (1.98, 2.94)	1.66 (1.10, 2.49)	2.07 (1.58, 2.71)	2.54 (1.92, 3.35)	3.01 (2.36, 3.83)	2.24 (1.47, 3.41)
Adjusted OR: Model 2**	**1**	2.28 (1.82, 2.86)	1.66 (1.09, 2.53)	1.98 (1.49, 2.64)	2.47 (1.79, 3.39)	2.89 (2.20, 3.82)	2.21 (1.44, 3.40)
Adjusted OR: Model 3***	**1**	2.38 (1.86, 3.03)	1.89 (1.23, 2.92)	1.98 (1.46, 2.67)	2.82 (2.01, 3.97)	3.04 (2.27, 4.08)	2.29 (1.47, 3.56)

For peer review only - http://bmjopen.bmj.com/site/about/guidelines.xhtml

**Fasting glucose**							
Unadjusted mean difference	**0**	**0.20 (0.18, 0.23)**	0.13 (0.08, 0.19)	0.17 (0.14, 0.21)	0.13 (0.09, 0.17)	0.32 (0.29, 0.36)	0.19 (0.13, 0.25)
Adjusted mean difference: Model 1*	**0**	**0.18 (0.16, 0.21)**	0.12 (0.06, 0.17)	0.15 (0.11, 0.19)	0.12 (0.09, 0.16)	0.27 (0.24, 0.31)	0.22 (0.16, 0.27)
Adjusted mean difference: Model 2**	**0**	**0.18 (0.15, 0.21)**	0.12 (0.06, 0.18)	0.15 (0.11, 0.19)	0.12 (0.07, 0.16)	0.27 (0.23, 0.31)	0.22 (0.16, 0.27)
Adjusted mean difference: Model 3***	**0**	**0.20 (0.17, 0.24)**	0.17 (0.12, 0.23)	0.16 (0.12, 0.19)	0.17 (0.12, 0.21)	0.29 (0.25, 0.33)	0.23 (0.17, 0.29)
**Postload glucose**							
Unadjusted mean difference	**0**	**0.42 (0.35, 0.49)**	0.12 (-0.04, 0.28)	0.34 (0.23, 0.45)	0.35 (0.24, 0.46)	0.72 (0.62, 0.83)	0.26 (0.09, 0.42)
Adjusted mean difference: Model 1*	**0**	**0.37 (0.29, 0.44)**	0.08 (-0.07, 0.24)	0.29 (0.18, 0.39)	0.35 (0.24, 0.46)	0.58 (0.48, 0.69)	0.35 (0.19, 0.51)
Adjusted mean difference: Model 2**	**0**	**0.35 (0.27, 0.43)**	0.10 (-0.06, 0.26)	0.28 (0.17, 0.39)	0.33 (0.20, 0.46)	0.56 (0.44, 0.68)	0.34 (0.18, 0.51)
Adjusted mean difference: Model 3***	**0**	**0.37 (0.28, 0.45)**	0.18 (0.02, 0.34)	0.27 (0.16, 0.38)	0.39 (0.26, 0.52)	0.57 (0.45, 0.69)	0.35 (0.18, 0.52)
**Fasting insulin**							
Unadjusted mean difference	**0**	**18.88 (16.31, 21.45)**	11.26 (5.42, 17.09)	19.36 (15.46, 23.26)	10.36 (6.26, 14.45)	24.71 (20.91, 28.51)	29.69 (23.58, 35.81)
Adjusted mean difference: Model 1*	**0**	**18.08 (15.42, 20.74)**	10.98 (5.13, 16.82)	18.59 (14.64, 22.54)	9.67 (5.51, 13.83)	23.36 (19.43, 27.30)	29.69 (23.55, 35.82)
Adjusted mean difference: Model 2**	**0**	**21.29 (18.13, 24.45)**	14.01 (7.95, 20.08)	20.62 (16.40, 24.83)	13.53 (8.73, 18.34)	25.24 (20.89, 29.59)	32.01 (25.72, 38.31)
Adjusted mean difference: Model 3***	**0**	**25.71 (22.73, 28.69)**	24.44 (19.03, 29.86)	21.29 (17.47, 25.13)	23.27 (18.86, 27.68)	29.03 (25.04, 33.02)	34.79 (29.18, 40.39)

*Adjusted for maternal age; parity
**Adjusted for maternal age; parity; employment status; level of education, receipt of means tested benefits; housing tenure
*** Adjusted for maternal age; parity; employment status; level of education, receipt of means tested benefits; housing tenure; early pregnancy BMI; smoking in pregnancy

For peer review only - http://bmjopen.bmj.com/site/about/guidelines.xhtml

Figure 1 Adjusted mean differences in BMI for Pakistani women relative to White British women

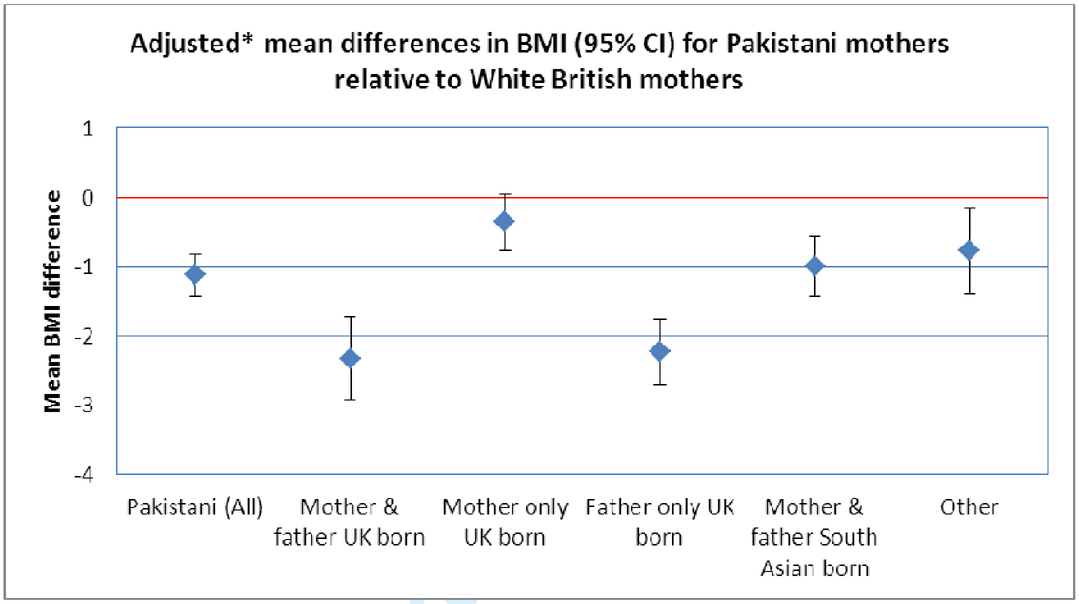

*Model 2: Adjusted for maternal age; parity; employment; post-16 education; receipt of means tested benefits; housing tenure

Figure 2 Adjusted mean differences in fasting insulin for Pakistani women relative to White British women

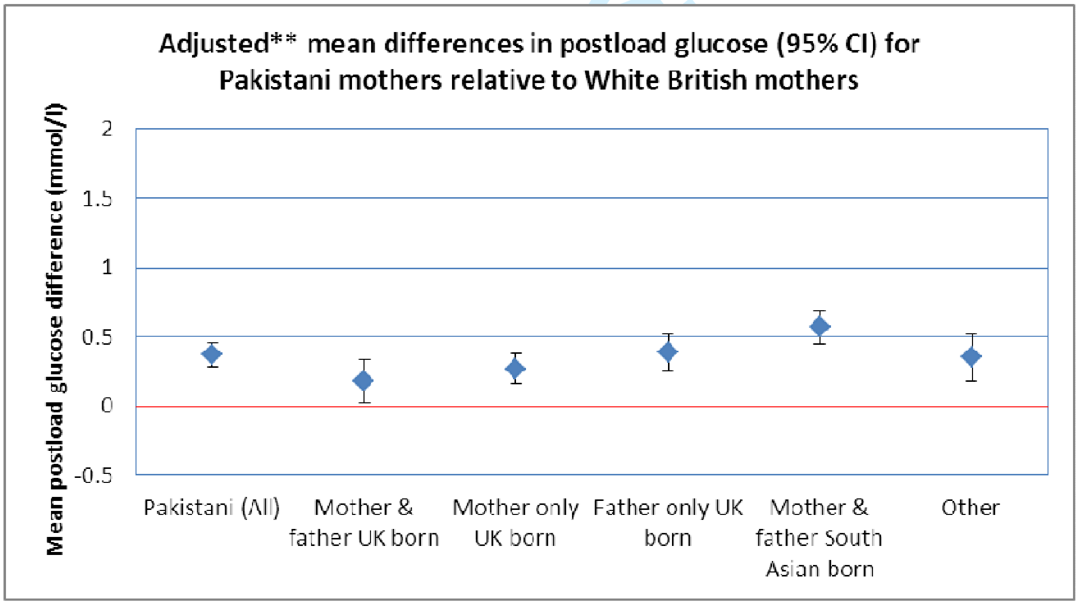

**Model 3: Adjusted for maternal age; parity; employment; post-16 education; receipt of means tested benefits; housing tenure; early pregnancy BMI; smoking in pregnancy; alcohol in pregnancy

STROBE Statement—Checklist of items that should be included in reports of ***cohort studies***

	Item No	Recommendation
**Title and abstract**	1	(*a*) The study's design is indicated in the title or the abstract (page 2)
		(*b*) Informative and balanced summary provided in abstract (page 2)
**Introduction**		
Background/rationale	2	Scientific background and rationale for the investigation being reported explained (page 4-6)
Objectives	3	Specific objectives stated (page 5)
**Methods**		
Study design	4	Key elements of study design presented (pages 6 & 7)
Setting	5	The setting, locations, and relevant dates, including periods of recruitment, exposure, follow-up, and data collection described (pages 6 & 7)
Participants	6	(*a*) Eligibility criteria and methods of follow-up given (page 6)
		(*b*) For matched studies, give matching criteria and number of exposed and unexposed N/A
Variables	7	All outcomes, exposures, predictors, potential confounders, and effect modifiers clearly defined (page 6 & 7)
Data sources/ measurement	8*	Sources of data and details of methods of assessment given. (pages 6 & 7)
Bias	9	Potential sources of bias discussed (page 12)
Study size	10	Study size described (page 6)
Quantitative variables	11	Means and sd /medians IQR were reported for continuous variables (pages 8 & 9)
Statistical methods	12	(*a*) All statistical methods, including those used to control for confounding described (page 7)
		(*b*) Describe any methods used to examine subgroups and interactions N/A
		(*c*) Explain how missing data were addressed : N/A
		(*d*) If applicable, explain how loss to follow-up was addressed N/A
		(*e*) Describe any sensitivity analyses N/A
**Results**		
Participants	13*	(a) Report numbers of individuals at each stage of study—eg numbers potentially eligible, examined for eligibility, confirmed eligible, included in the study, completing follow-up, and analysed (page 6)
		(b) Give reasons for non-participation at each stage N/A
		(c) Consider use of a flow diagram – described in methods
Descriptive data	14*	(a) Give characteristics of study participants (eg demographic, clinical, social) and information on exposures and potential confounders: included on page 6
		(b) Indicate number of participants with missing data for each variable of interest: N/A
		(c) Summarise follow-up time (eg, average and total amount) N/A (birth data)
Outcome data	15*	Report numbers of outcome events or summary measures over time: outcomes reported in results pages 8 & 9
Main results	16	(*a*) Give unadjusted estimates and, if applicable, confounder-adjusted estimates and their precision (eg, 95% confidence interval). Make clear which confounders were adjusted for and why they were included: included in main manuscript and tables
		(*b*) Report category boundaries when continuous variables were categorized: N/A
		(*c*) If relevant, consider translating estimates of relative risk into absolute risk for a

For peer review only

		meaningful time period N/A
Other analyses	17	Report other analyses done—eg analyses of subgroups and interactions, and sensitivity analyses N/A
**Discussion**		
Key results	18	Key results with reference to study objectives summarised (page 10)
Limitations	19	Limitations of the study, taking into account sources of potential bias or imprecision discussed. Limitations discussed (page 12)
Interpretation	20	Give a cautious overall interpretation of results considering objectives, limitations, multiplicity of analyses, results from similar studies, and other relevant evidence: included in discussion (pages10-12)
Generalisability	21	Discuss the generalisability (external validity) of the study results: included in discussion (page 12)
**Other information**		
Funding	22	Sources of funding and the role of the funders for the present study included (at end of manuscript)

For peer review only

For peer review only - http://bmjopen.bmj.com/site/about/guidelines.xhtml

BMJ Open

Differences in socioeconomic position, lifestyles and health related pregnancy characteristics between Pakistani and White British women: the influence of the woman's, her partner's and their parents' place of birth.

Journal:	*BMJ Open*
Manuscript ID:	bmjopen-2014-004805.R1
Article Type:	Research
Date Submitted by the Author:	08-Apr-2014
Complete List of Authors:	West, Jane; Bradford Teaching Hospitals NHS Foundation Trust, Bradford Institute for Health Research<br>Lawlor, Debbie; Department of Social Medicine, University of Bristol, Department of Social Medicine<br>fairley, lesley; Bradford Institute for Health Research, Data management<br>Wright, John; Department of Epidemiology and Public Health, Royal Infirmary Bradford
**Primary Subject Heading**:	Epidemiology
Secondary Subject Heading:	Public health
Keywords:	EPIDEMIOLOGY, ETHNICITY, LIFESTYLE

SCHOLARONE™
Manuscripts

For peer review only

Differences in socioeconomic position, lifestyles and health related pregnancy characteristics between Pakistani and White British women: the influence of the woman's, her partner's and their parents' place of birth.

West J *Specialty Registrar in Public Health*[1], **Lawlor DA** *professor of epidemiology* [2,3], **Fairley L** *statistician*[1], **Wright J** *professor of clinical epidemiology*[1]

[1] Bradford Institute for Health Research, Bradford Royal Infirmary, Bradford, BD9 6RJ, UK

[2] MRC Integrative Epidemiology Unit at the University of Bristol, UK

[3]School of Social and Community Medicine, University of Bristol, UK

Correspondence to jane.west@bthft.nhs.uk

For peer review only

For peer review only - http://bmjopen.bmj.com/site/about/guidelines.xhtml

ABSTRACT

Objective

To examine differences between Pakistani and White British women in relation to socioeconomic position, lifestyle characteristics and health related pregnancy characteristics, and to determine whether these differences vary depending on the woman's, her partner's and both of their parents' place of birth.

Design

Prospective cohort study.

Setting

Bradford, UK

Participants

3656 Pakistani and 3503 White British women recruited to the Born in Bradford study.

Main outcome measures

Socioeconomic position (employment status; level of education; receipt of benefits; housing tenure), lifestyle characteristics (BMI at the start of pregnancy; smoking during pregnancy) and health related pregnancy characteristics (hypertensive disorders of pregnancy; gestational diabetes; fasting glucose, postload glucose and fasting insulin at ~27 weeks gestation).

Results

Pakistani women were less likely to be employed (OR 0.17 95% CI 0.15, 0.19), the difference being markedly less for UK born women. UK born Pakistani women were more likely, and South Asian born less likely, to be educated post 16 than White British women. Smoking was uncommon among Pakistani women, though the difference comparing UK born Pakistani women to White British women was less than for other groups. BMI was lower among Pakistani compared to White British women (adjusted mean difference -1.12 95% CI -1.43, -0.81) the difference greatest when partners were UK born irrespective of the woman's place of birth. Pakistani women had higher fasting and postload glucose (mean difference 0.20 mmol/l 95% CI 0.17, 0.24; 0.37 95% CI 0.28, 0.45), higher fasting insulin and were more likely to have gestational diabetes.

For peer review only - http://bmjopen.bmj.com/site/about/guidelines.xhtml

Conclusions

Our results suggest that some socioeconomic, lifestyle and pregnancy characteristics could be beginning to change in response to migration to the UK, with generally beneficial i.e. improving education and employment prospects, lower BMI and no evidence that being UK born has further increased the risk of GDM, but some negative i.e. slight increases in smoking.

Strengths & limitations of this study

The strengths of this study include a large sample size, range of outcomes including oral glucose tolerance test data and detailed ancestry information.

We have for the first time, been able to examine ethnic differences in socioeconomic, lifestyle and pregnancy characteristics using information on the place of birth of women and their partners. We had also set out to explore differences based on all four grandparents but once we began analysing data it was apparent that for the majority of Pakistani women and their partners, all four of their parents were South Asian born. This limited our ability to explore differences across two generations, but highlights the persistence of strong family links in this community that have lived in Bradford for over 6 decades.

A potential limitation is that our results may not be generalizable to other South Asian populations and further work will be important to track these differences over future generations of UK South Asian migrants.

For peer review only

For peer review only - http://bmjopen.bmj.com/site/about/guidelines.xhtml

INTRODUCTION

Migration of South Asian populations to high income countries is generally thought to offer socioeconomic advantages in the form of improved education and employment opportunities, better housing and access to health care. However, improvements in environmental circumstances do not necessarily translate into improvements in health outcomes. Indeed, South Asian migrant populations to the UK experience an increased risk of maternal[1] and infant mortality[2] and some chronic diseases[3] compared with the UK population as a whole. This may reflect the effects of previous disadvantage associated with the country of origin which could persist over several generations, or could be a consequence of poor socioeconomic status within the host country, UK South Asian communities are on average very poor[4]. That is, it could be that in comparison to those who do not migrate, there are improved health outcomes, but these remain poorer in comparison to the indigenous population. A further explanation is that the adoption of the unhealthy and sedentary lifestyles associated with acculturation or Westernisation, often characterised by low levels of physical activity[5], consumption of high calorie energy rich diets[6] and cigarette smoking[7,8], counteracts any potential health advantage of living in a higher income country. This may vary across different migrant communities but where this is the case, adoption of such lifestyles may be particularly harmful to South Asian individuals who for a given body mass index (BMI), have greater total and central adiposity and are known to be at greater risk of type 2 diabetes and cardiovascular disease than European adults[9-11].

Ethnic differences in socioeconomic position and lifestyle that might impact health during pregnancy could contribute to some of the known ethnic differences in pregnancy complications and perinatal outcomes. For example, they could contribute to the established greater risk of gestational diabetes (GDM)[12,13] and small for gestational age (SGA)[14-16] in South Asian compared to White British women. They could also drive ethnic differences in future generations either through intrauterine effects of maternal behaviours on these or as a result of the adoption of parental lifestyles by offspring and a lack of social migration. Previous studies have reported ethnic differences in socioeconomic and lifestyle characteristics between South Asian and White British women during pregnancy. Findings from the Millennium Cohort Study suggest South Asian women, in particular those originating from Pakistan and Bangladesh, are less likely to have formal educational qualifications, more likely to belong to lower socioeconomic groups and more likely to have never worked or be long term unemployed[7,16]. Marked differences in smoking and alcohol consumption between South Asian and White British women have also been reported[7,17]. Whilst outside pregnancy BMI is reportedly higher among South Asian women compared to White British women[18], we have previously reported that BMI is lower among Pakistani origin

For peer review only - http://bmjopen.bmj.com/site/about/guidelines.xhtml

pregnant women in the Born in Bradford (BiB) cohort[17]. Much less is known about maternal blood glucose and insulin in particular whether there are differences in these outcomes across generations of UK South Asian migrants. To our knowledge, no previous studies have examined ethnic differences in all these characteristics (socioeconomic, lifestyle, pregnancy) collectively which is important to identify areas where South Asian women may have better outcomes and those where European women may have better outcomes. This knowledge could support the delivery of appropriate antenatal care aimed at maximising maternal and child health in both White British and South Asian groups.

Furthermore, previous studies have not explored whether any identified ethnic differences during pregnancy are consistent when the mother's, her partner's and both of their parents' country of origin are taken into account. In a previous study, using data from the Born in Bradford cohort, which is used in this paper, we showed that birthweight was lower, but that birth fatness (assessed using skinfold thickness and cord blood leptin) was greater in Pakistani compared to White British infants[17]. We further showed that these differences did not differ by whether both the mother and her partner and all four of their parents were born in the UK, all born in South Asia or there was a mixed pattern between these two extremes[17]. Here, we extend that work to look at a range of socioeconomic position, lifestyle and pregnancy related outcomes, in order to understand whether in the context of place of birth of women and her closest family relatives, there are some ethnic differences that are reduced or some that are enhanced, and if so whether these would be beneficial or detrimental to health.

The aim of this study is to examine differences between Pakistani women and White British women in relation to socioeconomic position (employment status; level of education; receipt of means tested benefits; housing tenure), lifestyle characteristics (BMI at the start of pregnancy; smoking during pregnancy) and health related pregnancy characteristics (hypertensive disorders of pregnancy (HDP); gestational diabetes; fasting glucose, postload glucose and fasting insulin at ~27 weeks gestation), and to determine whether these differences vary depending upon the woman's, her partner's and both of their parents' place of birth.

METHODS

Participants

The Born in Bradford (BiB) study is a largely bi-ethnic prospective birth cohort study that recruited women during pregnancy and has followed them, their infants and their partners into the child's infancy. To be eligible for the study women had to attend booking clinic

For peer review only - http://bmjopen.bmj.com/site/about/guidelines.xhtml

between March 2007 and December 2010 and be booked to give birth in the city of Bradford. Full details of the study methodology have been previously reported[18]. Women were recruited at their oral glucose tolerance test (OGTT) appointment; all women booked for delivery in Bradford are offered a 75g OGTT (comprising fasting and 2 hour postload samples) at around 26 – 28 weeks gestation. Women who attended this appointment and agreed to take part in the study consented to the use of their obstetric medical records, had their height and weight recorded and completed an interviewer administered questionnaire. The questionnaire included questions relating to ethnicity, social and economic circumstances, smoking, alcohol, diet, education and employment and collected place of birth information for both parents and all four grandparents. Interviews were conducted in a range of South Asian languages (including Mirpuri, Bengali, Punjabi). Mirpuri is the most commonly spoken Asian language in Bradford but has no written script therefore questionnaires were transliterated, that is translated verbally to Mirpuri and then written phonetically, precisely as spoken to ensure that all interpreters translated it in the same way. Details of the language used to conduct the questionnaire were recorded. Ethics approval for the study was provided by Bradford Local Research Ethics Committee (ref 06/Q1202/48). Data were available for 11,113 women recruited to the BiB cohort. We excluded stillbirths (n=64) and infants born to parents of ethnic origin other than White British or Pakistani (n=1598). Of the remaining 9451 participants 7159 had complete data for all variables included in all models thus 3656 Pakistani and 3503 White British women are included in these analyses. Women with existing diabetes (0.5% of the BiB cohort) are not invited to attend for the glucose tolerance test as they are treated from the start of their pregnancy by an endocrine physician. This means that these women were not recruited at the same time as other participants and do not have some data, including parental place of birth, therefore these women are not included in these complete case analyses.

Woman's family member's place of birth

Ethnicity was self-reported at interview, with participants given response options based on UK Office of National Statistics guidance[19]. Women completed a detailed ancestry interview, which included details of the place of birth of themselves, their partner and all four parents of themselves and their partner. Family place of birth groups of the Pakistani infants were derived from these data as previously reported[17]. In the previous report, since our outcome of interest was infant birth size the groups were defined in terms of 'parents' and 'grandparents'. As our outcomes here are in pregnant women we have described them in relation to her, but the groups are essentially the same as the previous paper. Our aim in that previous paper, as here, was to examine differences across all possible groups based on place of birth of the woman, her partner and all four parents. Thus, we began by

For peer review only - http://bmjopen.bmj.com/site/about/guidelines.xhtml

determining numbers in all 64 possible combinations of these six family members. Having done that it was apparent that for almost all women, the four parents of the woman and her partner were South Asian born meaning that the analyses were based primarily on the woman's and her partner's place of birth. Overall, 90% of women fell into one of four main categories:

1. Woman and her partner UK born and all four of their parents South Asian born
2. Woman UK born, partner and all four of their parents South Asian born
3. Partner UK born, woman and all four parents South Asian born
4. Woman, her partner and four parents all South Asian born

The remaining 11% (n=345), including those with one or more of the woman's or her partner's parents being UK born or where their parents' place of birth was unknown, was combined to form one 'other' group.

Outcome measures

Socioeconomic

Information on socioeconomic indicators (employment, education, receipt of benefits, housing tenure) was obtained from the interview with the woman at recruitment. We equivalised the mother's highest educational qualifications (based on the qualification received and the country obtained) into one of several categories using UK NARIC (http://www.ecctis.co.uk/naric/default.aspx): *<5 GCSE equivalent, ≥5 GCSE equivalent, 'A' level equivalent, Higher than A-level equivalent, Other qualifications (e.g. City and Guilds, RSA/OCR, BTEC), Don't know, Foreign Unknown.* Don't know relates to the mother responding "don't know" during interview. Foreign Unknown relates to a qualification listed in the free text response but no level of qualification is given or the qualification listed cannot be equivalised to one of the above categories. For these analyses, women were categorised as having been educated beyond the age of 16 or not (i.e. *Higher than A-level equivalent, Other qualifications (e.g. City and Guilds, RSA/OCR, BTEC). Information* Receipt of means tested benefits was based on the mother or her household receiving any of: Income Support, Job Seekers Allowance, Working Tax Credit or Housing Benefit. Housing tenure was categorised according to whether the woman lived in a household where the home was either part-owned (i.e. mortgaged) or owned outright, or not (i.e. rented).

Lifestyle

BMI is used in these analyses as a proxy marker of lifestyle as it is an outcome that can potentially be influenced by changes or differences in lifestyle (in particular dietary choices

For peer review only - http://bmjopen.bmj.com/site/about/guidelines.xhtml

and levels of physical activity). At recruitment, women were weighed and their height measured (unshod and in light clothing) using SECA digital scales and a Leicester Height Measure respectively. Weight at first antenatal clinic assessment when women were around 12 weeks gestation (median 12 weeks, IQR 11, 14), was abstracted from the antenatal records and this weight together with height measured at recruitment, was used to calculate the woman's BMI so that this reflected early pregnancy BMI before substantial contribution from pregnancy and the growing fetus. Information on smoking was obtained at the questionnaire interview, with women categorised as having smoked cigarettes at any stage of their pregnancy or not. As none of the Pakistani origin women reported drinking alcohol, we were unable to include alcohol consumption as an outcome.

Health related pregnancy characteristics

Women were classified as hypertensive in pregnancy if they had a systolic measure ≥140 and a diastolic ≥90mmHg on 2 or more occasions after 20 weeks gestation; information on this was obtained from the antenatal records. Fasting and postload glucose and fasting insulin were obtained from the OGTT plasma samples which were assayed immediately after sampling at the biochemistry department of Bradford Royal Infirmary using the glucose oxidase method on Siemen's Advia 2400 chemistry autoanalysers. GDM was defined using the fasting and postload glucose according to WHO criteria[20] at the time these women were pregnant as either a fasting glucose ≥6.1mmol/L or a two-hour postload glucose ≥7.8mmol/L. Women with existing diabetes prior to pregnancy did not complete an OGTT and are not included in this sample.

Statistical analyses

All analyses were performed using Stata (version 12.1). We used univariable regression to examine the association of ethnicity and family place of birth group with outcomes. Logistic regression was used for binary outcomes and linear regression for continuous outcomes, with the White British group used as the reference for all analyses, i.e. we compared outcomes in 'all' Pakistani women and then each of the five family place of birth subgroups of Pakistani women to outcomes in White British women. The rationale for this is because our aim is primarily to compare all Pakistani origin women with White British women and then to compare subgroups based on place of birth with the same reference group of White British women to see if place of birth of the Pakistani women influences the extent to which they differ or not from the indigenous population. In all adjusted analyses we adjusted for maternal age and parity (Model 1). In addition to the model adjusting for maternal age and parity, for the lifestyle outcomes (early pregnancy BMI; smoking) we also adjusted for each of the indicators of socioeconomic position in order to explore the extent of any differences

For peer review only - http://bmjopen.bmj.com/site/about/guidelines.xhtml

in these lifestyles might reflect ethnic differences in socioeconomic position (Model 2). For the health related pregnancy characteristics we also adjusted for socioeconomic indicators (Model 2) and also for the lifestyle characteristics (BMI; smoking) (Model 3), to explore whether these explained any of the differences.

RESULTS

The characteristics of White British and Pakistani origin women are shown in Table 1. There was little difference between the two ethnic groups in mean gestation, premature births and infant sex. As reported in our previous paper[17], birthweight of their infant was markedly lower in Pakistani compared to White British women when all Pakistani origin women were combined and also when compared by subgroups based on place of birth. Pakistani origin women were on average slightly older, in particular when both parents were South Asian born, markedly more likely to be married and on average they lived within larger households than White British women. These differences were similar across all generation groups. Pakistani women were shorter than White British women but the difference was less when women were UK born. There were also some differences in parity across Pakistani generation groups, for example, parity was on average lowest when both parents were UK born and highest when both parents were born in South Asia.

Pakistani women as a whole were 83% less likely to be employed (adjusted OR 0.17 95% CI 0.15, 0.19) than White British women, but there were differences by family place of birth (Table 2). Those who were South Asian born were 94% less likely to be in employment but this difference reduced to 60% for Pakistani women when both they and their partner were UK born. Following adjustment for maternal age and parity, Pakistani women as a whole were more likely to be educated beyond the age of 16 than White British women (OR 1.15 95% CI 1.04, 1.27), however there were marked differences across family place of birth groups with women who were South Asian born being less likely, and those who were UK born being more likely compared to White British women, to be educated beyond 16 years. Being in receipt of means tested benefits was similar in both ethnic groups when Pakistani women were assessed as a whole (adjusted OR 0.97 95% CI 0.87, 1.09) although for Pakistani women who were UK born with a South Asian partner there were increased odds of receiving benefits. Compared to White British women, Pakistani women were considerably more likely to own or part own their home (adjusted OR 2.30 95% CI 2.07, 2.56) and this was consistent across all family place of birth groups.

Table 3 shows the unadjusted and adjusted (Models 1 and 2) ethnic difference in lifestyle characteristics. Pakistani women had a lower BMI than White British women (adjusted

For peer review only - http://bmjopen.bmj.com/site/about/guidelines.xhtml

[Model 2] mean difference -1.12 95% CI -1.43, -0.81) but the difference was much greater when the woman's partner was UK born irrespective of where the woman herself was born (Figure 1). Pakistani women were around 94% less likely to smoke and this was similar across generation groups other than when both the woman and her partner were UK born in which case women were 85% less likely to have smoked during pregnancy. None of the Pakistani women reported drinking any alcohol during pregnancy (0%), whereas 8% of White British women drank during pregnancy.

In Table 4 the unadjusted and adjusted (Models 1-3) ethnic difference in pregnancy characteristics is shown. Pakistani women in general were less likely to have HDP (adjusted [Model 3] OR 0.87 95% CI 0.67, 1.13), although this result was imprecisely estimated with wide confidence intervals that included the null. This was not consistent across all family place of birth groups for example, women who were South Asian born were slightly more likely to have HPD than White British women and this was the case in all 3 adjusted models. Pakistani women were more likely to have GDM and higher fasting and postload glucose and fasting insulin than White British women and these differences were broadly similar across all 3 models of adjustment. There were some differences by family place of birth group, for example, the difference in postload glucose between Pakistani and White British women was far greater when the woman and her partner were born in South Asia than when both were UK born (adjusted mean difference [Model 3] 0.57 95% CI 0.45, 0.69 and 0.18 95% CI 0.02, 0.34 respectively and Figure 2).

DISCUSSION

We have shown differences across a range of socioeconomic, lifestyle and pregnancy characteristics between Pakistani and White British origin women and that these vary depending on whether Pakistani women are born in the UK or South Asia. We have for the first time, been able to consider not only the woman's place of birth, but also her partner's and both of their parents' place of birth; though after preliminary analyses it was clear that for the majority of women and their partners, all four of their parents were South Asian born. This provides important information about how these differences might be reduced or even enhanced with greater acculturation over generations. For example, Pakistani women as a whole, were 83% less likely to be in employment than White British women, but across generation groups this difference varied from 60% when both the woman and her partner were born in the UK, to 94% when both the woman and her partner were South Asian born. Likewise, we found interesting differences in education attainment between Pakistani and White British women. Overall, Pakistani women were slightly more likely to have been educated beyond the age of 16, but this was driven by UK born Pakistani women, especially

For peer review only - http://bmjopen.bmj.com/site/about/guidelines.xhtml

those with a UK born partner who were twice as likely as White British women to have completed education beyond age 16. By contrast, South Asian born Pakistani women, irrespective of their partner's place of birth, were less likely than White British women to have been educated beyond the age of 16. This could reflect a positive effect of migration and acculturation on social mobility which likely plays a part in the employment differences described above and is consistent with previous reports[7,21]. Whilst differences in employment and education by place of birth suggest the adoption of some British lifestyle characteristics, the tendency of Pakistani women to live within larger households and to be more likely to own or part-own their own home, suggests that the traditional culture of living within extended families has been maintained across all place of birth sub-groups of Pakistani women.. Living with an extended family could have considerable benefits for the mother and her offspring, such as childcare support and greater social capital, but could also result in overcrowding and potential detrimental impacts of this on health[22]. Early analyses using data from BiB suggests that living with more family members does not lead to greater family social capital (Cabieses B, unpublished data 2013). Pakistani women who were born in the UK but had a South Asian born partner, were more likely to claim benefits compared to White British women than those who were South Asian born which is surprising given that they tend to be more likely to be in employment. This might reflect a tendency for South Asian born partners to be in lower paid employment reducing total household income, or that poorer command of the English language (likely amongst those Pakistani women who were South Asian born and were less likely to claim benefits compared to White British women) is a barrier to accessing services and social support.

Greater social migration, for example coming to the UK for social reasons, has been associated with increased uptake of lifestyle characteristics of the host country such as smoking and alcohol consumption (Hawkins 2008). We report a similar trend in that UK born Pakistani origin women were more likely to smoke than South Asian born women, but smoking was still uncommon among all Pakistani women compared to White British women and none of them reported any alcohol consumption during pregnancy. Thus, the increase in these harmful health behaviours over generations in some migrant groups, whilst showing some signs of change, appears minimal among Pakistani women. This may reflect persisting cultural or religious influences[23,24] and could be related to the fact that for the majority of women, both of their parents and their partners parents were South Asian born We found BMI to be slightly lower among Pakistani origin women compared to White British women although there were interesting differences across family place of birth groups. The finding that the difference in BMI between Pakistani and White British women was markedly greater for Pakistani women with a UK born partner, irrespective of their own place of birth, than for

For peer review only - http://bmjopen.bmj.com/site/about/guidelines.xhtml

women with a South Asian born partner is particularly striking. One possible explanation is that within this population, partners/husbands have a particularly dominant role[25]. Thus, the lifestyle choices of the family or household will be driven mostly by the social norms and habits of the partner. In the case of men born in the UK, these are likely to be influenced by western culture which promotes a lower BMI as both healthy and attractive. Similarly, having been brought up and educated in the UK, they may be more likely to participate in organised physical activity and also may be more receptive to UK public health campaigns.

Health related pregnancy characteristics may be the most important to the long-term health of South Asian migrants in the UK, particularly in relation to the association of these characteristics with cardiovascular disease and type 2 diabetes[26]. We report a number of differences between Pakistani women and White British women in HDP, glucose tolerance, fasting insulin and GDM. Pakistani women as a whole group were less likely to have HDP, although this was not consistent across family subgroups, but more than twice as likely to have GDM. Consistent with these higher rates of GDM, Pakistani women had higher fasting and postload glucose and higher fasting insulin than White British women. These findings are similar to those from previous studies showing South Asian women are more likely to have GDM than White European women[12,13] and considerable evidence that adult non-pregnant women and men have a higher risk of insulin resistance and type 2 diabetes[9-11,26]. We found that the increased likelihood of Pakistani women having GDM compared to White British women was greatest for South Asian born women. We also found that the mean difference in fasting and postload glucose and fasting insulin relative to White British women was substantively greater when the woman and her partner were both born in South Asia. This is somewhat surprising as evidence suggests that the increased risk of insulin resistance and type 2 diabetes in South Asian adults compared to White Europeans is largely amongst those in urban (rather than rural) areas of South Asia[27], or in those who have migrated to Western countries[9,28]. We might therefore have expected the increase to be greater amongst those who were UK born. The difference between our findings and these previous studies of non-pregnant migrants[,9,26,27], might be explained by differences in the population studied, with many of these previous studies being of Indian, or mixed rather than Pakistani origin. Pakistani migrants in general tend to be poorer, shorter and weigh less, and the Pakistani women in this study have lower BMI than the White British women. For religious and cultural reasons Pakistani women remain unlikely to smoke or drink alcohol, this might influence their glucose tolerance, though smoking is related to lower BMI and so would be expected to reduce glucose tolerance[29]. It might also be that whilst insulin resistance and diabetes in the general population are enhanced in those who migrate and particularly with greater duration of migration, in pregnancy the impact of place of birth or

For peer review only - http://bmjopen.bmj.com/site/about/guidelines.xhtml

time since migration differs. We are not aware of other studies with equivalent data to explore this further, but it would be interesting to see if this finding does replicate.

The key strengths of this study are the large sample size, range of outcomes we have been able to examine, including OGTT data, and the detailed information on place of birth. To our knowledge this is the first study to examine differences between Pakistani and White British women in relation to socioeconomic, lifestyle and pregnancy characteristics using detailed information on the place of birth of women and their partners. We had hoped to explore three generations of Pakistani migrants to Bradford, but for almost all the women in this study, their parents and the parents of their partner were born in South Asia. However, this is in itself an interesting finding and useful for meeting future health needs in the city. It might also explain some of our findings in relation to the persistence of some characteristics across family place of birth subgroups. A potential limitation of our study was the inability to include other South Asian groups in our analyses (Indian and Bangladeshi) due to small numbers within our cohort. On the one hand examining a specific South Asian population (Pakistani) reduces the problem of heterogeneity between South Asian groups but at the same time it may limit the generalisability of our results to other South Asian populations. Our analyses have not accounted for South Asians who migrate to the UK in childhood and may be resident in the UK for much of their development and education, which could potentially dilute any differences between the Pakistani place of birth groups. Within BiB information regarding the age at which an individual migrated to the UK is only available for women (not their partner or parents) therefore we were not able to account for this in our family place of birth groups. We were not able to validate self-report of smoking or alcohol consumption in pregnancy for either the Pakistani or White British women. If reporting bias, which might occur because of the stigma associated with these behaviours in pregnancy, is similar in each ethnic group it should not bias the comparisons that are the main focus of this paper. Many of the researchers who collected interview data were of Pakistani origin and it is possible that this may have resulted in greater under-reporting in the Pakistani origin women. However, the prevalence of these behaviours in this study is similar to those in other studies of Pakistani women[7].

In summary, we have found some evidence that the difference in some of these characteristics between Pakistani and White British women may be changing in response to migration to the UK, in that differences were seen most often in those where the woman or her partner were UK born. Several of these differences would be beneficial to health and wellbeing. For example, Pakistani women born in the UK were more likely than White British women to be educated beyond age 16. UK born Pakistani women were also more similar to

For peer review only - http://bmjopen.bmj.com/site/about/guidelines.xhtml

White British women in terms of employment and there was no evidence that being UK born increased their risk of GDM or glucose intolerance. On the other hand, whilst overall prevalence of smoking in Pakistani women in all groups was very small, the difference between them and White British women was least when they were UK born. We have also identified differences that vary according to the woman's partner's place of birth, for example BMI is lower among Pakistani women with a UK born partner. Further work is needed that continues to track these important ethnic differences over future generations to support the delivery of appropriate antenatal care.

Words: Abstract:300 ; Main text: 4800

Acknowledgements

Born in Bradford is only possible because of the enthusiasm and commitment of the children and parents in Born in Bradford. We are grateful to all participants, health professionals and researchers who have made Born in Bradford happen.

Funding

J West was funded by a UK Medical Research Council (MRC) Special Training Fellowship in Health of the Public and Health Services Research (MRC GO601712). DA Lawlor works in a unit that receives UK MRC funding (MC_UU_12013/5). BiB receives core infrastructure funding from the Wellcome Trust (WT101597MA).

Competing interests

All authors declare no competing interests

Author contributions

J West, DA Lawlor and J Wright conceived the study idea, obtained funds, developed the statistical analysis plan, were involved in managing the data collection and wrote the initial drafts of the paper; J West undertook the main analysis with input from L Fairley and supervision from DA Lawlor and J Wright. J West acts as guarantor.

Declaration of transparency

J West affirms that the manuscript is an honest, accurate, and transparent account of the study being reported; that no important aspects of the study have been omitted; and that any discrepancies from the study as planned (and, if relevant, registered) have been explained.

For peer review only

This is an Open Access article distributed in accordance with the Creative Commons Attribution Non Commercial (CC BY-NC 3.0) license, which permits others to distribute, remix, adapt, build upon this work non-commercially, and license their derivative works on different terms, provided the original work is properly cited and the use is non-commercial. See:http://creativecommons.org/licenses/by-nc/3.0/.

Data sharing

Scientists are encouraged and able to use BiB data. Data requests are made to the BiB executive using the form available from the study website www.borninbradford.nhs.uk (please click on "Science and Research" to access the form). Guidance for researchers and collaborators, the study protocol and the data collection schedule are all available via the website. All requests are carefully considered and accepted where possible.

Figure 1 Adjusted mean differences in BMI for Pakistani women relative to White British women

*Model 2: Adjusted for maternal age; parity; employment; post-16 education; receipt of means tested benefits; housing tenure

Figure 2 Adjusted mean differences in fasting insulin for Pakistani women relative to White British women

**Model 3: Adjusted for maternal age; parity; employment; post-16 education; receipt of means tested benefits; housing tenure; early pregnancy BMI; smoking in pregnancy

References

1. Saving Mothers' Lives: Reviewing maternal deaths to make motherhood safer: 2006-2008. *BJOG* 2011 Mar 1;118 Suppl 1:1–203

2. Office for National Statistics (ONS). Gestation-specific infant mortality in England and Wales, 2010. London, ONS 2012. At http://www.ons.gov.uk/ons/dcp171778_282579.pdf accessed on 18/03/2014.

3. Bhala N, Zaman MS. Preventing premature mortality in chronic diseases for South Asians in the UK and beyond. *J R Soc Med* 2009;102(11): 459–463.

4. Nazroo, J. Ethnicity, class and health. London, Policy Studies Institute, 2001.

5. Williams ED, Stamatakis E, Chandola T et al. Assessment of physical activity levels in South Asians in the UK: findings from the Health Survey for England. *JECH* 2011;65:517-21.

6. Patel JV, Vyas A, Cruickshank JK et al. Impact of migration on coronary heart disease risk factors: comparison of Gujaratis in Britain and their contemporaries in villages of origin in India. *Atherosclerosis* 2006;185:297-306.

7. Hawkins SS, Lamb K, Cole TJ et al. Influence of moving to the UK on maternal health behaviours: prospective cohort study. *BMJ* 2008; 336:1052.

For peer review only - http://bmjopen.bmj.com/site/about/guidelines.xhtml

8. Sproston, K; Mindell, JE. Health Survey for England 2004. The health of minority ethnic groups. HSE 2006. Information Centre, London.
9. Barnett AH, Dixon AN, Bellary S, et al. Type 2 diabetes and cardiovascular risk in the UK south Asian community. *Diabetologia* 2006;49(10):2234-46.
10. Mather HM, Keen H. The Southall Diabetes survey: prevalence of known diabetes in Asians and Europeans. *BMJ* 1985;291:1081-4.

11. Gholap N, Davies M, Patel K et al. Type 2 diabetes and cardiovascular disease in South Asians. *Prim Care Diabetes* 2011;5(1):45-56. Epub 2010/09/28.

12. Dornhurst A, Pateson CM, Nicholls JS et al. High prevalence of gestational diabetes mellitus in women from ethnic minority groups. *Diabet Med* 1992; Nov;9(9):820-5

13. Makgoba M, Savvidou MD, Steer PJ. An analysis of the interrelationship between maternal age, body mass index and racial origin in the development of gestational diabetes mellitus. *BJOG* 2012; Feb;119(3):276-82.

14. Margetts BM, Mohd Yusof S, Al Dallal Z et al. Persistence of lower birth weight in second generation South Asian babies born in the United Kingdom. *J Epidemiol Community Health* 2002 Sep;56(9):684-7.

15. Leon DA, Moser K. Low birth weight persists in South Asian babies born in England & Wales regardless of maternal country of birth. Slow pace of acculteration, physiological constraint or both? Analysis of routine data. *J Epidemiol Community Health* 2010; Published online 30 Nov 2010. doi:10.1136/jech2010.112516

16. Kelly Y, Panico L, Bartlet M et al. Why does birthweight vary among ethnic groups in the UK? Findings from the Millennium Cohort Study. *J Public Health (Oxf)* 2008;31(1):131-137.

17. West J; Lawlor DA; Fairley L et al. UK born Pakistani origin infants are relatively more adipose than White British infants: findings from 8704 mother-offspring pairs in the Born in Bradford prospective birth cohort. *J Epidemiol Community Health* 2013; doi:10.1136/jech-2012-20189.1

18. WrightJ, Small N, Raynor P et al. Cohort profile: the Born in Bradford multi-ethnic family cohort study. *Int J Epidemiol* 2013; Aug;42(4):978-91.

19. Office for National Statistics (ONS). Ethnic group statistics: a guide for the collection and classification of ethnicity data. London: Stationary Office; 2003.

20. World Health Organisation. Definition, diagnosis and classification of diabetes mellitus and its complications. Report of a WHO consultation. Part 1: diagnosis and classification of diabetes mellitus. WHO 1999 at http://whqlibdoc.who.int/hq/1999/who_ncd_ncs_99.2.pdf accessed on 18/03/2014.

21. Berthaud R. Family formation in multicultural Britain: diversity and change. In Ethnicity, social mobility and public policy, Loury GC, Modood T, Teles SM (Eds).Cambridge: Cambridge University Press; 2005.

22. Galobardes B, Shaw M, Lawlor DA et al. Indicators of socioeconomic position (Part 1). *J Epidemiol Community Health* 2006; 60(1):7-12.

For peer review only - http://bmjopen.bmj.com/site/about/guidelines.xhtml

23. Bradby H, Williams R. Is religion or culture the key feature in changes in substance use after leaving school? Young Punjabis and a comparison group in Glasgow. *Ethn and Health* 2006;11(3):307-324.

24. Hurcombe R, Bayley M, Goodman A. Ethnicity and alcohol. A review of the UK literature. Joseph Rowntree Foundation 2010.

25. Beishon S, Modood T, Virdee S. Ethnic minority families. London : Policy Studies Institute, 1998.

26. McKeigue PM, Pierpoint T, Ferrie JE et al. Relationship of glucose intolerance and hyperinsulinaemia to body fat pattern in South Asians and Europeans. *Diabetologia* 1992;35(8):785-91.

27. Ebrahim S, Kinra S, Bowen L et al. The effect of rural-to-urban migration on obesity and diabetes in India: a cross-sectional study. *PLoS Med* 2010 Apr 27;7(4).

28. Landman J, Cruickshank JK. A review of ethnicity, health and nutrition-related diseases in relation to migration in the United Kingdom. *Public Health Nutr* 2001;4 (2b):647-657.

29. Freathy RM, Kazeem GR, Morris RW et al. Genetic variation at *CHRNA5-CHRNA3-CHRNB4* interacts with smoking status to influence BMI. *Int J Epidemiol* 2011;40:1617-1628.

For peer review only - http://bmjopen.bmj.com/site/about/guidelines.xhtml

Table 1 Characteristics of women and infants (N=9450) by ethnic and generation group

	**White British (UK & Ireland)**	**All Pakistani births**	**Pakistani sub-groups defined by place of birth of parents**				
			**Pakistani: Woman & partner UK born†**	**Pakistani: Woman UK born, partner SA born†**	**Pakistani: Partner UK born, woman SA born†**	**Pakistani: Woman & partner SA born†**	**Pakistani: Other**
**Number**	3503	3656	383	992	876	1060	345
**Gestation at delivery (weeks)** Mean (sd)	39.0 (1.9)	39.0 (1.8)	39.0 (1.8)	38.9 (1.9)	39.0 (1.9)	39.1 (1.7)	39.1 (1.6)
**Births before 37 weeks N (%)**	209 (6.0)	204 (5.6)	22 (5.7)	63 (6.4)	50 (5.7)	52 (4.9)	17 (4.9)
**Mean birth weight in gm (sd)**	3346 (568)	3124(540)	3114 (538)	3100 (549)	3101 (537)	3160 (547)	3158 (497)
**Sex N (%)**							
**Male**	1808(52)	1851(51)	200(52)	504(51)	420(48)	535(51)	192(56)
**Female**	1695(48)	1805(49)	183(48)	488(49)	456(52)	525(49)	153(44)
**Maternal age** Mean (sd)	27 (6)	28 (5)	28 (5)	28 (5)	27 (5)	30 (5)	26 (5)
**Maternal height (m) Mean (sd)**	1.64 (0.06)	1.60 (0.06)	1.61 (0.05)	1.60 (0.06)	1.59 (0.05)	1.59 (0.05)	1.61 (0.06)
**Parity N (%)**							
0	1688 (48)	1157 (32)	155 (40)	331 (33)	253 (29)	254 (24)	164 (47)
1	1122 (32)	986 (26)	105 (27)	261 (26)	253 (29)	265 (25)	102 (30)
2	454 (13)	754 (21)	76 (20)	194 (20)	199 (23)	233 (22)	52 (15)
3	139 (4)	462 (13	34 (9)	125 (13)	111 (12)	178 (17)	14 (4)
4 or more	100 (3)	297 (8)	13 (4)	81 (8)	60 (7)	130 (12)	13 (4)
**Married N (%)**	1149 (33)	3571 (98)	364 (95)	974 (98)	862 (98)	1051 (99)	320 (93)
**Living with a partner N (%)**	2518 (72)	4702 (93)	352 (92)	898 (91)	852 (97)	1001 (95)	303 (88)
**Consumed alcohol during pregnancy N (%)**	266 (8)	0 (0)	0 (0)	0 (0)	0 (0)	0 (0)	0 (0)
**Total number of household members** Mean (sd)	3 (1)	5 (3)	5 (3)	5 (2)	6 (3)	5 (2)	5 (3)

†All four parents of the woman & her partner South Asian (SA) born

For peer review only - http://bmjopen.bmj.com/site/about/guidelines.xhtml

Table 2 Unadjusted and adjusted* odds ratios (95% CI) for socioeconomic characteristics for ethnic and generation groups

	**White British N=3503**	**All Pakistani births N=3656**	**Pakistani sub-groups defined by place of birth of parents**				
			**Pakistani: Woman & partner UK born[†] N=383**	**Pakistani: Woman UK born, partner SA born[†] N=992**	**Pakistani: Partner UK born, woman SA born[†] N=876**	**Pakistani: Woman &partner SA born[†] N=1060**	**Pakistani: Other N=345**
**In employment**							
Number (%)	**2272 (65)**	**881 (24)**	175 (46)	388 (39)	81 (9)	132 (12)	105 (30)
Unadjusted OR	**1**	**0.17 (0.16, 0.19)**	0.46 (0.37, 0.56)	0.35 (0.30, 0.40)	0.06 0.04, 0.07)	0.08 (0.06, 0.09)	0.24 (0.19, 0.30)
Adjusted OR*	**1**	**0.17 (0.15, 0.19)**	0.40 (0.32, 0.51)	0.38 (0.32, 0.44)	0.06 (0.04, 0.07)	0.06 (0.05, 0.08)	0.25 (0.19, 0.32)
**Educated post 16**							
Number (%)	**1601 (46)**	**1578 (43)**	235 (1)	473 (48)	306 (35)	401 (38)	163 (47)
Unadjusted OR	1	**0.90 (0.82, 0.99)**	1.89 (1.52, 2.34)	1.08 (0.94, 1.25)	0.64 (0.55, 0.74)	0.72 (0.63, 0.83)	1.06 (0.85, 1.33)
Adjusted OR*	1	**1.15 (1.04, 1.27)**	2.14 (1.70, 2.68)	1.37 (1.18, 1.59)	0.86 (0.73, 1.02)	0.88 (0.75, 1.03)	1.39 (1.11, 1.76)
**In receipt of means tested benefits****							
Number (%)	**1334 (38)**	**1742 (48)**	163 (43)	534 (54)	387 (44)	523 (49)	135 (39)
Unadjusted OR	**1**	**1.48 (1.35, 1.63)**	1.20 (0.97, 1.49)	1.90 (1.64, 2.19)	1.29 (1.11, 1.49)	1.58 (1.38, 1.89)	1.05 (0.83, 1.31)
Adjusted OR*	**1**	**0.97 (0.87, 1.09)**	1.02 (0.79, 1.30)	1.42 (1.20, 1.67)	0.71 (0.60, 0.84)	0.91 (0.78, 1.08)	0.84 (0.65, 1.09)
**Housing tenure: owns/part-owns (mortgage)**							
Number (%)	**1875 (54)**	**2600 (71)**	283 (74)	730 (74)	669 (76)	698 (66)	220 (64)
Unadjusted OR	**1**	**2.14 (1.94, 2.36)**	2.46 (1.94, 3.12)	2.42 (2.07, 2.83)	2.81 (2.37, 3.32)	1.67 (1.45, 1.93)	1.53 (1.21, 1.92)
Adjusted OR*	**1**	**2.30 (2.07, 2.56)**	2.49 (1.95, 3.18)	2.60 (2.20, 3.06)	3.35 (2.80, 3.99)	1.55 (1.32, 1.80)	2.02 (1.60, 2.57)

[†]All four parents of the woman & her partner South Asian (SA) born

*Adjusted for maternal age; parity

****** Any of: Income Support; Job Seekers Allowance; Working Tax Credit; Housing Benefits

For peer review only - http://bmjopen.bmj.com/site/about/guidelines.xhtml

Table 3 Unadjusted and adjusted* mean difference / odds ratios (95% CI) for lifestyle characteristics for ethnic and generation groups

	**White British N=3503**	**All Pakistani births N=3656**	**Pakistani sub-groups defined by place of birth of parents**				
			**Pakistani: Woman & partner UK born† N=383**	**Pakistani: Woman UK born, partner SA born† N=992**	**Pakistani: Partner UK born, woman SA born† N=876**	**Pakistani: Woman & partner SA born† N=1060**	**Pakistani: Other N=345**
**BMI at start of pregnancy** Mean (sd)	**26.8 (5.9)**	**25.7 (5.4)**	24.3 (4.6)	26.7 (5.7)	24.4 (4.7)	26.4 (5.6)	25.4 (5.3)
Unadjusted mean difference	**0**	**-1.15 (-1.41, -0.88)**	-2.53 (-3.13, -1.94)	-0.15 (-0.55, 0.25)	-2.44 (-2.86, -2.02)	-0.43 (-0.82, -0.04)	-1.40 (-2.02, -0.77)
Adjusted mean difference: Model 1*	**0**	**-1.75 (-2.01, -1.49)**	-2.84 (-3.41, -2.26)	-0.73 (-1.12, -0.34)	-2.95 (-3.36, -2.54)	-1.49 (-1.88, -1.10)	-1.22 (-1.83, -0.62)
Adjusted mean difference: Model 2**	**0**	**-1.12 (-1.43, -0.81)**	-2.32 (-2.92, -1.72)	-0.35 (-0.76, 0.07)	-2.22 (-2.69, -1.75)	-0.99 (-1.43, -0.57)	-0.77 (-1.39, -0.15)
**Smoked during pregnancy** Number (%)	**1183 (34)**	123 (3)	25 (7)	47 (5)	7 (0.8)	18 (2)	26 (8)
Unadjusted OR	**1**	**0.07 (0.06, 0.08)**	0.14 (0.09, 0.21)	0.09 (0.07, 0.13)	0.02 (0.01, 0.03)	0.03 (0.02, 0.05)	0.16 (0.11, 0.24)
Adjusted OR: Model 1*	**1**	**0.06 (0.05, 0.07)**	0.13 (0.09, 0.20)	0.09 (0.06, 0.12)	0.01 (0.01, 0.03)	0.03 (0.02, 0.05)	0.12 (0.08, 0.19)
Adjusted OR: Model 2**	**1**	**0.06 (0.05, 0.08)**	0.15 (0.09, 0.23)	0.09 (0.07, 0.13)	0.01 (0.01, 0.03)	0.03 (0.02, 0.05)	0.13 (0.08, 0.19)

†All four parents of the woman & her partner South Asian (SA) born

*Adjusted for maternal age; parity

**Adjusted for maternal age; parity; employment status; level of education, receipt of means tested benefits; housing tenure

For peer review only - http://bmjopen.bmj.com/site/about/guidelines.xhtml

Table 4 Unadjusted and adjusted* mean difference / odds ratios (95% CI) for health related pregnancy characteristics for ethnic and generation groups

	White British N=3503	All Pakistani births N=3656	Pakistani sub-groups defined by place of birth of parents				
			Pakistani: Woman & partner UK born† N=383	Pakistani: Woman UK born, partner SA born† N=992	Pakistani: Partner UK born, woman SA born† N=876	Pakistani: Woman & partner SA born† N=1060	Pakistani: Other N=345
**Hypertensive disorders of pregnancy**							
Number (%)	**239 (7)**	**188 (5)**	16 (4)	51 (5)	43 (5)	66 (6)	12 (3)
Unadjusted OR	**1**	**0.74 (0.61, 0.90)**	0.59 (0.35, 0.99)	0.74 (0.54, 1.01)	0.70 (0.51, 0.98)	0.91 (0.68, 1.20)	0.49 (0.27, 0.89)
Adjusted OR: Model 1*	**1**	**0.82 (0.67, 1.01)**	0.62 (0.37, 1.04)	0.82 (0.59, 1.12)	0.85 (0.61, 1.19)	0.99 (0.74, 1.33)	0.56 (0.31, 1.01)
Adjusted OR: Model 2**	**1**	**0.82 (0.64, 1.04)**	0.62 (0.36, 1.06)	0.81 (0.58, 1.13)	0.87 (0.59, 1.29)	1.01 (0.73, 1.40)	0.56 (0.31, 1.03)
Adjusted OR: Model 3***	**1**	**0.87 (0.67, 1.13)**	0.78 (0.45, 1.35)	0.80 (0.56, 1.14)	1.06 (0.70, 1.61)	1.06 (0.75, 1.49)	0.57 (0.31, 1.06)
**Gestational diabetes**							
Number (%)	**172 (5)**	406 (11)	30 (8)	96 (10)	92 (11)	159 (15)	29 (8)
Unadjusted OR	**1**	2.42 (2.01, 2.91)	1.65 (1.09, 2.46)	2.07 (1.59, 2.69)	2.27 (1.74, 2.96)	3.42 (2.72, 4.29)	1.78 (1.18, 2.68)
Adjusted OR: Model 1*	**1**	2.41 (1.98, 2.94)	1.66 (1.10, 2.49)	2.07 (1.58, 2.71)	2.54 (1.92, 3.35)	3.01 (2.36, 3.83)	2.24 (1.47, 3.41)
Adjusted OR: Model 2**	**1**	2.28 (1.82, 2.86)	1.66 (1.09, 2.53)	1.98 (1.49, 2.64)	2.47 (1.79, 3.39)	2.89 (2.20, 3.82)	2.21 (1.44, 3.40)
Adjusted OR: Model 3***	**1**	2.38 (1.86, 3.03)	1.89 (1.23, 2.92)	1.98 (1.46, 2.67)	2.82 (2.01, 3.97)	3.04 (2.27, 4.08)	2.29 (1.47, 3.56)

For peer review only - http://bmjopen.bmj.com/site/about/guidelines.xhtml

**Fasting glucose** Mean (sd)	**4.41 (0.41)**	**4.62 (0.62)**	4.54 (0.47)	4.58 (0.64)	4.54 (0.48)	4.73 (0.76)	4.60 (0.53)
Unadjusted mean difference	**0**	**0.20 (0.18, 0.23)**	0.13 (0.08, 0.19)	0.17 (0.14, 0.21)	0.13 (0.09, 0.17)	0.32 (0.29, 0.36)	0.19 (0.13, 0.25)
Adjusted mean difference: Model 1*	**0**	**0.18 (0.16, 0.21)**	0.12 (0.06, 0.17)	0.15 (0.11, 0.19)	0.12 (0.09, 0.16)	0.27 (0.24, 0.31)	0.22 (0.16, 0.27)
Adjusted mean difference: Model 2**	**0**	**0.18 (0.15, 0.21)**	0.12 (0.06, 0.18)	0.15 (0.11, 0.19)	0.12 (0.07, 0.16)	0.27 (0.23, 0.31)	0.22 (0.16, 0.27)
Adjusted mean difference: Model 3***	**0**	**0.20 (0.17, 0.24)**	0.17 (0.12, 0.23)	0.16 (0.12, 0.19)	0.17 (0.12, 0.21)	0.29 (0.25, 0.33)	0.23 (0.17, 0.29)
**Postload glucose** Mean (sd)	**5.47 (1.30)**	**5.89 (1.68)**	5.59 (1.35)	5.81 (1.58)	5.82 (1.50)	6.12 (2.02)	5.73 (1.45)
Unadjusted mean difference	**0**	**0.42 (0.35, 0.49)**	0.12 (-0.04, 0.28)	0.34 (0.23, 0.45)	0.35 (0.24, 0.46)	0.72 (0.62, 0.83)	0.26 (0.09, 0.42)
Adjusted mean difference: Model 1*	**0**	**0.37 (0.29, 0.44)**	0.08 (-0.07, 0.24)	0.29 (0.18, 0.39)	0.35 (0.24, 0.46)	0.58 (0.48, 0.69)	0.35 (0.19, 0.51)
Adjusted mean difference: Model 2**	**0**	**0.35 (0.27, 0.43)**	0.10 (-0.06, 0.26)	0.28 (0.17, 0.39)	0.33 (0.20, 0.46)	0.56 (0.44, 0.68)	0.34 (0.18, 0.51)
Adjusted mean difference: Model 3***	**0**	**0.37 (0.28, 0.45)**	0.18 (0.02, 0.34)	0.27 (0.16, 0.38)	0.39 (0.26, 0.52)	0.57 (0.45, 0.69)	0.35 (0.18, 0.52)
**Fasting insulin** Mean (sd)	**81.40 (46.72)**	**100.28 (62.76)**	92.66 (65.59)	100.76 (56.46)	91.75 (49.04)	106.11 (68.84)	111.09 (81.89)
Unadjusted mean difference	**0**	**18.88 (16.31, 21.45)**	11.26 (5.42, 17.09)	19.36 (15.46, 23.26)	10.36 (6.26, 14.45)	24.71 (20.91, 28.51)	29.69 (23.58, 35.81)
Adjusted mean difference: Model 1*	**0**	**18.08 (15.42, 20.74)**	10.98 (5.13, 16.82)	18.59 (14.64, 22.54)	9.67 (5.51, 13.83)	23.36 (19.43, 27.30)	29.69 (23.55, 35.82)
Adjusted mean difference: Model 2**	**0**	**21.29 (18.13, 24.45)**	14.01 (7.95, 20.08)	20.62 (16.40, 24.83)	13.53 (8.73, 18.34)	25.24 (20.89, 29.59)	32.01 (25.72, 38.31)
Adjusted mean difference: Model 3***	**0**	**25.71 (22.73, 28.69)**	24.44 (19.03, 29.86)	21.29 (17.47, 25.13)	23.27 (18.86, 27.68)	29.03 (25.04, 33.02)	34.79 (29.18, 40.39)

†All four parents of the woman & her partner South Asian (SA) born
*Adjusted for maternal age; parity
**Adjusted for maternal age; parity; employment status; level of education, receipt of means tested benefits; housing tenure
*** Adjusted for maternal age; parity; employment status; level of education, receipt of means tested benefits; housing tenure; early pregnancy BMI; smoking in pregnancy

For peer review only - http://bmjopen.bmj.com/site/about/guidelines.xhtml

Differences in socioeconomic position, lifestyles and health related pregnancy characteristics between Pakistani and White British women: the influence of the woman's, her partner's and their parents' place of birth.

West J *Specialty Registrar in Public Health*[1], **Lawlor DA** *professor of epidemiology* [2,3], **Fairley L** *statistician*[1], **Wright J** *professor of clinical epidemiology*[1]

[1] Bradford Institute for Health Research, Bradford Royal Infirmary, Bradford, BD9 6RJ, UK

[2] MRC Integrative Epidemiology Unit at the University of Bristol, UK

[3]School of Social and Community Medicine, University of Bristol, UK

Correspondence to jane.west@bthft.nhs.uk

For peer review only

ABSTRACT

Objective

To examine differences between Pakistani and White British women in relation to socioeconomic position, lifestyle characteristics and health related pregnancy characteristics, and to determine whether these differences vary depending on the woman's, her partner's and both of their parents' place of birth.

Design

Prospective cohort study.

Setting

Bradford, UK

Participants

3656 Pakistani and 3503 White British women recruited to the Born in Bradford study.

Main outcome measures

Socioeconomic position (employment status; level of education; receipt of benefits; housing tenure), lifestyle characteristics (BMI at the start of pregnancy; smoking during pregnancy) and health related pregnancy characteristics (hypertensive disorders of pregnancy; gestational diabetes; fasting glucose, postload glucose and fasting insulin at ~27 weeks gestation).

Results

Pakistani women were less likely to be employed (OR 0.17 95% CI 0.15, 0.19), the difference being markedly less for UK born women. UK born Pakistani women were more likely, and South Asian born less likely, to be educated post 16 than White British women. Smoking was uncommon among Pakistani women, though the difference comparing UK born Pakistani women to White British women was less than for other groups. BMI was lower among Pakistani compared to White British women (adjusted mean difference -1.12 95% CI -1.43, -0.81) the difference greatest when partners were UK born irrespective of the woman's place of birth. Pakistani women had higher fasting and postload glucose (mean difference 0.20 mmol/l 95% CI 0.17, 0.24; 0.37 95% CI 0.28, 0.45), higher fasting insulin and were more likely to have gestational diabetes.

For peer review only - http://bmjopen.bmj.com/site/about/guidelines.xhtml

Conclusions

Our results suggest that some socioeconomic, lifestyle and pregnancy characteristics could be beginning to change in response to migration to the UK, with generally beneficial i.e. improving education and employment prospects, lower BMI and no evidence that being UK born has further increased the risk of GDM, but some negative i.e. slight increases in smoking.

Strengths & limitations of this study

~~We explored differences in socioeconomic, lifestyle and pregnancy characteristics between UK Pakistani and White British women.~~

~~We found that some differences are reduced and some are enhanced in UK born Pakistani women suggesting both positive and negative changes in response to migration.~~

The strengths of this study include a large sample size, range of outcomes including oral glucose tolerance test data and detailed ancestry information.

We have for the first time, been able to examine ethnic differences in socioeconomic, lifestyle and pregnancy characteristics using information on the place of birth of women and their partners. We had also set out to explore differences based on all four grandparents but once we began analysing data it was apparent that for the majority of Pakistani women and their partners, all four of their parents were South Asian born. This limited our ability to explore differences across two generations, but highlights the persistence of strong family links in this community that have lived in Bradford for over 6 decades.

~~Place of birth of both women and their partners may be important to lifestyle choices in this population.~~

A potential limitation is that our results may not be generalizable to other South Asian populations and further work will be important to track these differences over future generations of UK South Asian migrants.

For peer review only - http://bmjopen.bmj.com/site/about/guidelines.xhtml

INTRODUCTION

Migration of South Asian populations to high income countries is generally thought to offer socioeconomic advantages in the form of improved education and employment opportunities, better housing and access to health care. However, improvements in environmental circumstances do not necessarily translate into improvements in health outcomes. Indeed, South Asian migrant populations to the UK experience an increased risk of maternal[1] and infant mortality[2] and some chronic diseases[3] compared with the UK population as a whole. This may reflect the effects of previous disadvantage associated with the country of origin which could persist over several generations, or could be a consequence of poor socioeconomic status within the host country, UK South Asian communities are on average very poor[4]. That is, it could be that in comparison to those who do not migrate, there are improved health outcomes, but these remain poorer in comparison to the indigenous population. A further explanation is that the adoption of the unhealthy and sedentary lifestyles associated with acculturation or Westernisation, often characterised by low levels of physical activity[5], consumption of high calorie energy rich diets[6] and cigarette smoking[7,8], counteracts any potential health advantage of living in a higher income country. This may vary across different migrant communities but where this is the case, adoption of such lifestyles may be particularly harmful to South Asian individuals who for a given body mass index (BMI), have greater total and central adiposity and are known to be at greater risk of type 2 diabetes and cardiovascular disease than European adults[9-11].

Ethnic differences in socioeconomic position and lifestyle that might impact health during pregnancy could contribute to some of the known ethnic differences in pregnancy complications and perinatal outcomes. For example, they could contribute to the established greater risk of gestational diabetes (GDM)[12,13] and small for gestational age (SGA)[14-16] in South Asian compared to White British women. They could also drive ethnic differences in future generations either through intrauterine effects of maternal behaviours on these or as a result of the adoption of parental lifestyles by offspring and a lack of social migration. Previous studies have reported ethnic differences in socioeconomic and lifestyle characteristics between South Asian and White British women during pregnancy. Findings from the Millennium Cohort Study suggest South Asian women, in particular those originating from Pakistan and Bangladesh, are less likely to have formal educational qualifications, more likely to belong to lower socioeconomic groups and more likely to have never worked or be long term unemployed[7,16]. Marked differences in smoking and alcohol consumption between South Asian and White British women have also been reported[7,17]. Whilst outside pregnancy BMI is reportedly higher among South Asian women compared to White British women[18], we have previously reported that BMI is lower among Pakistani origin

For peer review only - http://bmjopen.bmj.com/site/about/guidelines.xhtml

pregnant women in the Born in Bradford (BiB) cohort[17]. Much less is known about maternal blood glucose and insulin in particular whether there are differences in these outcomes across generations of UK South Asian migrants. To our knowledge, no previous studies have examined ethnic differences in all these characteristics (socioeconomic, lifestyle, pregnancy) collectively which is important to identify areas where South Asian women may have better outcomes and those where European women may have better outcomes. This knowledge could support the delivery of appropriate antenatal care aimed at maximising maternal and child health in both White British and South Asian groups.

Furthermore, previous studies have not explored whether any identified ethnic differences during pregnancy are consistent when the mother's, her partner's and both of their parents' country of origin are taken into account. In a previous study, using data from the Born in Bradford cohort, which is used in this paper, we showed that birthweight was lower, but that birth fatness (assessed using skinfold thickness and cord blood leptin) was greater in Pakistani compared to White British infants[17]. We further showed that these differences did not differ by whether both the mother and her partner and all four of their parents were born in the UK, all born in South Asia or there was a mixed pattern between these two extremes[17]. Here, we extend that work to look at a range of socioeconomic position, lifestyle and pregnancy related outcomes, in order to understand whether in the context of place of birth of women and her closest family relatives, there are some ethnic differences that are reduced or some that are enhanced, and if so whether these would be beneficial or detrimental to health.

The aim of this study is to examine differences between Pakistani women and White British women in relation to socioeconomic position (employment status; level of education; receipt of means tested benefits; housing tenure), lifestyle characteristics (BMI at the start of pregnancy; smoking during pregnancy) and health related pregnancy characteristics (hypertensive disorders of pregnancy (HDP); gestational diabetes; fasting glucose, postload glucose and fasting insulin at ~27 weeks gestation), and to determine whether these differences vary depending upon the woman's, her partner's and both of their parents' place of birth.

METHODS

Participants

The Born in Bradford (BiB) study is a largely bi-ethnic prospective birth cohort study that recruited women during pregnancy and has followed them, their infants and their partners into the child's infancy. To be eligible for the study women had to attend booking clinic

For peer review only - http://bmjopen.bmj.com/site/about/guidelines.xhtml

between March 2007 and December 2010 and be booked to give birth in the city of Bradford. Full details of the study methodology have been previously reported[18]. Women were recruited at their oral glucose tolerance test (OGTT) appointment; all women booked for delivery in Bradford are offered a 75g OGTT (comprising fasting and 2 hour postload samples) at around 26 – 28 weeks gestation. Women who attended this appointment and agreed to take part in the study consented to the use of their obstetric medical records, had their height and weight recorded and completed an interviewer administered questionnaire. The questionnaire included questions relating to ethnicity, social and economic circumstances, smoking, alcohol, diet, education and employment and collected place of birth information for both parents and all four grandparents. Interviews were conducted in a range of South Asian languages (including Mirpuri, Bengali, Punjabi). Mirpuri is the most commonly spoken Asian language in Bradford but has no written script therefore questionnaires were transliterated, that is translated verbally to Mirpuri and then written phonetically, precisely as spoken to ensure that all interpreters translated it in the same way. Details of the language used to conduct the questionnaire were recorded. Ethics approval for the study was provided by Bradford Local Research Ethics Committee (ref 06/Q1202/48). Data were available for 11,113 women recruited to the BiB cohort. We excluded stillbirths (n=64) and infants born to parents of ethnic origin other than White British or Pakistani (n=1598~~1605~~). Of the remaining 9451 participants 7159 had complete data for all variables included in all models thus 3656 Pakistani and 3503 White British women are included in these analyses. Women with existing diabetes (0.5% of the BiB cohort) are not invited to attend for the glucose tolerance test as they are treated from the start of their pregnancy by an endocrine physician. This means that these women were not recruited at the same time as other participants and do not have some data, including parental place of birth, therefore these women are not included in these complete case analyses.

Woman's family member's place of birth

Ethnicity was self-reported at interview, with participants given response options based on UK Office of National Statistics guidance[19]. Women completed a detailed ancestry interview, which included details of the place of birth of themselves, their partner and all four parents of themselves and their partner. Family place of birth groups of the Pakistani infants were derived from these data as previously reported[17]. In the previous report, since our outcome of interest was infant birth size the groups were defined in terms of 'parents' and 'grandparents'. As our outcomes here are in pregnant women we have described them in relation to her, but the groups are essentially the same as the previous paper. Our aim in that previous paper, as here, was to examine differences across all possible groups based on place of birth of the woman, her partner and all four parents. Thus, we began by

For peer review only - http://bmjopen.bmj.com/site/about/guidelines.xhtml

determining numbers in all 64 possible combinations of these six family members. Having done that it was apparent that for almost all women, the four parents of the woman and her partner were South Asian born meaning that the analyses were based primarily on the woman's and her partner's place of birth. Overall, 90% of women fell into one of four main categories:

1. Woman and her partner UK born and all four of their parents South Asian born
2. Woman UK born, partner and all four of their parents South Asian born
3. Partner UK born, woman and all four parents South Asian born
4. Woman, her partner and four parents all South Asian born

The remaining 11% (n=345), including those with one or more of the woman's or her partner's parents being UK born or where their parents' place of birth was unknown, was combined to form one 'other' group.

Outcome measures

Socioeconomic

Information on socioeconomic indicators (employment, education, receipt of benefits, housing tenure) was obtained from the interview with the woman at recruitment. We equivalised the mother's highest educational qualifications (based on the qualification received and the country obtained) into one of several categories using UK NARIC (http://www.ecctis.co.uk/naric/default.aspx): *<5 GCSE equivalent, ≥5 GCSE equivalent, 'A' level equivalent, Higher than A-level equivalent, Other qualifications (e.g. City and Guilds, RSA/OCR, BTEC), Don't know, Foreign Unknown.* Don't know relates to the mother responding "don't know" during interview. Foreign Unknown relates to a qualification listed in the free text response but no level of qualification is given or the qualification listed cannot be equivalised to one of the above categories. For these analyses, women were categorised as having been educated beyond the age of 16 or not (i.e. *Higher than A-level equivalent, Other qualifications (e.g. City and Guilds, RSA/OCR, BTEC). Information* Receipt of means tested benefits was based on the mother or her household receiving any of: Income Support, Job Seekers Allowance, Working Tax Credit or Housing Benefit. Housing tenure was categorised according to whether the woman lived in a household where the home was either part-owned (i.e. mortgaged) or owned outright, or not (i.e. rented).

Lifestyle

BMI is used in these analyses as a proxy marker of lifestyle as it is an outcome that can potentially be influenced by changes or differences in lifestyle (in particular dietary choices

For peer review only - http://bmjopen.bmj.com/site/about/guidelines.xhtml

and levels of physical activity). At recruitment, women were weighed and their height measured (unshod and in light clothing) using SECA digital scales and a Leicester Height Measure respectively. Weight at first antenatal clinic assessment when women were around 12 weeks gestation (median 12 weeks, IQR 11, 14), was abstracted from the antenatal records and this weight together with height measured at recruitment, was used to calculate the woman's BMI so that this reflected early pregnancy BMI before substantial contribution from pregnancy and the growing fetus. Information on smoking was obtained at the questionnaire interview, with women categorised as having smoked cigarettes at any stage of their pregnancy or not. As none of the Pakistani origin women reported drinking alcohol, we were unable to include alcohol consumption as an outcome.

Health related pregnancy characteristics

Women were classified as hypertensive in pregnancy if they had a systolic measure ≥140 and a diastolic ≥90mmHg on 2 or more occasions after 20 weeks gestation; information on this was obtained from the antenatal records. Fasting and postload glucose and fasting insulin were obtained from the OGTT plasma samples which were assayed immediately after sampling at the biochemistry department of Bradford Royal Infirmary using the glucose oxidase method on Siemen's Advia 2400 chemistry autoanalysers. GDM was defined using the fasting and postload glucose according to WHO criteria[20] at the time these women were pregnant as either a fasting glucose ≥6.1mmol/L or a two-hour postload glucose ≥7.8mmol/L. Women with existing diabetes prior to pregnancy did not complete an OGTT and are not included in this sample.

Statistical analyses

All analyses were performed using Stata (version 12.1). We used univariable regression to examine the association of ethnicity and family place of birth group with outcomes. Logistic regression was used for binary outcomes and linear regression for continuous outcomes, with the White British group used as the reference for all analyses, i.e. we compared outcomes in 'all' Pakistani women and then each of the five family place of birth subgroups of Pakistani women to outcomes in White British women. The rationale for this is because our aim is primarily to compare all Pakistani origin women with White British women and then to compare subgroups based on place of birth with the same reference group of White British women to see if place of birth of the Pakistani women influences the extent to which they differ or not from the indigenous population. In all adjusted analyses we adjusted for maternal age and parity (Model 1). In addition to the model adjusting for maternal age and parity, for the lifestyle outcomes (early pregnancy BMI; smoking) we also adjusted for each of the indicators of socioeconomic position in order to explore the extent of any differences

For peer review only - http://bmjopen.bmj.com/site/about/guidelines.xhtml

in these lifestyles might reflect ethnic differences in socioeconomic position (Model 2). For the health related pregnancy characteristics we also adjusted for socioeconomic indicators (Model 2) and also for the lifestyle characteristics (BMI; smoking) (Model 3), to explore whether these explained any of the differences.

RESULTS

The characteristics of White British and Pakistani origin women are shown in Table 1. There was little difference between the two ethnic groups in mean gestation, premature births and infant sex. As reported in our previous paper[17], birthweight of their infant was markedly lower in Pakistani compared to White British women when all Pakistani origin women were combined and also when compared by subgroups based on place of birth. Pakistani origin women were on average slightly older, in particular when both parents were South Asian born, markedly more likely to be married and on average they lived within larger households than White British women. These differences were similar across all generation groups. Pakistani women were shorter than White British women but the difference was less when women were UK born. There were also some differences in parity across Pakistani generation groups, for example, parity was on average lowest when both parents were UK born and highest when both parents were born in South Asia.

Pakistani women as a whole were 83% less likely to be employed (adjusted OR 0.17 95% CI 0.15, 0.19) than White British women, but there were differences by family place of birth (Table 2). Those who were South Asian born were 94% less likely to be in employment but this difference reduced to 60% for Pakistani women when both they and their partner were UK born. Following adjustment for maternal age and parity, Pakistani women as a whole were more likely to be educated beyond the age of 16 than White British women (OR 1.15 95% CI 1.04, 1.27), however there were marked differences across family place of birth groups with women who were South Asian born being less likely, and those who were UK born being more likely compared to White British women, to be educated beyond 16 years. Being in receipt of means tested benefits was similar in both ethnic groups when Pakistani women were assessed as a whole (adjusted OR 0.97 95% CI 0.87, 1.09) although for Pakistani women who were UK born with a South Asian partner there were increased odds of receiving benefits., especially when they were UK born but their partner and parents were born in South Asia (adjusted OR 1.42 95% CI 1.20, 1.67). Compared to White British women, Pakistani women were considerably more likely to own or part own their home (adjusted OR 2.30 95% CI 2.07, 2.56) and this was consistent across all family place of birth groups.

For peer review only - http://bmjopen.bmj.com/site/about/guidelines.xhtml

Table 3 shows the unadjusted and adjusted (Models 1 and 2) ethnic difference in lifestyle characteristics. Pakistani women had a lower BMI than White British women (adjusted [Model 2] mean difference -1.12 95% CI -1.43, -0.81) but the difference was much greater when the woman's partner was UK born irrespective of where the woman herself was born (Figure 1). Pakistani women were around 94% less likely to smoke and this was similar across generation groups other than when both the woman and her partner were UK born in which case women were 85% less likely to have smoked during pregnancy. None of the Pakistani women reported drinking any alcohol during pregnancy (0%), whereas 8% of White British women drank during pregnancy.

In Table 4 the unadjusted and adjusted (Models 1-3) ethnic difference in pregnancy characteristics is shown. Pakistani women in general were less likely to have HDP (adjusted [Model 3] OR 0.87 95% CI 0.67, 1.13), although this result was imprecisely estimated with wide confidence intervals that included the null. This was not consistent across all family place of birth groups for example, women who were South Asian born were slightly more likely to have HPD than White British women and this was the case in all 3 adjusted models. Pakistani women were more likely to have GDM and higher fasting and postload glucose and fasting insulin than White British women and these differences were broadly similar across all 3 models of adjustment. There were some differences by family place of birth group, for example, the difference in postload glucose between Pakistani and White British women was far greater when the woman and her partner were born in South Asia than when both were UK born (adjusted mean difference [Model 3] 0.57 95% CI 0.45, 0.69 and 0.18 95% CI 0.02, 0.34 respectively and Figure 2).

DISCUSSION

We have shown number of differences in across a range of socioeconomic, lifestyle and pregnancy characteristics between Pakistani and White British origin women and that these vary depending on whether Pakistani women are born in the UK or South Asia. We have for the first time, been able to consider not only the woman's place of birth, but also determine whether these differences vary depending on the woman's, her partner's and both of their parents' place of birth; though after preliminary analyses it was clear that for the majority of women and their partners, all four of their parents were South Asian born. This provides important information about how these differences might be reduced or even enhanced with greater acculturation over generations. For example, Pakistani women as a whole, were 83% less likely to be in employment than White British women, but across generation groups this difference varied from 60% when both the woman and her partner were born in the UK, to 94% when both the woman and her partner were South Asian born. Likewise, we found

For peer review only - http://bmjopen.bmj.com/site/about/guidelines.xhtml

interesting differences in education attainment between Pakistani and White British women. Overall, Pakistani women were slightly more likely to have been educated beyond the age of 16, but this was driven by UK born Pakistani women, especially those with a UK born partner who were twice as likely as White British women to have completed education beyond age 16. By contrast, South Asian born Pakistani women, irrespective of their partner's place of birth, were less likely than White British women to have been educated beyond the age of 16. This could reflect a positive effect of migration and acculturation on social mobility which likely plays a part in the employment differences described above and is consistent with previous reports[7,21]. Whilst differences in employment and education by place of birth suggest the adoption of some British ~~behaviours and~~ lifestyle characteristics, the tendency of Pakistani women to live within larger households and to be more likely to own or part-own their own home, suggests that the traditional culture of living within extended families has been maintained across all place of birth sub-groups of Pakistani women.~~in this population~~. Living with an extended family could have considerable benefits for the mother and her offspring, such as childcare support and greater social capital, but could also result in overcrowding and potential detrimental impacts of this on health[22]. Early analyses using data from BiB suggests that living with more family members does not lead to greater family social capital (Cabieses B, unpublished data 2013). Pakistani women who were born in the UK but had a South Asian born partner, were more likely to claim benefits compared to White British women than those who were South Asian born which is surprising given that they tend to be more likely to be in employment. This might reflect a tendency for South Asian born partners to be in lower paid employment reducing total household income, or that poorer command of the English language (likely amongst those Pakistani women who were South Asian born and were less likely to claim benefits compared to White British women) is a barrier to accessing services and social support.

Greater social migration, for example coming to the UK for social reasons, has been associated with increased uptake of lifestyle characteristics of the host country such as smoking and alcohol consumption (Hawkins 2008). We report a similar trend in that UK born Pakistani origin women were ~~slightly~~ more likely to smoke than South Asian born women, but smoking was still uncommon among all Pakistani women compared to White British women and none of them reported any alcohol consumption during pregnancy. Thus, the increase in these harmful health behaviours over generations in some migrant groups, whilst showing some signs of change, appears minimal among Pakistani women.~~,~~ This~~which~~ may reflect persisting cultural or religious influences[23,24] and could be related to the fact that for the majority of women, both of their parents and their partners parents were South Asian born~~.~~ We found BMI to be slightly lower among Pakistani origin women compared to White

For peer review only - http://bmjopen.bmj.com/site/about/guidelines.xhtml

British women although there were interesting differences across family place of birth groups. The finding that the difference in BMI between Pakistani and White British women was markedly greater for Pakistani women with a UK born partner, irrespective of their own place of birth, than for women with a South Asian born partner is particularly striking. One possible explanation is that within this population, partners/husbands have a particularly dominant role[25]. Thus, the lifestyle choices of the family or household will be driven mostly by the social norms and habits of the partner. In the case of men born in the UK, these are likely to be influenced by western culture which promotes a lower BMI as both healthy and attractive. Similarly, having been brought up and educated in the UK, they may be more likely to participate in organised physical activity and also may be more receptive to UK public health campaigns.

Health related pregnancy characteristics may be the most important to the long-term health of South Asian migrants in the UK, particularly in relation to the association of these characteristics with cardiovascular disease and type 2 diabetes[26]. We report a number of differences between Pakistani women and White British women in HDP, glucose tolerance, fasting insulin and GDM. Pakistani women as a whole group were less likely to have HDP, although this was not consistent across family subgroups, but more than twice as likely to have GDM. Consistent with these higher rates of GDM, Pakistani women had higher fasting and postload glucose and higher fasting insulin than White British women. These findings are similar to those from previous studies showing South Asian women are more likely to have GDM than White European women[12,13] and considerable evidence that adult non-pregnant women and men have a higher risk of insulin resistance and type 2 diabetes[9-11,26]. We found that the increased likelihood of Pakistani women having GDM compared to White British women was greatest for South Asian born women. We also found that the mean difference in fasting and postload glucose and fasting insulin relative to White British women was substantively greater when the woman and her partner were both born in South Asia. This is somewhat surprising as evidence suggests that the increased risk of insulin resistance and type 2 diabetes in South Asian adults compared to White Europeans is largely amongst those in urban (rather than rural) areas of South Asia[27], or in those who have migrated to Western countries[9,28]. We might therefore have expected the increase to be greater amongst those who were UK born. The difference between our findings and these previous studies of non-pregnant migrants[9,26,27], might be explained by differences in the population studied, with many of these previous studies being of~~are in~~ Indian, or mixed rather than Pakistani origin. Pakistani migrants in general tend to be poorer, shorter and weigh less, and the Pakistani women in this study have lower BMI than the White British women. For religious and cultural reasons Pakistani women remain ~~are particularly~~ unlikely

For peer review only - http://bmjopen.bmj.com/site/about/guidelines.xhtml

to smoke or drink alcohol, this might influence their glucose tolerance, though smoking is related to lower BMI and so would be expected to reduce glucose tolerance[29]. It might also be that whilst insulin resistance and diabetes in the general population are enhanced in those who migrate and particularly with greater duration of migration, in pregnancy the impact of place of birth or time since migration differs. We are not aware of other studies with equivalent data to explore this further, but it would be interesting to see if this finding does replicate.

The key strengths of this study are the large sample size, range of outcomes we have been able to examine, including OGTT data, and the detailed information on place of birth. To our knowledge this is the first study to examine differences between Pakistani and White British women in relation to socioeconomic, lifestyle and pregnancy characteristics using detailed information on the place of birth of women and their partners. We had hoped to explore three generations of Pakistani migrants to Bradford, but for almost all the women in this study, their parents and the parents of their partner were born in South Asia. However, this is in itself an interesting finding and useful for meeting future health needs in the city. It might also explain some of our findings in relation to the persistence of some characteristics across family place of birth subgroups. A potential limitation of our study was the inability to include other South Asian groups in our analyses (Indian and Bangladeshi) due to small numbers within our cohort. On the one hand examining a specific South Asian population (Pakistani) reduces the problem of heterogeneity between South Asian groups but at the same time it may limit the generalisability of our results to other South Asian populations. Our analyses have not accounted for South Asians who migrate to the UK in childhood and may be resident in the UK for much of their development and education, which could potentially dilute any differences between the Pakistani place of birth groups. Within BiB information regarding the age at which an individual migrated to the UK is only available for women (not their partner or parents) therefore we were not able to account for this in our family place of birth groups. We were not able to validate self-report of smoking or alcohol consumption in pregnancy for either the Pakistani or White British women. If reporting bias, which might occur because of the stigma associated with these behaviours in pregnancy, is similar in each ethnic group it should not bias the comparisons that are the main focus of this paper. Many of the researchers who collected interview data were of Pakistani origin and it is possible that this may have resulted in greater under-reporting in the Pakistani origin women. However, the prevalence of these behaviours in this study is similar to those in other studies of Pakistani women[7].

For peer review only - http://bmjopen.bmj.com/site/about/guidelines.xhtml

In summary, we have found some evidence that the difference in some of these characteristics between Pakistani and White British women may be changing in response to migration to the UK, in that differences were seen most often in those where the woman or her partner were UK born. Several of these differences would be beneficial to health and wellbeing. For example, Pakistani women born in the UK were more likely than White British women to be educated beyond age 16. UK born Pakistani women were also more similar to White British women in terms of employment and there was no evidence that being UK born increased their risk of GDM or glucose intolerance. On the other hand, whilst overall prevalence of smoking in Pakistani women in all groups was very small, the difference between them and White British women was least when they were UK born. We have also identified differences that vary according to the woman's partner's place of birth, for example BMI is lower among Pakistani women with a UK born partner. Further work is needed that continues to track these important ethnic differences over future generations to support the delivery of appropriate antenatal care.

Words: Abstract:300 ; Main text: 4800

Acknowledgements

Born in Bradford is only possible because of the enthusiasm and commitment of the children and parents in Born in Bradford. We are grateful to all participants, health professionals and researchers who have made Born in Bradford happen.

Funding

J West was funded by a UK Medical Research Council (MRC) Special Training Fellowship in Health of the Public and Health Services Research (MRC GO601712). DA Lawlor works in a unit that receives UK MRC funding (MC_UU_12013/5). BiB receives core infrastructure funding from the Wellcome Trust (WT101597MA).

Competing interests

All authors declare no competing interests

Author contributions

J West, DA Lawlor and J Wright conceived the study idea, obtained funds, developed the statistical analysis plan, were involved in managing the data collection and wrote the initial drafts of the paper; J West undertook the main analysis with input from L Fairley and supervision from DA Lawlor and J Wright. J West acts as guarantor.

For peer review only - http://bmjopen.bmj.com/site/about/guidelines.xhtml

Declaration of transparency

J West affirms that the manuscript is an honest, accurate, and transparent account of the study being reported; that no important aspects of the study have been omitted; and that any discrepancies from the study as planned (and, if relevant, registered) have been explained.

This is an Open Access article distributed in accordance with the Creative Commons Attribution Non Commercial (CC BY-NC 3.0) license, which permits others to distribute, remix, adapt, build upon this work non-commercially, and license their derivative works on different terms, provided the original work is properly cited and the use is non-commercial. See:http://creativecommons.org/licenses/by-nc/3.0/.

Data sharing

Scientists are encouraged and able to use BiB data. Data requests are made to the BiB executive using the form available from the study website www.borninbradford.nhs.uk (please click on "Science and Research" to access the form). Guidance for researchers and collaborators, the study protocol and the data collection schedule are all available via the website. All requests are carefully considered and accepted where possible.

Figure 1 Adjusted mean differences in BMI for Pakistani women relative to White British women

*Model 2: Adjusted for maternal age; parity; employment; post-16 education; receipt of means tested benefits; housing tenure

Figure 2 Adjusted mean differences in fasting insulin for Pakistani women relative to White British women

**Model 3: Adjusted for maternal age; parity; employment; post-16 education; receipt of means tested benefits; housing tenure; early pregnancy BMI; smoking in pregnancy

References

1. Saving Mothers' Lives: Reviewing maternal deaths to make motherhood safer: 2006-2008. *BJOG* 2011 Mar 1;118 Suppl 1:1–203

2. Office for National Statistics (ONS). Gestation-specific infant mortality in England and Wales, 2010. London, ONS 2012. At http://www.ons.gov.uk/ons/dcp171778_282579.pdf accessed on 18/03/2014.

3. Bhala N, Zaman MS. Preventing premature mortality in chronic diseases for South Asians in the UK and beyond. *J R Soc Med* 2009;102(11): 459–463.

4. Nazroo, J. Ethnicity, class and health. London, Policy Studies Institute, 2001.

5. Williams ED, Stamatakis E, Chandola T et al. Assessment of physical activity levels in South Asians in the UK: findings from the Health Survey for England. *JECH* 2011;65:517-21.

For peer review only - http://bmjopen.bmj.com/site/about/guidelines.xhtml

6. Patel JV, Vyas A, Cruickshank JK et al. Impact of migration on coronary heart disease risk factors: comparison of Gujaratis in Britain and their contemporaries in villages of origin in India. *Atherosclerosis* 2006;185:297-306.

7. Hawkins SS, Lamb K, Cole TJ et al. Influence of moving to the UK on maternal health behaviours: prospective cohort study. *BMJ* 2008; 336:1052.

8. Sproston, K; Mindell, JE. Health Survey for England 2004. The health of minority ethnic groups. HSE 2006. Information Centre, London.
9. Barnett AH, Dixon AN, Bellary S, et al. Type 2 diabetes and cardiovascular risk in the UK south Asian community. *Diabetologia* 2006;49(10):2234-46.
10. Mather HM, Keen H. The Southall Diabetes survey: prevalence of known diabetes in Asians and Europeans. *BMJ* 1985;291:1081-4.

11. Gholap N, Davies M, Patel K et al. Type 2 diabetes and cardiovascular disease in South Asians. *Prim Care Diabetes* 2011;5(1):45-56. Epub 2010/09/28.

12. Dornhurst A, Pateson CM, Nicholls JS et al. High prevalence of gestational diabetes mellitus in women from ethnic minority groups. *Diabet Med* 1992; Nov;9(9):820-5

13. Makgoba M, Savvidou MD, Steer PJ. An analysis of the interrelationship between maternal age, body mass index and racial origin in the development of gestational diabetes mellitus. *BJOG* 2012; Feb;119(3):276-82.

14. Margetts BM, Mohd Yusof S, Al Dallal Z et al. Persistence of lower birth weight in second generation South Asian babies born in the United Kingdom. *J Epidemiol Community Health* 2002 Sep;56(9):684-7.

15. Leon DA, Moser K. Low birth weight persists in South Asian babies born in England & Wales regardless of maternal country of birth. Slow pace of acculteration, physiological constraint or both? Analysis of routine data. *J Epidemiol Community Health* 2010; Published online 30 Nov 2010. doi:10.1136/jech2010.112516

16. Kelly Y, Panico L, Bartlet M et al. Why does birthweight vary among ethnic groups in the UK? Findings from the Millennium Cohort Study. *J Public Health (Oxf)* 2008;31(1):131-137.

17. West J; Lawlor DA; Fairley L et al. UK born Pakistani origin infants are relatively more adipose than White British infants: findings from 8704 mother-offspring pairs in the Born in Bradford prospective birth cohort. *J Epidemiol Community Health* 2013; doi:10.1136/jech-2012-20189.1

18. WrightJ, Small N, Raynor P et al. Cohort profile: the Born in Bradford multi-ethnic family cohort study. *Int J Epidemiol* 2013; Aug;42(4):978-91.

19. Office for National Statistics (ONS). Ethnic group statistics: a guide for the collection and classification of ethnicity data. London: Stationary Office; 2003.

20. World Health Organisation. Definition, diagnosis and classification of diabetes mellitus and its complications. Report of a WHO consultation. Part 1: diagnosis and classification of diabetes mellitus. WHO 1999 at http://whqlibdoc.who.int/hq/1999/who_ncd_ncs_99.2.pdf accessed on 18/03/2014.

For peer review only - http://bmjopen.bmj.com/site/about/guidelines.xhtml

21. Berthaud R. Family formation in multicultural Britain: diversity and change. In Ethnicity, social mobility and public policy, Loury GC, Modood T, Teles SM (Eds).Cambridge: Cambridge University Press; 2005.

22. Galobardes B, Shaw M, Lawlor DA et al. Indicators of socioeconomic position (Part 1). *J Epidemiol Community Health* 2006; 60(1):7-12.

23. Bradby H, Williams R. Is religion or culture the key feature in changes in substance use after leaving school? Young Punjabis and a comparison group in Glasgow. *Ethn and Health* 2006;11(3):307-324.

24. Hurcombe R, Bayley M, Goodman A. Ethnicity and alcohol. A review of the UK literature. Joseph Rowntree Foundation 2010.

25. Beishon S, Modood T, Virdee S. Ethnic minority families. London : Policy Studies Institute, 1998.

26. McKeigue PM, Pierpoint T, Ferrie JE et al. Relationship of glucose intolerance and hyperinsulinaemia to body fat pattern in South Asians and Europeans. *Diabetologia* 1992;35(8):785-91.

27. Ebrahim S, Kinra S, Bowen L et al. The effect of rural-to-urban migration on obesity and diabetes in India: a cross-sectional study. *PLoS Med* 2010 Apr 27;7(4).

28. Landman J, Cruickshank JK. A review of ethnicity, health and nutrition-related diseases in relation to migration in the United Kingdom. *Public Health Nutr* 2001;4 (2b):647-657.

29. Freathy RM, Kazeem GR, Morris RW et al. Genetic variation at *CHRNA5-CHRNA3-CHRNB4* interacts with smoking status to influence BMI. *Int J Epidemiol* 2011;40:1617-1628.

For peer review only - http://bmjopen.bmj.com/site/about/guidelines.xhtml

Table 1 Characteristics of women and infants (N=9450) by ethnic and generation group

	White British (UK & Ireland)	All Pakistani births	Pakistani sub-groups defined by place of birth of parents				
			Pakistani: Woman & partner UK born†	Pakistani: Woman UK born, partner SA born†	Pakistani: Partner UK born, woman SA born†	Pakistani: Woman & partner SA born†	Pakistani: Other
**Number**	3503	3656	383	992	876	1060	345
**Gestation at delivery (weeks)** Mean (sd)	39.0 (1.9)	39.0 (1.8)	39.0 (1.8)	38.9 (1.9)	39.0 (1.9)	39.1 (1.7)	39.1 (1.6)
**Births before 37 weeks N (%)**	209 (6.0)	204 (5.6)	22 (5.7)	63 (6.4)	50 (5.7)	52 (4.9)	17 (4.9)
**Mean birth weight in gm (sd)**	3346 (568)	3124(540)	3114 (538)	3100 (549)	3101 (537)	3160 (547)	3158 (497)
**Sex N (%)**							
**Male**	1808(52)	1851(51)	200(52)	504(51)	420(48)	535(51)	192(56)
**Female**	1695(48)	1805(49)	183(48)	488(49)	456(52)	525(49)	153(44)
**Maternal age** Mean (sd)	27 (6)	28 (5)	28 (5)	28 (5)	27 (5)	30 (5)	26 (5)
**Maternal height (m) Mean (sd)**	1.64 (0.06)	1.60 (0.06)	1.61 (0.05)	1.60 (0.06)	1.59 (0.05)	1.59 (0.05)	1.61 (0.06)
**Parity N (%)**							
0	1688 (48)	1157 (32)	155 (40)	331 (33)	253 (29)	254 (24)	164 (47)
1	1122 (32)	986 (26)	105 (27)	261 (26)	253 (29)	265 (25)	102 (30)
2	454 (13)	754 (21)	76 (20)	194 (20)	199 (23)	233 (22)	52 (15)
3	139 (4)	462 (13	34 (9)	125 (13)	111 (12)	178 (17)	14 (4)
4 or more	100 (3)	297 (8)	13 (4)	81 (8)	60 (7)	130 (12)	13 (4)
**Married N (%)**	1149 (33)	3571 (98)	364 (95)	974 (98)	862 (98)	1051 (99)	320 (93)
**Living with a partner N (%)**	2518 (72)	4702 (93)	352 (92)	898 (91)	852 (97)	1001 (95)	303 (88)
**Consumed alcohol during pregnancy N (%)**	266 (8)	0 (0)	0 (0)	0 (0)	0 (0)	0 (0)	0 (0)
**Total number of household members** Mean (sd)	3 (1)	5 (3)	5 (3)	5 (2)	6 (3)	5 (2)	5 (3)

†All four parents of the woman & her partner South Asian (SA) born

For peer review only - http://bmjopen.bmj.com/site/about/guidelines.xhtml

Table 2 Unadjusted and adjusted* odds ratios (95% CI) for socioeconomic characteristics for ethnic and generation groups

	**White British N=3503**	**All Pakistani births N=3656**	**Pakistani sub-groups defined by place of birth of parents**				
			**Pakistani: Woman & partner UK born† N=383**	**Pakistani: Woman UK born, partner SA born† N=992**	**Pakistani: Partner UK born, woman SA born† N=876**	**Pakistani: Woman &partner SA born† N=1060**	**Pakistani: Other N=345**
**In employment** Number (%)	**2272 (65)**	**881 (24)**	175 (46)	388 (39)	81 (9)	132 (12)	105 (30)
Unadjusted OR	**1**	**0.17 (0.16, 0.19)**	0.46 (0.37, 0.56)	0.35 (0.30, 0.40)	0.06 0.04, 0.07)	0.08 (0.06, 0.09)	0.24 (0.19, 0.30)
Adjusted OR*	**1**	**0.17 (0.15, 0.19)**	0.40 (0.32, 0.51)	0.38 (0.32, 0.44)	0.06 (0.04, 0.07)	0.06 (0.05, 0.08)	0.25 (0.19, 0.32)
**Educated post 16** Number (%)	**1601 (46)**	**1578 (43)**	235 (1)	473 (48)	306 (35)	401 (38)	163 (47)
Unadjusted OR	1	**0.90 (0.82, 0.99)**	1.89 (1.52, 2.34)	1.08 (0.94, 1.25)	0.64 (0.55, 0.74)	0.72 (0.63, 0.83)	1.06 (0.85, 1.33)
Adjusted OR*	1	**1.15 (1.04, 1.27)**	2.14 (1.70, 2.68)	1.37 (1.18, 1.59)	0.86 (0.73, 1.02)	0.88 (0.75, 1.03)	1.39 (1.11, 1.76)
**In receipt of means tested benefits**** Number (%)	**1334 (38)**	**1742 (48)**	163 (43)	534 (54)	387 (44)	523 (49)	135 (39)
Unadjusted OR	**1**	**1.48 (1.35, 1.63)**	1.20 (0.97, 1.49)	1.90 (1.64, 2.19)	1.29 (1.11, 1.49)	1.58 (1.38, 1.89)	1.05 (0.83, 1.31)
Adjusted OR*	**1**	**0.97 (0.87, 1.09)**	1.02 (0.79, 1.30)	1.42 (1.20, 1.67)	0.71 (0.60, 0.84)	0.91 (0.78, 1.08)	0.84 (0.65, 1.09)
**Housing tenure: owns/part-owns (mortgage)** Number (%)	**1875 (54)**	**2600 (71)**	283 (74)	730 (74)	669 (76)	698 (66)	220 (64)
Unadjusted OR	**1**	**2.14 (1.94, 2.36)**	2.46 (1.94, 3.12)	2.42 (2.07, 2.83)	2.81 (2.37, 3.32)	1.67 (1.45, 1.93)	1.53 (1.21, 1.92)
Adjusted OR*	**1**	**2.30 (2.07, 2.56)**	2.49 (1.95, 3.18)	2.60 (2.20, 3.06)	3.35 (2.80, 3.99)	1.55 (1.32, 1.80)	2.02 (1.60, 2.57)

†All four parents of the woman & her partner South Asian (SA) born

*Adjusted for maternal age; parity

** Any of: Income Support; Job Seekers Allowance; Working Tax Credit; Housing Benefits

For peer review only - http://bmjopen.bmj.com/site/about/guidelines.xhtml

Table 3 Unadjusted and adjusted* mean difference / odds ratios (95% CI) for lifestyle characteristics for ethnic and generation groups

	**White British N=3503**	**All Pakistani births N=3656**	**Pakistani sub-groups defined by place of birth of parents**				
			**Pakistani: Woman & partner UK born† N=383**	**Pakistani: Woman UK born, partner SA born† N=992**	**Pakistani: Partner UK born, woman SA born† N=876**	**Pakistani: Woman & partner SA born† N=1060**	**Pakistani: Other N=345**
**BMI at start of pregnancy** Mean (sd)	**26.8 (5.9)**	**25.7 (5.4)**	24.3 (4.6)	26.7 (5.7)	24.4 (4.7)	26.4 (5.6)	25.4 (5.3)
Unadjusted mean difference	**0**	**-1.15 (-1.41, -0.88)**	-2.53 (-3.13, -1.94)	-0.15 (-0.55, 0.25)	-2.44 (-2.86, -2.02)	-0.43 (-0.82, -0.04)	-1.40 (-2.02, -0.77)
Adjusted mean difference: Model 1*	**0**	**-1.75 (-2.01, -1.49)**	-2.84 (-3.41, -2.26)	-0.73 (-1.12, -0.34)	-2.95 (-3.36, -2.54)	-1.49 (-1.88, -1.10)	-1.22 (-1.83, -0.62)
Adjusted mean difference: Model 2**	**0**	**-1.12 (-1.43, -0.81)**	-2.32 (-2.92, -1.72)	-0.35 (-0.76, 0.07)	-2.22 (-2.69, -1.75)	-0.99 (-1.43, -0.57)	-0.77 (-1.39, -0.15)
**Smoked during pregnancy** Number (%)	**1183 (34)**	123 (3)	25 (7)	47 (5)	7 (0.8)	18 (2)	26 (8)
Unadjusted OR	**1**	**0.07 (0.06, 0.08)**	0.14 (0.09, 0.21)	0.09 (0.07, 0.13)	0.02 (0.01, 0.03)	0.03 (0.02, 0.05)	0.16 (0.11, 0.24)
Adjusted OR: Model 1*	**1**	**0.06 (0.05, 0.07)**	0.13 (0.09, 0.20)	0.09 (0.06, 0.12)	0.01 (0.01, 0.03)	0.03 (0.02, 0.05)	0.12 (0.08, 0.19)
Adjusted OR: Model 2**	**1**	**0.06 (0.05, 0.08)**	0.15 (0.09, 0.23)	0.09 (0.07, 0.13)	0.01 (0.01, 0.03)	0.03 (0.02, 0.05)	0.13 (0.08, 0.19)

†All four parents of the woman & her partner South Asian (SA) born

*Adjusted for maternal age; parity
**Adjusted for maternal age; parity; employment status; level of education, receipt of means tested benefits; housing tenure

For peer review only - http://bmjopen.bmj.com/site/about/guidelines.xhtml

Table 4 Unadjusted and adjusted* mean difference / odds ratios (95% CI) for health related pregnancy characteristics for ethnic and generation groups

	White British N=3503	All Pakistani births N=3656	Pakistani sub-groups defined by place of birth of parents				
			Pakistani: Woman & partner UK born† N=383	Pakistani: Woman UK born, partner SA born† N=992	Pakistani: Partner UK born, woman SA born† N=876	Pakistani: Woman & partner SA born† N=1060	Pakistani: Other N=345
**Hypertensive disorders of pregnancy**							
Number (%)	**239 (7)**	**188 (5)**	16 (4)	51 (5)	43 (5)	66 (6)	12 (3)
Unadjusted OR	**1**	**0.74 (0.61, 0.90)**	0.59 (0.35, 0.99)	0.74 (0.54, 1.01)	0.70 (0.51, 0.98)	0.91 (0.68, 1.20)	0.49 (0.27, 0.89)
Adjusted OR: Model 1*	**1**	**0.82 (0.67, 1.01)**	0.62 (0.37, 1.04)	0.82 (0.59, 1.12)	0.85 (0.61, 1.19)	0.99 (0.74, 1.33)	0.56 (0.31, 1.01)
Adjusted OR: Model 2**	**1**	**0.82 (0.64, 1.04)**	0.62 (0.36, 1.06)	0.81 (0.58, 1.13)	0.87 (0.59, 1.29)	1.01 (0.73, 1.40)	0.56 (0.31, 1.03)
Adjusted OR: Model 3***	**1**	**0.87 (0.67, 1.13)**	0.78 (0.45, 1.35)	0.80 (0.56, 1.14)	1.06 (0.70, 1.61)	1.06 (0.75, 1.49)	0.57 (0.31, 1.06)
**Gestational diabetes**							
Number (%)	**172 (5)**	406 (11)	30 (8)	96 (10)	92 (11)	159 (15)	29 (8)
Unadjusted OR	**1**	2.42 (2.01, 2.91)	1.65 (1.09, 2.46)	2.07 (1.59, 2.69)	2.27 (1.74, 2.96)	3.42 (2.72, 4.29)	1.78 (1.18, 2.68)
Adjusted OR: Model 1*	**1**	2.41 (1.98, 2.94)	1.66 (1.10, 2.49)	2.07 (1.58, 2.71)	2.54 (1.92, 3.35)	3.01 (2.36, 3.83)	2.24 (1.47, 3.41)
Adjusted OR: Model 2**	**1**	2.28 (1.82, 2.86)	1.66 (1.09, 2.53)	1.98 (1.49, 2.64)	2.47 (1.79, 3.39)	2.89 (2.20, 3.82)	2.21 (1.44, 3.40)
Adjusted OR: Model 3***	**1**	2.38 (1.86, 3.03)	1.89 (1.23, 2.92)	1.98 (1.46, 2.67)	2.82 (2.01, 3.97)	3.04 (2.27, 4.08)	2.29 (1.47, 3.56)

**Fasting glucose** Mean (sd)	**4.41 (0.41)**	**4.62 (0.62)**	4.54 (0.47)	4.58 (0.64)	4.54 (0.48)	4.73 (0.76)	4.60 (0.53)
Unadjusted mean difference	**0**	**0.20 (0.18, 0.23)**	0.13 (0.08, 0.19)	0.17 (0.14, 0.21)	0.13 (0.09, 0.17)	0.32 (0.29, 0.36)	0.19 (0.13, 0.25)
Adjusted mean difference: Model 1*	**0**	**0.18 (0.16, 0.21)**	0.12 (0.06, 0.17)	0.15 (0.11, 0.19)	0.12 (0.09, 0.16)	0.27 (0.24, 0.31)	0.22 (0.16, 0.27)
Adjusted mean difference: Model 2**	**0**	**0.18 (0.15, 0.21)**	0.12 (0.06, 0.18)	0.15 (0.11, 0.19)	0.12 (0.07, 0.16)	0.27 (0.23, 0.31)	0.22 (0.16, 0.27)
Adjusted mean difference: Model 3***	**0**	**0.20 (0.17, 0.24)**	0.17 (0.12, 0.23)	0.16 (0.12, 0.19)	0.17 (0.12, 0.21)	0.29 (0.25, 0.33)	0.23 (0.17, 0.29)
**Postload glucose** Mean (sd)	**5.47 (1.30)**	**5.89 (1.68)**	5.59 (1.35)	5.81 (1.58)	5.82 (1.50)	6.12 (2.02)	5.73 (1.45)
Unadjusted mean difference	**0**	**0.42 (0.35, 0.49)**	0.12 (-0.04, 0.28)	0.34 (0.23, 0.45)	0.35 (0.24, 0.46)	0.72 (0.62, 0.83)	0.26 (0.09, 0.42)
Adjusted mean difference: Model 1*	**0**	**0.37 (0.29, 0.44)**	0.08 (-0.07, 0.24)	0.29 (0.18, 0.39)	0.35 (0.24, 0.46)	0.58 (0.48, 0.69)	0.35 (0.19, 0.51)
Adjusted mean difference: Model 2**	**0**	**0.35 (0.27, 0.43)**	0.10 (-0.06, 0.26)	0.28 (0.17, 0.39)	0.33 (0.20, 0.46)	0.56 (0.44, 0.68)	0.34 (0.18, 0.51)
Adjusted mean difference: Model 3***	**0**	**0.37 (0.28, 0.45)**	0.18 (0.02, 0.34)	0.27 (0.16, 0.38)	0.39 (0.26, 0.52)	0.57 (0.45, 0.69)	0.35 (0.18, 0.52)
**Fasting insulin** Mean (sd)	**81.40 (46.72)**	**100.28 (62.76)**	92.66 (65.59)	100.76 (56.46)	91.75 (49.04)	106.11 (68.84)	111.09 (81.89)
Unadjusted mean difference	**0**	**18.88 (16.31, 21.45)**	11.26 (5.42, 17.09)	19.36 (15.46, 23.26)	10.36 (6.26, 14.45)	24.71 (20.91, 28.51)	29.69 (23.58, 35.81)
Adjusted mean difference: Model 1*	**0**	**18.08 (15.42, 20.74)**	10.98 (5.13, 16.82)	18.59 (14.64, 22.54)	9.67 (5.51, 13.83)	23.36 (19.43, 27.30)	29.69 (23.55, 35.82)
Adjusted mean difference: Model 2**	**0**	**21.29 (18.13, 24.45)**	14.01 (7.95, 20.08)	20.62 (16.40, 24.83)	13.53 (8.73, 18.34)	25.24 (20.89, 29.59)	32.01 (25.72, 38.31)
Adjusted mean difference: Model 3***	**0**	**25.71 (22.73, 28.69)**	24.44 (19.03, 29.86)	21.29 (17.47, 25.13)	23.27 (18.86, 27.68)	29.03 (25.04, 33.02)	34.79 (29.18, 40.39)

†All four parents of the woman & her partner South Asian (SA) born
*Adjusted for maternal age; parity
**Adjusted for maternal age; parity; employment status; level of education, receipt of means tested benefits; housing tenure
*** Adjusted for maternal age; parity; employment status; level of education, receipt of means tested benefits; housing tenure; early pregnancy BMI; smoking in pregnancy

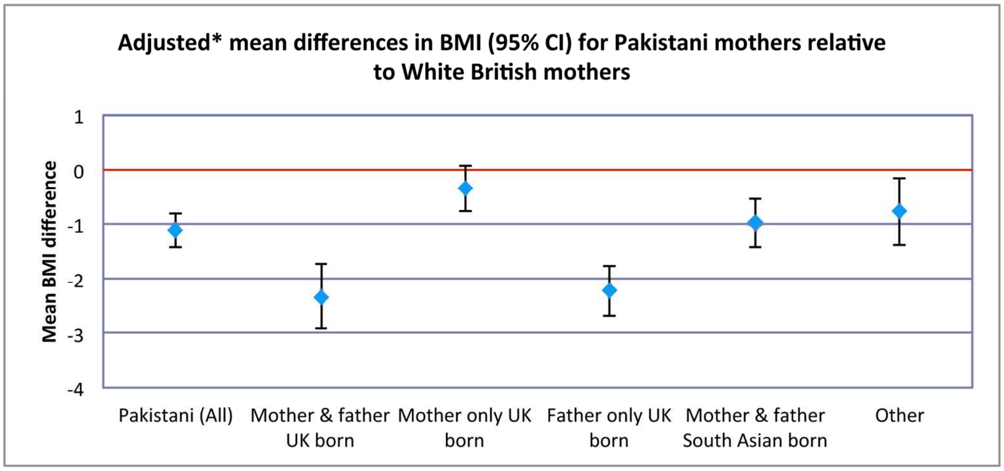

168x78mm (300 x 300 DPI)

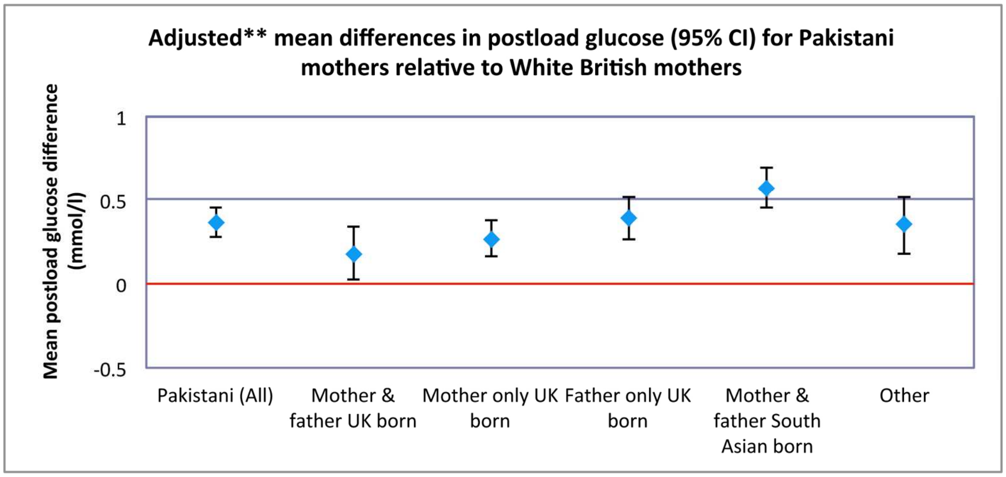

166x78mm (300 x 300 DPI)

For peer review only

STROBE Statement—Checklist of items that should be included in reports of ***cohort studies***

	Item No	Recommendation
**Title and abstract**	1	(*a*) The study's design is indicated in the title or the abstract (page 2)
		(*b*) Informative and balanced summary provided in abstract (page 2)
**Introduction**		
Background/rationale	2	Scientific background and rationale for the investigation being reported explained (page 4-6)
Objectives	3	Specific objectives stated (page 5)
**Methods**		
Study design	4	Key elements of study design presented (pages 6 & 7)
Setting	5	The setting, locations, and relevant dates, including periods of recruitment, exposure, follow-up, and data collection described (pages 6 & 7)
Participants	6	(*a*) Eligibility criteria and methods of follow-up given (page 6)
		(*b*) For matched studies, give matching criteria and number of exposed and unexposed N/A
Variables	7	All outcomes, exposures, predictors, potential confounders, and effect modifiers clearly defined (page 6 & 7)
Data sources/ measurement	8*	Sources of data and details of methods of assessment given. (pages 6 & 7)
Bias	9	Potential sources of bias discussed (page 12)
Study size	10	Study size described (page 6)
Quantitative variables	11	Means and sd /medians IQR were reported for continuous variables (pages 8 & 9)
Statistical methods	12	(*a*) All statistical methods, including those used to control for confounding described (page 7)
		(*b*) Describe any methods used to examine subgroups and interactions N/A
		(*c*) Explain how missing data were addressed : N/A
		(*d*) If applicable, explain how loss to follow-up was addressed N/A
		(*e*) Describe any sensitivity analyses N/A
**Results**		
Participants	13*	(a) Report numbers of individuals at each stage of study—eg numbers potentially eligible, examined for eligibility, confirmed eligible, included in the study, completing follow-up, and analysed (page 6)
		(b) Give reasons for non-participation at each stage N/A
		(c) Consider use of a flow diagram – described in methods
Descriptive data	14*	(a) Give characteristics of study participants (eg demographic, clinical, social) and information on exposures and potential confounders: included on page 6
		(b) Indicate number of participants with missing data for each variable of interest: N/A
		(c) Summarise follow-up time (eg, average and total amount) N/A (birth data)
Outcome data	15*	Report numbers of outcome events or summary measures over time: outcomes reported in results pages 8 & 9
Main results	16	(*a*) Give unadjusted estimates and, if applicable, confounder-adjusted estimates and their precision (eg, 95% confidence interval). Make clear which confounders were adjusted for and why they were included: included in main manuscript and tables
		(*b*) Report category boundaries when continuous variables were categorized: N/A
		(*c*) If relevant, consider translating estimates of relative risk into absolute risk for a

For peer review only - http://bmjopen.bmj.com/site/about/guidelines.xhtml

		meaningful time period N/A
Other analyses	17	Report other analyses done—eg analyses of subgroups and interactions, and sensitivity analyses N/A
**Discussion**		
Key results	18	Key results with reference to study objectives summarised (page 10)
Limitations	19	Limitations of the study, taking into account sources of potential bias or imprecision discussed. Limitations discussed (page 12)
Interpretation	20	Give a cautious overall interpretation of results considering objectives, limitations, multiplicity of analyses, results from similar studies, and other relevant evidence: included in discussion (pages10-12)
Generalisability	21	Discuss the generalisability (external validity) of the study results: included in discussion (page 12)
**Other information**		
Funding	22	Sources of funding and the role of the funders for the present study included (at end of manuscript)

For peer review only

For peer review only - http://bmjopen.bmj.com/site/about/guidelines.xhtml

BMJ Open

Differences in socioeconomic position, lifestyles and health related pregnancy characteristics between Pakistani and White British women in the Born in Bradford prospective cohort study: the influence of the woman's, her partner's and their parents' place of birth.

Journal:	*BMJ Open*
Manuscript ID:	bmjopen-2014-004805.R2
Article Type:	Research
Date Submitted by the Author:	13-May-2014
Complete List of Authors:	West, Jane; Bradford Teaching Hospitals NHS Foundation Trust, Bradford Institute for Health Research<br>Lawlor, Debbie; Department of Social Medicine, University of Bristol, Department of Social Medicine<br>fairley, lesley; Bradford Institute for Health Research, Data management<br>Wright, John; Department of Epidemiology and Public Health, Royal Infirmary Bradford
**Primary Subject Heading**:	Epidemiology
Secondary Subject Heading:	Public health
Keywords:	EPIDEMIOLOGY, ETHNICITY, LIFESTYLE

SCHOLARONE™ Manuscripts

For peer review only

Differences in socioeconomic position, lifestyle and health related pregnancy characteristics between Pakistani and White British women in the Born in Bradford prospective cohort study: the influence of the woman's, her partner's and their parents' place of birth.

West J *Specialty Registrar in Public Health*[1], **Lawlor DA** *professor of epidemiology* [2,3], **Fairley L** *statistician*[1], **Wright J** *professor of clinical epidemiology*[1]

[1] Bradford Institute for Health Research, Bradford Royal Infirmary, Bradford, BD9 6RJ, UK

[2] MRC Integrative Epidemiology Unit at the University of Bristol, UK

[3]School of Social and Community Medicine, University of Bristol, UK

Correspondence to jane.west@bthft.nhs.uk

For peer review only

For peer review only - http://bmjopen.bmj.com/site/about/guidelines.xhtml

ABSTRACT

Objective

To examine differences between Pakistani and White British women in relation to socioeconomic position, lifestyle and health related pregnancy characteristics, and to determine whether these differences vary depending on the woman's, her partner's and both of their parents' place of birth.

Design

Prospective cohort study.

Setting

Bradford, UK

Participants

3656 Pakistani and 3503 White British women recruited to the Born in Bradford study.

Main outcome measures

Socioeconomic position (employment status; level of education; receipt of benefits; housing tenure), lifestyle characteristics (BMI at the start of pregnancy; smoking during pregnancy) and health related pregnancy characteristics (hypertensive disorders of pregnancy; gestational diabetes; fasting glucose, postload glucose and fasting insulin at ~27 weeks gestation).

Results

Fewer Pakistani women were employed (OR 0.17 95% CI 0.15, 0.19), the difference being markedly less for UK born women. UK born Pakistani women were more likely, and South Asian born less likely, to be educated post 16 than White British women. Smoking was uncommon among Pakistani women, though the difference comparing UK born Pakistani women to White British women was less than for other groups. BMI was lower among Pakistani compared to White British women (adjusted mean difference -1.12 95% CI -1.43, -0.81) the difference greatest when partners were UK born irrespective of the woman's place of birth. Pakistani women had higher fasting and postload glucose (mean difference 0.20 mmol/l 95% CI 0.17, 0.24; 0.37 95% CI 0.28, 0.45), higher fasting insulin and were more likely to have gestational diabetes.

For peer review only - http://bmjopen.bmj.com/site/about/guidelines.xhtml

For peer review only

Conclusions

Our results suggest some socioeconomic, lifestyle and pregnancy characteristics could be beginning to change in response to migration to the UK, with generally beneficial changes i.e. improving education and employment prospects, lower BMI and no evidence that being UK born has further increased the risk of GDM, but some negative i.e. slight increases in smoking.

Strengths & limitations of this study

The strengths of this study include a large sample size, range of outcomes including oral glucose tolerance test data and detailed ancestry information.

We have for the first time, been able to examine ethnic differences in socioeconomic, lifestyle and pregnancy characteristics using information on the place of birth of women and their partners. We had also set out to explore differences based on all four grandparents but once we began analysing data it was apparent that for the majority of Pakistani women and their partners, all four of their parents were South Asian born. This limited our ability to explore differences across two generations, but highlights the persistence of strong family links in this community that have lived in Bradford for over 6 decades.

A potential limitation is that our results may not be generalizable to other South Asian populations and further work will be important to track these differences over future generations of UK South Asian migrants.

For peer review only

For peer review only - http://bmjopen.bmj.com/site/about/guidelines.xhtml

INTRODUCTION

Migration of South Asian populations to high income countries is generally thought to offer socioeconomic advantages in the form of improved education and employment opportunities, better housing and access to health care. However, improvements in environmental circumstances do not necessarily translate into improvements in health outcomes. Indeed, South Asian migrant populations to the UK experience an increased risk of maternal[1] and infant mortality[2] and some chronic diseases[3] compared with the UK population as a whole. This may reflect previous disadvantage associated with the country of origin which could persist over several generations, or could be a consequence of poor socioeconomic status within the host country. For example, UK South Asian communities are on average very poor[4]. That is, it could be that in comparison to those who do not migrate, there are improved health outcomes, but these remain poorer in comparison to the indigenous population. A further explanation is that the adoption of the unhealthy and sedentary lifestyles associated with acculturation or Westernisation, often characterised by low levels of physical activity[5], consumption of high calorie energy rich diets[6] and cigarette smoking[7,8], counteracts any potential health advantage of living in a higher income country. This may vary across different migrant communities but where this is the case, adoption of such lifestyles may be particularly harmful to South Asian individuals who for a given body mass index (BMI), have greater total and central adiposity and are known to be at greater risk of type 2 diabetes and cardiovascular disease than European adults[8-11].

Ethnic differences in socioeconomic position and lifestyle that might impact health during pregnancy could contribute to some of the known ethnic differences in pregnancy complications and perinatal outcomes. For example, they could contribute to the established greater risk of gestational diabetes (GDM)[12,13] and small for gestational age (SGA)[14-16] in South Asian compared to White British women. They could also drive ethnic differences in future generations either through intrauterine effects of maternal behaviours on these or as a result of the adoption of parental lifestyles by offspring and a lack of social migration. Previous studies have reported ethnic differences in socioeconomic and lifestyle characteristics between South Asian and White British women during pregnancy. Findings from the Millennium Cohort Study suggest South Asian women, in particular those originating from Pakistan and Bangladesh, are less likely to have formal educational qualifications, more likely to belong to lower socioeconomic groups and more likely to have never worked or be long term unemployed[7,16]. Marked differences in smoking and alcohol consumption between South Asian and White British women have also been reported[7,17]. Whilst outside pregnancy BMI is reportedly higher among South Asian women compared to White British women[18], we have previously reported that BMI is lower among Pakistani origin

For peer review only - http://bmjopen.bmj.com/site/about/guidelines.xhtml

pregnant women in the Born in Bradford (BiB) cohort[17]. Much less is known about maternal blood glucose and insulin in particular whether there are differences in these outcomes across generations of UK South Asian migrants. To our knowledge, no previous studies have examined ethnic differences in all these characteristics (socioeconomic, lifestyle, pregnancy) collectively which is important to identify areas where South Asian women may have better outcomes and those where European women may have better outcomes. This knowledge could support the delivery of appropriate antenatal care aimed at maximising maternal and child health in both White British and South Asian groups.

Furthermore, previous studies have not explored whether any identified ethnic differences during pregnancy are consistent when the mother's, her partner's and both of their parents' country of origin are taken into account. In a previous study, using data from the Born in Bradford cohort, which is used in this paper, we showed that birthweight was lower, but that birth fatness (assessed using skinfold thickness and cord blood leptin) was greater in Pakistani compared to White British infants[17]. We further showed that these differences did not differ by whether both the mother and her partner and all four of their parents were born in the UK, all born in South Asia or there was a mixed pattern between these two extremes[17]. Here, we extend that work to look at a range of socioeconomic position, lifestyle and pregnancy related outcomes, in order to understand whether in the context of place of birth of women and her closest family relatives, there are some ethnic differences that are reduced or some that are enhanced, and if so whether these would be beneficial or detrimental to health.

The aim of this study was to examine differences between Pakistani women and White British women in relation to socioeconomic position (employment status; level of education; receipt of means tested benefits; housing tenure), lifestyle characteristics (BMI at the start of pregnancy; smoking during pregnancy) and health related pregnancy characteristics (hypertensive disorders of pregnancy (HDP); gestational diabetes; fasting glucose, postload glucose and fasting insulin at ~27 weeks gestation), and to determine whether these differences vary depending upon the woman's, her partner's and both of their parents' place of birth.

METHODS

Participants

The Born in Bradford (BiB) study is a largely bi-ethnic prospective birth cohort study that recruited women during pregnancy and has followed them, their infants and their partners into the child's infancy. To be eligible for the study women had to attend booking clinic

For peer review only - http://bmjopen.bmj.com/site/about/guidelines.xhtml

between March 2007 and December 2010 and be booked to give birth in the city of Bradford. Full details of the study methodology have been previously reported[18]. Women were recruited at their oral glucose tolerance test (OGTT) appointment; all women booked for delivery in Bradford are offered a 75g OGTT (comprising fasting and 2 hour postload samples) at around 26 – 28 weeks gestation. Women who attended this appointment and agreed to take part in the study consented to the use of their obstetric medical records, had their height and weight recorded and completed an interviewer administered questionnaire. The questionnaire included questions relating to ethnicity, social and economic circumstances, smoking, alcohol, diet, education and employment and collected place of birth information for both parents and all four grandparents. Interviews were conducted in a range of South Asian languages (including Mirpuri, Bengali, Punjabi). Mirpuri is the most commonly spoken Asian language in Bradford but has no written script therefore questionnaires were transliterated, that is translated verbally to Mirpuri and then written phonetically, precisely as spoken to ensure that all interpreters translated it in the same way. Details of the language used to conduct the questionnaire were recorded. Ethics approval for the study was provided by Bradford Local Research Ethics Committee (ref 06/Q1202/48). Data were available for 11,113 women recruited to the BiB cohort. We excluded stillbirths (n=64) and infants born to parents of ethnic origin other than White British or Pakistani (n=1598). Of the remaining 9451 participants 7159 had complete data for all variables included in all models thus 3656 Pakistani and 3503 White British women are included in these analyses. Women with existing diabetes (0.5% of the BiB cohort) are not invited to attend for the glucose tolerance test as they are treated from the start of their pregnancy by an endocrine physician. As a result these women were not recruited at the same time as other participants and do not have some data, including parental place of birth. These women are therefore not included in these complete case analyses.

Woman's family member's place of birth

Ethnicity was self-reported at interview, with participants given response options based on UK Office of National Statistics guidance[19]. Women completed a detailed ancestry interview, which included details of the place of birth of themselves, their partner and all four parents of themselves and their partner. Family place of birth groups of the Pakistani infants were derived from these data as previously reported[17]. In the previous report, since our outcome of interest was infant birth size the groups were defined in terms of 'parents' and 'grandparents'. As our outcomes here are in pregnant women we have described them in relation to her, but the groups are essentially the same as the previous paper. Our aim in that previous paper, as here, was to examine differences across all possible groups based on place of birth of the woman, her partner and all four parents. Thus, we began by

For peer review only - http://bmjopen.bmj.com/site/about/guidelines.xhtml

determining numbers in all 64 possible combinations of these six family members. Having done that it was apparent that for almost all women, the four parents of the woman and her partner were South Asian born meaning that the analyses were based primarily on the woman's and her partner's place of birth. Overall, 90% of women fell into one of four main categories:

1. Woman and her partner UK born and all four of their parents South Asian born
2. Woman UK born, partner and all four of their parents South Asian born
3. Partner UK born, woman and all four parents South Asian born
4. Woman, her partner and four parents all South Asian born

The remaining 11% (n=345), including those with one or more of the woman's or her partner's parents being UK born or where their parents' place of birth was unknown, was combined to form one 'other' group.

Outcome measures

Socioeconomic

Information on socioeconomic indicators (employment, education, receipt of benefits, housing tenure) was obtained from the interview with the woman at recruitment. We equivalised the mother's highest educational qualifications (based on the qualification received and the country obtained) into one of several categories using UK NARIC (http://www.ecctis.co.uk/naric/default.aspx): *<5 GCSE equivalent, ≥5 GCSE equivalent, 'A' level equivalent, Higher than A-level equivalent, Other qualifications (e.g. City and Guilds, RSA/OCR, BTEC), Don't know, Foreign Unknown.* Don't know relates to the mother responding "don't know" during interview. Foreign Unknown relates to a qualification listed in the free text response but no level of qualification is given or the qualification listed cannot be equivalised to one of the above categories. For these analyses, women were categorised as having been educated beyond the age of 16 or not (i.e. *Higher than A-level equivalent, Other qualifications (e.g. City and Guilds, RSA/OCR, BTEC).* Receipt of means tested benefits was based on the mother or her household receiving any of: Income Support, Job Seekers Allowance, Working Tax Credit or Housing Benefit. Housing tenure was categorised according to whether the woman lived in a household where the home was either part-owned (i.e. mortgaged) or owned outright, or not (i.e. rented).

Lifestyle

BMI is used in these analyses as a proxy marker of lifestyle as it is an outcome that can potentially be influenced by changes or differences in lifestyle (in particular dietary choices

For peer review only - http://bmjopen.bmj.com/site/about/guidelines.xhtml

and levels of physical activity). At recruitment, women were weighed and their height measured (unshod and in light clothing) using SECA digital scales and a Leicester Height Measure respectively. Weight at first antenatal clinic assessment when women were around 12 weeks gestation (median 12 weeks, IQR 11, 14), was abstracted from the antenatal records and this weight together with height measured at recruitment, was used to calculate the woman's BMI so that this reflected early pregnancy BMI before substantial contribution from pregnancy and the growing fetus. Information on smoking was obtained at the questionnaire interview, with women categorised as having smoked cigarettes at any stage of their pregnancy or not. As none of the Pakistani origin women reported drinking alcohol, we were unable to include alcohol consumption as an outcome.

Health related pregnancy characteristics

Women were classified as hypertensive in pregnancy if they had a systolic measure ≥140 and a diastolic ≥90mmHg on 2 or more occasions after 20 weeks gestation; information on this was obtained from the antenatal records. Fasting and postload glucose and fasting insulin were obtained from the OGTT plasma samples which were assayed immediately after sampling at the biochemistry department of Bradford Royal Infirmary using the glucose oxidase method on Siemen's Advia 2400 chemistry autoanalysers. GDM was defined using the fasting and postload glucose according to WHO criteria[20] at the time these women were pregnant as either a fasting glucose ≥6.1mmol/L or a two-hour postload glucose ≥7.8mmol/L. Women with existing diabetes prior to pregnancy did not complete an OGTT and are not included in this sample.

Statistical analyses

All analyses were performed using Stata (version 12.1). We used univariable regression to examine the association of ethnicity and family place of birth group with outcomes. Included predictor variables were decided a priori based on existing evidence and knowledge. Logistic regression was used for binary outcomes and linear regression for continuous outcomes, with the White British group used as the reference for all analyses, i.e. we compared outcomes in 'all' Pakistani women and then each of the five family place of birth subgroups of Pakistani women to outcomes in White British women. The rationale for this is because our aim is primarily to compare all Pakistani origin women with White British women and then to compare subgroups based on place of birth with the same reference group of White British women to see if place of birth of the Pakistani women influences the extent to which they differ or not from the indigenous population. In all adjusted analyses we adjusted for maternal age and parity (Model 1). For the lifestyle outcomes (early pregnancy BMI; smoking) we also adjusted for each of the indicators of socioeconomic position in order

For peer review only - http://bmjopen.bmj.com/site/about/guidelines.xhtml

For peer review only

to explore the extent of any differences in these lifestyles might reflect ethnic differences in socioeconomic position (Model 2). For the health related pregnancy characteristics we also adjusted for socioeconomic indicators (Model 2) and also for the lifestyle characteristics (BMI; smoking) (Model 3), to explore whether these explained any of the differences. When age and BMI were included in models as covariables they were used as continuous variables. Existing literature supports their linear associations with outcomes and we confirmed this graphically. For all multivariable models we examined the residuals and these were all found to be approximately normal. Further, we checked potential problems with collinearity in each model by assessing variance inflation and found that this was lower than 2 for all independent variables in all models.

RESULTS

The characteristics of White British and Pakistani origin women are shown in Table 1. There was little difference between the two ethnic groups in mean gestation, premature births and infant sex. As reported in our previous paper[17], birthweight of their infant was markedly lower in Pakistani compared to White British women when all Pakistani origin women were combined and also when compared by subgroups based on place of birth. On average, Pakistani origin women were slightly older, in particular when both parents were South Asian born, markedly more likely to be married and lived within larger households than White British women. These differences were similar across all generation groups. Pakistani women were shorter than White British women but the difference was less when women were UK born. There were also some differences in parity across Pakistani generation groups, for example, parity was on average lowest when both parents were UK born and highest when both parents were born in South Asia.

The odds of being in employment for Pakistani women were 83% lower than White British women (adjusted OR 0.17 95% CI 0.15, 0.19), but there were differences by family place of birth (Table 2). These odds were 94% less for those who were South Asian born but this difference reduced to 60% for Pakistani women when both they and their partner were UK born. Following adjustment for maternal age and parity, Pakistani women as a whole were more likely to be educated beyond the age of 16 than White British women (OR 1.15 95% CI 1.04, 1.27), however there were marked differences across family place of birth groups with women who were South Asian born being less likely, and those who were UK born being more likely compared to White British women, to be educated beyond 16 years. Being in receipt of means tested benefits was similar in both ethnic groups when Pakistani women were assessed as a whole (adjusted OR 0.97 95% CI 0.87, 1.09) although for Pakistani women who were UK born with a South Asian partner there were increased odds of

For peer review only - http://bmjopen.bmj.com/site/about/guidelines.xhtml

receiving benefits. Compared to White British women, Pakistani women were considerably more likely to own or part own their home (adjusted OR 2.30 95% CI 2.07, 2.56) and this was consistent across all family place of birth groups.

Table 3 shows the unadjusted and adjusted (Models 1 and 2) ethnic difference in lifestyle characteristics. Pakistani women had a lower BMI than White British women (adjusted [Model 2] mean difference -1.12 95% CI -1.43, -0.81) but the difference was much greater when the woman's partner was UK born irrespective of where the woman herself was born (Figure 1). The odds of smoking for Pakistani women were around 94% less and this was similar across generation groups other than when both the woman and her partner were UK born in which case they were 85% less. None of the Pakistani women reported drinking any alcohol during pregnancy (0%), whereas 8% of White British women drank during pregnancy.

In Table 4 the unadjusted and adjusted (Models 1-3) ethnic difference in pregnancy characteristics is shown. Fewer Pakistani women in general had HDP (adjusted [Model 3] OR 0.87 95% CI 0.67, 1.13), although this result was imprecisely estimated with wide confidence intervals that included the null. This was not consistent across all family place of birth groups for example, women who were South Asian born were slightly more likely to have HDP than White British women and this was the case in all 3 adjusted models. Pakistani women were more likely to have GDM and higher fasting and postload glucose and fasting insulin than White British women and these differences were broadly similar across all 3 models of adjustment. There were some differences by family place of birth group, for example, the difference in postload glucose between Pakistani and White British women was far greater when the woman and her partner were born in South Asia than when both were UK born (adjusted mean difference [Model 3] 0.57 95% CI 0.45, 0.69 and 0.18 95% CI 0.02, 0.34 respectively and Figure 2).

DISCUSSION

We have shown differences across a range of socioeconomic, lifestyle and pregnancy characteristics between Pakistani and White British origin women and that these vary depending on whether Pakistani women are born in the UK or South Asia. We have for the first time, been able to consider not only the woman's place of birth, but also her partner's and both of their parents' place of birth; though after preliminary analyses it was clear that for the majority of women and their partners, all four of their parents were South Asian born. This provides important information about how these differences might be reduced or even enhanced with greater acculturation over generations. For example, the odds of Pakistani

For peer review only - http://bmjopen.bmj.com/site/about/guidelines.xhtml

women as a whole being in employment, were 83% less than White British women, but across generation groups this difference varied from 60% when both the woman and her partner were born in the UK, to 94% when both the woman and her partner were South Asian born. Likewise, we found interesting differences in education attainment between Pakistani and White British women. Overall, Pakistani women were slightly more likely to have been educated beyond the age of 16, but this was driven by UK born Pakistani women, especially those with a UK born partner who were twice as likely as White British women to have completed education beyond age 16. By contrast, South Asian born Pakistani women, irrespective of their partner's place of birth, were less likely than White British women to have been educated beyond the age of 16. This could reflect a positive effect of migration and acculturation on social mobility which likely plays a part in the employment differences described above and is consistent with previous reports[7,21]. Whilst differences in employment and education by place of birth suggest the adoption of some British lifestyle characteristics, the tendency of Pakistani women to live within larger households and to be more likely to own or part-own their own home, suggests that the traditional culture of living within extended families has been maintained across all place of birth sub-groups of Pakistani women.. Living with an extended family could have considerable benefits for the mother and her offspring, such as childcare support and greater social capital, but could also result in overcrowding and potential detrimental impacts of this on health[22]. Early analyses using data from BiB suggests that living with more family members does not lead to greater family social capital (Cabieses B, unpublished data 2013). Pakistani women who were born in the UK but had a South Asian born partner, were more likely to claim benefits compared to White British women than those who were South Asian born which is surprising given that they tend to be more likely to be in employment. This might reflect a tendency for South Asian born partners to be in lower paid employment reducing total household income, or that poorer command of the English language (likely amongst those Pakistani women who were South Asian born and were less likely to claim benefits compared to White British women) is a barrier to accessing services and social support.

Greater social migration, for example coming to the UK for social reasons, has been associated with increased uptake of lifestyle characteristics of the host country such as smoking and alcohol consumption[7]. We report a similar trend in that UK born Pakistani origin women were more likely to smoke than South Asian born women, but smoking was still uncommon among all Pakistani women compared to White British women and none of them reported any alcohol consumption during pregnancy. Thus, the increase in these harmful health behaviours over generations in some migrant groups, whilst showing some signs of change, appears minimal among Pakistani women. This may reflect persisting cultural or

For peer review only - http://bmjopen.bmj.com/site/about/guidelines.xhtml

religious influences[23,24] and could be related to the fact that for the majority of women, both of their parents and their partners parents were South Asian born We found BMI to be slightly lower among Pakistani origin women compared to White British women although there were interesting differences across family place of birth groups. The finding that the difference in BMI between Pakistani and White British women was markedly greater for Pakistani women with a UK born partner, irrespective of their own place of birth, than for women with a South Asian born partner is particularly striking. One possible explanation is that within this population, partners/husbands have a particularly dominant role[25]. Thus, the lifestyle choices of the family or household will be driven mostly by the social norms and habits of the partner. In the case of men born in the UK, these are likely to be influenced by western culture which promotes a lower BMI as both healthy and attractive. Similarly, having been brought up and educated in the UK, they may be more likely to participate in organised physical activity and also may be more receptive to UK public health campaigns.

Health related pregnancy characteristics may be the most important to the long-term health of South Asian migrants in the UK, particularly in relation to the association of these characteristics with cardiovascular disease and type 2 diabetes[26]. We report a number of differences between Pakistani women and White British women in HDP, glucose tolerance, fasting insulin and GDM. Pakistani women as a whole group were less likely to have HDP, although this was not consistent across family subgroups, but they were more than twice as likely to have GDM. Consistent with these higher rates of GDM, Pakistani women had higher fasting and postload glucose and higher fasting insulin than White British women. These findings are similar to those from previous studies showing that South Asian women are more likely to have GDM than White European women[12,13]. They are also consistent with considerable evidence that adult non-pregnant women and men have a higher risk of insulin resistance and type 2 diabetes[9-11,26]. We found that the increased likelihood of Pakistani women having GDM compared to White British women was greatest for South Asian born women. We also found that the mean difference in fasting and postload glucose and fasting insulin relative to White British women was substantively greater when the woman and her partner were both born in South Asia. This is somewhat surprising as evidence suggests that the increased risk of insulin resistance and type 2 diabetes in South Asian adults compared to White Europeans is largely amongst those in urban (rather than rural) areas of South Asia[27], or in those who have migrated to Western countries[9,28]. We might therefore have expected the increase to be greater amongst those who were UK born. The difference between our findings and these previous studies of non-pregnant migrants[9,26,27], might be explained by differences in the population studied, with many of these previous studies being of Indian, or mixed rather than Pakistani origin. Pakistani

For peer review only - http://bmjopen.bmj.com/site/about/guidelines.xhtml

migrants in general tend to be poorer, shorter and weigh less, and the Pakistani women in this study have lower BMI than the White British women. For religious and cultural reasons Pakistani women remain unlikely to smoke or drink alcohol which could influence their glucose tolerance, although smoking is related to lower BMI and therefore would be expected to reduce glucose tolerance[29]. It might also be that whilst insulin resistance and diabetes in the general population are enhanced in those who migrate and particularly with greater duration of migration, in pregnancy the impact of place of birth or time since migration differs. We are not aware of other studies with equivalent data to explore this further, but it would be interesting to see if this finding is replicated.

The key strengths of this study are the large sample size, range of outcomes we have been able to examine, including OGTT data, and the detailed information on place of birth. To our knowledge this is the first study to examine differences between Pakistani and White British women in relation to socioeconomic, lifestyle and pregnancy characteristics using detailed information on the place of birth of women and their partners. We had hoped to explore three generations of Pakistani migrants to Bradford, but for almost all the women in this study, their parents and the parents of their partner were born in South Asia. However, this is in itself an interesting finding and useful for meeting future health needs in the city. It might also explain some of our findings in relation to the persistence of some characteristics across family place of birth subgroups. A potential limitation of our study was the inability to include other South Asian groups in our analyses (Indian and Bangladeshi) due to small numbers within our cohort. On the one hand examining a specific South Asian population (Pakistani) reduces the problem of heterogeneity between South Asian groups but at the same time it may limit the generalisability of our results to other South Asian populations. Our analyses have not accounted for South Asians who migrate to the UK in childhood and may be resident in the UK for much of their development and education, which could potentially dilute any differences between the Pakistani place of birth groups. Within BiB information regarding the age at which an individual migrated to the UK is only available for women (not their partner or parents) therefore we were not able to account for this in our family place of birth groups. We were not able to validate self-report of smoking or alcohol consumption in pregnancy for either the Pakistani or White British women. If reporting bias, which might occur because of the stigma associated with these behaviours in pregnancy, is similar in each ethnic group it should not bias the comparisons that are the main focus of this paper. Many of the researchers who collected interview data were of Pakistani origin and it is possible that this may have resulted in greater under-reporting in the Pakistani origin women. However, the prevalence of these behaviours in this study is similar to those in other studies of Pakistani women[7].

For peer review only - http://bmjopen.bmj.com/site/about/guidelines.xhtml

In summary, we have found some evidence that the difference in some of these characteristics between Pakistani and White British women may be changing in response to migration to the UK, in that differences were seen most often in those where the woman or her partner were UK born. Several of these differences would be beneficial to health and wellbeing. For example, Pakistani women born in the UK were more likely than White British women to be educated beyond age 16. UK born Pakistani women were also more similar to White British women in terms of employment and there was no evidence that being UK born increased their risk of GDM or glucose intolerance. On the other hand, whilst overall prevalence of smoking in Pakistani women in all groups was very small, the difference between them and White British women was least when they were UK born. We have also identified differences that vary according to the woman's partner's place of birth, for example BMI is lower among Pakistani women with a UK born partner. Further work is needed that continues to track these important ethnic differences over future generations to support the delivery of appropriate antenatal care.

Words: Abstract:300 ; Main text: 4800

For peer review only

Acknowledgements

Born in Bradford is only possible because of the enthusiasm and commitment of the children and parents in Born in Bradford. We are grateful to all participants, health professionals and researchers who have made Born in Bradford happen.

Funding

J West was funded by a UK Medical Research Council (MRC) Special Training Fellowship in Health of the Public and Health Services Research (MRC GO601712). DA Lawlor works in a unit that receives UK MRC funding (MC_UU_12013/5). BiB receives core infrastructure funding from the Wellcome Trust (WT101597MA).

Author contributions

J West, DA Lawlor and J Wright conceived the study idea, obtained funds, developed the statistical analysis plan, were involved in managing the data collection and wrote the initial drafts of the paper; J West undertook the main analysis with input from L Fairley and supervision from DA Lawlor and J Wright. J West acts as guarantor.

Competing interests

All authors declare no competing interests

Data sharing

Scientists are encouraged and able to use BiB data. Data requests are made to the BiB executive using the form available from the study website www.borninbradford.nhs.uk (please click on "Science and Research" to access the form). Guidance for researchers and collaborators, the study protocol and the data collection schedule are all available via the website. All requests are carefully considered and accepted where possible.

Declaration of transparency

J West affirms that the manuscript is an honest, accurate, and transparent account of the study being reported; that no important aspects of the study have been omitted; and that any discrepancies from the study as planned (and, if relevant, registered) have been explained.

This is an Open Access article distributed in accordance with the Creative Commons Attribution Non Commercial (CC BY-NC 3.0) license, which permits others to distribute, remix, adapt, build upon this work non-commercially, and license their derivative works on different terms, provided the original work is properly cited and the use is non-commercial. See:http://creativecommons.org/licenses/by-nc/3.0/

For peer review only - http://bmjopen.bmj.com/site/about/guidelines.xhtml

FIGURE LEGENDS

Figure 1 Adjusted mean differences in BMI for Pakistani women relative to White British women

*Model 2: Adjusted for maternal age; parity; employment; post-16 education; receipt of means tested benefits; housing tenure

Figure 2 Adjusted mean differences in postload glucose for Pakistani women relative to White British women

**Model 3: Adjusted for maternal age; parity; employment; post-16 education; receipt of means tested benefits; housing tenure; early pregnancy BMI; smoking in pregnancy

For peer review only

References

1. Saving Mothers' Lives: Reviewing maternal deaths to make motherhood safer: 2006-2008. *BJOG* 2011 Mar 1;118 Suppl 1:1–203

2. Office for National Statistics (ONS). Gestation-specific infant mortality in England and Wales, 2010. London, ONS 2012. At http://www.ons.gov.uk/ons/dcp171778_282579.pdf accessed on 18/03/2014.

3. Bhala N, Zaman MS. Preventing premature mortality in chronic diseases for South Asians in the UK and beyond. *J R Soc Med* 2009;102(11): 459–463.

4. Nazroo, J. Ethnicity, class and health. London, Policy Studies Institute, 2001.

5. Williams ED, Stamatakis E, Chandola T et al. Assessment of physical activity levels in South Asians in the UK: findings from the Health Survey for England. *JECH* 2011;65:517-21.

6. Patel JV, Vyas A, Cruickshank JK et al. Impact of migration on coronary heart disease risk factors: comparison of Gujaratis in Britain and their contemporaries in villages of origin in India. *Atherosclerosis* 2006;185:297-306.

7. Hawkins SS, Lamb K, Cole TJ et al. Influence of moving to the UK on maternal health behaviours: prospective cohort study. *BMJ* 2008; 336:1052.

8. Sproston, K; Mindell, JE. Health Survey for England 2004. The health of minority ethnic groups. HSE 2006. Information Centre, London.
9. Barnett AH, Dixon AN, Bellary S, et al. Type 2 diabetes and cardiovascular risk in the UK south Asian community. *Diabetologia* 2006;49(10):2234-46.
10. Mather HM, Keen H. The Southall Diabetes survey: prevalence of known diabetes in Asians and Europeans. *BMJ* 1985;291:1081-4.

11. Gholap N, Davies M, Patel K et al. Type 2 diabetes and cardiovascular disease in South Asians. *Prim Care Diabetes* 2011;5(1):45-56. Epub 2010/09/28.

12. Dornhurst A, Pateson CM, Nicholls JS et al. High prevalence of gestational diabetes mellitus in women from ethnic minority groups. *Diabet Med* 1992; Nov;9(9):820-5

13. Makgoba M, Savvidou MD, Steer PJ. An analysis of the interrelationship between maternal age, body mass index and racial origin in the development of gestational diabetes mellitus. *BJOG* 2012; Feb;119(3):276-82.

14. Margetts BM, Mohd Yusof S, Al Dallal Z et al. Persistence of lower birth weight in second generation South Asian babies born in the United Kingdom. *J Epidemiol Community Health* 2002 Sep;56(9):684-7.

15. Leon DA, Moser K. Low birth weight persists in South Asian babies born in England & Wales regardless of maternal country of birth. Slow pace of acculteration, physiological constraint or both? Analysis of routine data. *J Epidemiol Community Health* 2010; Published online 30 Nov 2010. doi:10.1136/jech2010.112516

For peer review only - http://bmjopen.bmj.com/site/about/guidelines.xhtml

16. Kelly Y, Panico L, Bartlet M et al. Why does birthweight vary among ethnic groups in the UK? Findings from the Millennium Cohort Study. *J Public Health (Oxf)* 2008;31(1):131-137.

17. West J; Lawlor DA; Fairley L et al. UK born Pakistani origin infants are relatively more adipose than White British infants: findings from 8704 mother-offspring pairs in the Born in Bradford prospective birth cohort. *J Epidemiol Community Health* 2013; doi:10.1136/jech-2012-20189.1

18. WrightJ, Small N, Raynor P et al. Cohort profile: the Born in Bradford multi-ethnic family cohort study. *Int J Epidemiol* 2013; Aug;42(4):978-91.

19. Office for National Statistics (ONS). Ethnic group statistics: a guide for the collection and classification of ethnicity data. London: Stationary Office; 2003.

20. World Health Organisation. Definition, diagnosis and classification of diabetes mellitus and its complications. Report of a WHO consultation. Part 1: diagnosis and classification of diabetes mellitus. WHO 1999 at http://whqlibdoc.who.int/hq/1999/who_ncd_ncs_99.2.pdf accessed on 18/03/2014.

21. Berthaud R. Family formation in multicultural Britain: diversity and change. In Ethnicity, social mobility and public policy, Loury GC, Modood T, Teles SM (Eds).Cambridge: Cambridge University Press; 2005.

22. Galobardes B, Shaw M, Lawlor DA et al. Indicators of socioeconomic position (Part 1). *J Epidemiol Community Health* 2006; 60(1):7-12.

23. Bradby H, Williams R. Is religion or culture the key feature in changes in substance use after leaving school? Young Punjabis and a comparison group in Glasgow. *Ethn and Health* 2006;11(3):307-324.

24. Hurcombe R, Bayley M, Goodman A. Ethnicity and alcohol. A review of the UK literature. Joseph Rowntree Foundation 2010.

25. Beishon S, Modood T, Virdee S. Ethnic minority families. London : Policy Studies Institute, 1998.

26. McKeigue PM, Pierpoint T, Ferrie JE et al. Relationship of glucose intolerance and hyperinsulinaemia to body fat pattern in South Asians and Europeans. *Diabetologia* 1992;35(8):785-91.

27. Ebrahim S, Kinra S, Bowen L et al. The effect of rural-to-urban migration on obesity and diabetes in India: a cross-sectional study. *PLoS Med* 2010 Apr 27;7(4).

28. Landman J, Cruickshank JK. A review of ethnicity, health and nutrition-related diseases in relation to migration in the United Kingdom. *Public Health Nutr* 2001;4 (2b):647-657.

For peer review only - http://bmjopen.bmj.com/site/about/guidelines.xhtml

29. Freathy RM, Kazeem GR, Morris RW et al. Genetic variation at *CHRNA5-CHRNA3-CHRNB4* interacts with smoking status to influence BMI. *Int J Epidemiol* 2011;40:1617-1628.

For peer review only

For peer review only - http://bmjopen.bmj.com/site/about/guidelines.xhtml

Table 1 Characteristics of women and infants (N=9450) by ethnic and generation group

	White British (UK & Ireland)	All Pakistani births	Pakistani sub-groups defined by place of birth of parents				
			Pakistani: Woman & partner UK born†	Pakistani: Woman UK born, partner SA born†	Pakistani: Partner UK born, woman SA born†	Pakistani: Woman & partner SA born†	Pakistani: Other
**Number**	3503	3656	383	992	876	1060	345
**Gestation at delivery (weeks)** Mean (sd)	39.0 (1.9)	39.0 (1.8)	39.0 (1.8)	38.9 (1.9)	39.0 (1.9)	39.1 (1.7)	39.1 (1.6)
**Births before 37 weeks N (%)**	209 (6.0)	204 (5.6)	22 (5.7)	63 (6.4)	50 (5.7)	52 (4.9)	17 (4.9)
**Mean birth weight in gm (sd)**	3346 (568)	3124(540)	3114 (538)	3100 (549)	3101 (537)	3160 (547)	3158 (497)
**Sex N (%)**							
**Male**	1808(52)	1851(51)	200(52)	504(51)	420(48)	535(51)	192(56)
**Female**	1695(48)	1805(49)	183(48)	488(49)	456(52)	525(49)	153(44)
**Maternal age** Mean (sd)	27 (6)	28 (5)	28 (5)	28 (5)	27 (5)	30 (5)	26 (5)
**Maternal height (m) Mean (sd)**	1.64 (0.06)	1.60 (0.06)	1.61 (0.05)	1.60 (0.06)	1.59 (0.05)	1.59 (0.05)	1.61 (0.06)
**Parity N (%)**							
0	1688 (48)	1157 (32)	155 (40)	331 (33)	253 (29)	254 (24)	164 (47)
1	1122 (32)	986 (26)	105 (27)	261 (26)	253 (29)	265 (25)	102 (30)
2	454 (13)	754 (21)	76 (20)	194 (20)	199 (23)	233 (22)	52 (15)
3	139 (4)	462 (13	34 (9)	125 (13)	111 (12)	178 (17)	14 (4)
4 or more	100 (3)	297 (8)	13 (4)	81 (8)	60 (7)	130 (12)	13 (4)
**Married N (%)**	1149 (33)	3571 (98)	364 (95)	974 (98)	862 (98)	1051 (99)	320 (93)
**Living with a partner N (%)**	2518 (72)	4702 (93)	352 (92)	898 (91)	852 (97)	1001 (95)	303 (88)
**Consumed alcohol during pregnancy N (%)**	266 (8)	0 (0)	0 (0)	0 (0)	0 (0)	0 (0)	0 (0)
**Total number of household members** Mean (sd)	3 (1)	5 (3)	5 (3)	5 (2)	6 (3)	5 (2)	5 (3)

†All four parents of the woman & her partner South Asian (SA) born

For peer review only - http://bmjopen.bmj.com/site/about/guidelines.xhtml

Table 2 Unadjusted and adjusted* odds ratios (95% CI) for socioeconomic characteristics for ethnic and generation groups

	**White British N=3503**	**All Pakistani births N=3656**	**Pakistani sub-groups defined by place of birth of parents**				
			**Pakistani: Woman & partner UK born† N=383**	**Pakistani: Woman UK born, partner SA born† N=992**	**Pakistani: Partner UK born, woman SA born† N=876**	**Pakistani: Woman &partner SA born† N=1060**	**Pakistani: Other N=345**
**In employment**							
Number (%)	**2272 (65)**	**881 (24)**	175 (46)	388 (39)	81 (9)	132 (12)	105 (30)
Unadjusted OR	**1**	**0.17 (0.16, 0.19)**	0.46 (0.37, 0.56)	0.35 (0.30, 0.40)	0.06 0.04, 0.07)	0.08 (0.06, 0.09)	0.24 (0.19, 0.30)
Adjusted OR*	**1**	**0.17 (0.15, 0.19)**	0.40 (0.32, 0.51)	0.38 (0.32, 0.44)	0.06 (0.04, 0.07)	0.06 (0.05, 0.08)	0.25 (0.19, 0.32)
**Educated post 16**							
Number (%)	**1601 (46)**	**1578 (43)**	235 (1)	473 (48)	306 (35)	401 (38)	163 (47)
Unadjusted OR	1	**0.90 (0.82, 0.99)**	1.89 (1.52, 2.34)	1.08 (0.94, 1.25)	0.64 (0.55, 0.74)	0.72 (0.63, 0.83)	1.06 (0.85, 1.33)
Adjusted OR*	1	**1.15 (1.04, 1.27)**	2.14 (1.70, 2.68)	1.37 (1.18, 1.59)	0.86 (0.73, 1.02)	0.88 (0.75, 1.03)	1.39 (1.11, 1.76)
**In receipt of means tested benefits****							
Number (%)	**1334 (38)**	**1742 (48)**	163 (43)	534 (54)	387 (44)	523 (49)	135 (39)
Unadjusted OR	**1**	**1.48 (1.35, 1.63)**	1.20 (0.97, 1.49)	1.90 (1.64, 2.19)	1.29 (1.11, 1.49)	1.58 (1.38, 1.89)	1.05 (0.83, 1.31)
Adjusted OR*	**1**	**0.97 (0.87, 1.09)**	1.02 (0.79, 1.30)	1.42 (1.20, 1.67)	0.71 (0.60, 0.84)	0.91 (0.78, 1.08)	0.84 (0.65, 1.09)
**Housing tenure: owns/part-owns (mortgage)**							
Number (%)	**1875 (54)**	**2600 (71)**	283 (74)	730 (74)	669 (76)	698 (66)	220 (64)
Unadjusted OR	**1**	**2.14 (1.94, 2.36)**	2.46 (1.94, 3.12)	2.42 (2.07, 2.83)	2.81 (2.37, 3.32)	1.67 (1.45, 1.93)	1.53 (1.21, 1.92)
Adjusted OR*	**1**	**2.30 (2.07, 2.56)**	2.49 (1.95, 3.18)	2.60 (2.20, 3.06)	3.35 (2.80, 3.99)	1.55 (1.32, 1.80)	2.02 (1.60, 2.57)

†All four parents of the woman & her partner South Asian (SA) born

*Adjusted for maternal age; parity

****** Any of: Income Support; Job Seekers Allowance; Working Tax Credit; Housing Benefits

For peer review only - http://bmjopen.bmj.com/site/about/guidelines.xhtml

Table 3 Unadjusted and adjusted* mean difference / odds ratios (95% CI) for lifestyle characteristics for ethnic and generation groups

	**White British N=3503**	**All Pakistani births N=3656**	**Pakistani sub-groups defined by place of birth of parents**				
			**Pakistani: Woman & partner UK born† N=383**	**Pakistani: Woman UK born, partner SA born† N=992**	**Pakistani: Partner UK born, woman SA born† N=876**	**Pakistani: Woman & partner SA born† N=1060**	**Pakistani: Other N=345**
**BMI at start of pregnancy** Mean (sd)	**26.8 (5.9)**	**25.7 (5.4)**	24.3 (4.6)	26.7 (5.7)	24.4 (4.7)	26.4 (5.6)	25.4 (5.3)
Unadjusted mean difference	**0**	**-1.15 (-1.41, -0.88)**	-2.53 (-3.13, -1.94)	-0.15 (-0.55, 0.25)	-2.44 (-2.86, -2.02)	-0.43 (-0.82, -0.04)	-1.40 (-2.02, -0.77)
Adjusted mean difference: Model 1*	**0**	**-1.75 (-2.01, -1.49)**	-2.84 (-3.41, -2.26)	-0.73 (-1.12, -0.34)	-2.95 (-3.36, -2.54)	-1.49 (-1.88, -1.10)	-1.22 (-1.83, -0.62)
Adjusted mean difference: Model 2**	**0**	**-1.12 (-1.43, -0.81)**	-2.32 (-2.92, -1.72)	-0.35 (-0.76, 0.07)	-2.22 (-2.69, -1.75)	-0.99 (-1.43, -0.57)	-0.77 (-1.39, -0.15)
**Smoked during pregnancy** Number (%)	**1183 (34)**	123 (3)	25 (7)	47 (5)	7 (0.8)	18 (2)	26 (8)
Unadjusted OR	**1**	**0.07 (0.06, 0.08)**	0.14 (0.09, 0.21)	0.09 (0.07, 0.13)	0.02 (0.01, 0.03)	0.03 (0.02, 0.05)	0.16 (0.11, 0.24)
Adjusted OR: Model 1*	**1**	**0.06 (0.05, 0.07)**	0.13 (0.09, 0.20)	0.09 (0.06, 0.12)	0.01 (0.01, 0.03)	0.03 (0.02, 0.05)	0.12 (0.08, 0.19)
Adjusted OR: Model 2**	**1**	**0.06 (0.05, 0.08)**	0.15 (0.09, 0.23)	0.09 (0.07, 0.13)	0.01 (0.01, 0.03)	0.03 (0.02, 0.05)	0.13 (0.08, 0.19)

†All four parents of the woman & her partner South Asian (SA) born

*Adjusted for maternal age; parity
**Adjusted for maternal age; parity; employment status; level of education, receipt of means tested benefits; housing tenure

For peer review only - http://bmjopen.bmj.com/site/about/guidelines.xhtml

Table 4 Unadjusted and adjusted* mean difference / odds ratios (95% CI) for health related pregnancy characteristics for ethnic and generation groups

	White British N=3503	All Pakistani births N=3656	Pakistani sub-groups defined by place of birth of parents				
			Pakistani: Woman & partner UK born† N=383	Pakistani: Woman UK born, partner SA born† N=992	Pakistani: Partner UK born, woman SA born† N=876	Pakistani: Woman & partner SA born† N=1060	Pakistani: Other N=345
**Hypertensive disorders of pregnancy** Number (%)	**239 (7)**	**188 (5)**	16 (4)	51 (5)	43 (5)	66 (6)	12 (3)
Unadjusted OR	**1**	**0.74 (0.61, 0.90)**	0.59 (0.35, 0.99)	0.74 (0.54, 1.01)	0.70 (0.51, 0.98)	0.91 (0.68, 1.20)	0.49 (0.27, 0.89)
Adjusted OR: Model 1*	**1**	**0.82 (0.67, 1.01)**	0.62 (0.37, 1.04)	0.82 (0.59, 1.12)	0.85 (0.61, 1.19)	0.99 (0.74, 1.33)	0.56 (0.31, 1.01)
Adjusted OR: Model 2**	**1**	**0.82 (0.64, 1.04)**	0.62 (0.36, 1.06)	0.81 (0.58, 1.13)	0.87 (0.59, 1.29)	1.01 (0.73, 1.40)	0.56 (0.31, 1.03)
Adjusted OR: Model 3***	**1**	**0.87 (0.67, 1.13)**	0.78 (0.45, 1.35)	0.80 (0.56, 1.14)	1.06 (0.70, 1.61)	1.06 (0.75, 1.49)	0.57 (0.31, 1.06)
**Gestational diabetes** Number (%)	**172 (5)**	406 (11)	30 (8)	96 (10)	92 (11)	159 (15)	29 (8)
Unadjusted OR	**1**	2.42 (2.01, 2.91)	1.65 (1.09, 2.46)	2.07 (1.59, 2.69)	2.27 (1.74, 2.96)	3.42 (2.72, 4.29)	1.78 (1.18, 2.68)
Adjusted OR: Model 1*	**1**	2.41 (1.98, 2.94)	1.66 (1.10, 2.49)	2.07 (1.58, 2.71)	2.54 (1.92, 3.35)	3.01 (2.36, 3.83)	2.24 (1.47, 3.41)
Adjusted OR: Model 2**	**1**	2.28 (1.82, 2.86)	1.66 (1.09, 2.53)	1.98 (1.49, 2.64)	2.47 (1.79, 3.39)	2.89 (2.20, 3.82)	2.21 (1.44, 3.40)
Adjusted OR: Model 3***	**1**	2.38 (1.86, 3.03)	1.89 (1.23, 2.92)	1.98 (1.46, 2.67)	2.82 (2.01, 3.97)	3.04 (2.27, 4.08)	2.29 (1.47, 3.56)

For peer review only - http://bmjopen.bmj.com/site/about/guidelines.xhtml

**Fasting glucose** Mean (sd)	**4.41 (0.41)**	**4.62 (0.62)**	4.54 (0.47)	4.58 (0.64)	4.54 (0.48)	4.73 (0.76)	4.60 (0.53)
Unadjusted mean difference	**0**	**0.20 (0.18, 0.23)**	0.13 (0.08, 0.19)	0.17 (0.14, 0.21)	0.13 (0.09, 0.17)	0.32 (0.29, 0.36)	0.19 (0.13, 0.25)
Adjusted mean difference: Model 1*	**0**	**0.18 (0.16, 0.21)**	0.12 (0.06, 0.17)	0.15 (0.11, 0.19)	0.12 (0.09, 0.16)	0.27 (0.24, 0.31)	0.22 (0.16, 0.27)
Adjusted mean difference: Model 2**	**0**	**0.18 (0.15, 0.21)**	0.12 (0.06, 0.18)	0.15 (0.11, 0.19)	0.12 (0.07, 0.16)	0.27 (0.23, 0.31)	0.22 (0.16, 0.27)
Adjusted mean difference: Model 3***	**0**	**0.20 (0.17, 0.24)**	0.17 (0.12, 0.23)	0.16 (0.12, 0.19)	0.17 (0.12, 0.21)	0.29 (0.25, 0.33)	0.23 (0.17, 0.29)
**Postload glucose** Mean (sd)	**5.47 (1.30)**	**5.89 (1.68)**	5.59 (1.35)	5.81 (1.58)	5.82 (1.50)	6.12 (2.02)	5.73 (1.45)
Unadjusted mean difference	**0**	**0.42 (0.35, 0.49)**	0.12 (-0.04, 0.28)	0.34 (0.23, 0.45)	0.35 (0.24, 0.46)	0.72 (0.62, 0.83)	0.26 (0.09, 0.42)
Adjusted mean difference: Model 1*	**0**	**0.37 (0.29, 0.44)**	0.08 (-0.07, 0.24)	0.29 (0.18, 0.39)	0.35 (0.24, 0.46)	0.58 (0.48, 0.69)	0.35 (0.19, 0.51)
Adjusted mean difference: Model 2**	**0**	**0.35 (0.27, 0.43)**	0.10 (-0.06, 0.26)	0.28 (0.17, 0.39)	0.33 (0.20, 0.46)	0.56 (0.44, 0.68)	0.34 (0.18, 0.51)
Adjusted mean difference: Model 3***	**0**	**0.37 (0.28, 0.45)**	0.18 (0.02, 0.34)	0.27 (0.16, 0.38)	0.39 (0.26, 0.52)	0.57 (0.45, 0.69)	0.35 (0.18, 0.52)
**Fasting insulin** Mean (sd)	**81.40 (46.72)**	**100.28 (62.76)**	92.66 (65.59)	100.76 (56.46)	91.75 (49.04)	106.11 (68.84)	111.09 (81.89)
Unadjusted mean difference	**0**	**18.88 (16.31, 21.45)**	11.26 (5.42, 17.09)	19.36 (15.46, 23.26)	10.36 (6.26, 14.45)	24.71 (20.91, 28.51)	29.69 (23.58, 35.81)
Adjusted mean difference: Model 1*	**0**	**18.08 (15.42, 20.74)**	10.98 (5.13, 16.82)	18.59 (14.64, 22.54)	9.67 (5.51, 13.83)	23.36 (19.43, 27.30)	29.69 (23.55, 35.82)
Adjusted mean difference: Model 2**	**0**	**21.29 (18.13, 24.45)**	14.01 (7.95, 20.08)	20.62 (16.40, 24.83)	13.53 (8.73, 18.34)	25.24 (20.89, 29.59)	32.01 (25.72, 38.31)
Adjusted mean difference: Model 3***	**0**	**25.71 (22.73, 28.69)**	24.44 (19.03, 29.86)	21.29 (17.47, 25.13)	23.27 (18.86, 27.68)	29.03 (25.04, 33.02)	34.79 (29.18, 40.39)

[†]All four parents of the woman & her partner South Asian (SA) born
*Adjusted for maternal age; parity
**Adjusted for maternal age; parity; employment status; level of education, receipt of means tested benefits; housing tenure
*** Adjusted for maternal age; parity; employment status; level of education, receipt of means tested benefits; housing tenure; early pregnancy BMI; smoking in pregnancy

For peer review only - http://bmjopen.bmj.com/site/about/guidelines.xhtml

Differences in socioeconomic position, lifestyle and health related pregnancy characteristics between Pakistani and White British women in the Born in Bradford prospective cohort study: the influence of the woman's, her partner's and their parents' place of birth.

West J *Specialty Registrar in Public Health*[1], **Lawlor DA** *professor of epidemiology* [2,3], **Fairley L** *statistician*[1], **Wright J** *professor of clinical epidemiology*[1]

[1] Bradford Institute for Health Research, Bradford Royal Infirmary, Bradford, BD9 6RJ, UK
[2] MRC Integrative Epidemiology Unit at the University of Bristol, UK
[3]School of Social and Community Medicine, University of Bristol, UK

Correspondence to jane.west@bthft.nhs.uk

For peer review only

ABSTRACT

Objective

To examine differences between Pakistani and White British women in relation to socioeconomic position, lifestyle and health related pregnancy characteristics, and to determine whether these differences vary depending on the woman's, her partner's and both of their parents' place of birth.

Design

Prospective cohort study.

Setting

Bradford, UK

Participants

3656 Pakistani and 3503 White British women recruited to the Born in Bradford study.

Main outcome measures

Socioeconomic position (employment status; level of education; receipt of benefits; housing tenure), lifestyle characteristics (BMI at the start of pregnancy; smoking during pregnancy) and health related pregnancy characteristics (hypertensive disorders of pregnancy; gestational diabetes; fasting glucose, postload glucose and fasting insulin at ~27 weeks gestation).

Results

Fewer Pakistani women were employed (OR 0.17 95% CI 0.15, 0.19), the difference being markedly less for UK born women. UK born Pakistani women were more likely, and South Asian born less likely, to be educated post 16 than White British women. Smoking was uncommon among Pakistani women, though the difference comparing UK born Pakistani women to White British women was less than for other groups. BMI was lower among Pakistani compared to White British women (adjusted mean difference -1.12 95% CI -1.43, -0.81) the difference greatest when partners were UK born irrespective of the woman's place of birth. Pakistani women had higher fasting and postload glucose (mean difference 0.20 mmol/l 95% CI 0.17, 0.24; 0.37 95% CI 0.28, 0.45), higher fasting insulin and were more likely to have gestational diabetes.

For peer review only - http://bmjopen.bmj.com/site/about/guidelines.xhtml

Conclusions

Our results suggest some socioeconomic, lifestyle and pregnancy characteristics could be beginning to change in response to migration to the UK, with generally beneficial changes i.e. improving education and employment prospects, lower BMI and no evidence that being UK born has further increased the risk of GDM, but some negative i.e. slight increases in smoking.

Strengths & limitations of this study

The strengths of this study include a large sample size, range of outcomes including oral glucose tolerance test data and detailed ancestry information.

We have for the first time, been able to examine ethnic differences in socioeconomic, lifestyle and pregnancy characteristics using information on the place of birth of women and their partners. We had also set out to explore differences based on all four grandparents but once we began analysing data it was apparent that for the majority of Pakistani women and their partners, all four of their parents were South Asian born. This limited our ability to explore differences across two generations, but highlights the persistence of strong family links in this community that have lived in Bradford for over 6 decades.

A potential limitation is that our results may not be generalizable to other South Asian populations and further work will be important to track these differences over future generations of UK South Asian migrants.

For peer review only

INTRODUCTION

Migration of South Asian populations to high income countries is generally thought to offer socioeconomic advantages in the form of improved education and employment opportunities, better housing and access to health care. However, improvements in environmental circumstances do not necessarily translate into improvements in health outcomes. Indeed, South Asian migrant populations to the UK experience an increased risk of maternal[1] and infant mortality[2] and some chronic diseases[3] compared with the UK population as a whole. This may reflect previous disadvantage associated with the country of origin which could persist over several generations, or could be a consequence of poor socioeconomic status within the host country. For example, UK South Asian communities are on average very poor[4]. That is, it could be that in comparison to those who do not migrate, there are improved health outcomes, but these remain poorer in comparison to the indigenous population. A further explanation is that the adoption of the unhealthy and sedentary lifestyles associated with acculturation or Westernisation, often characterised by low levels of physical activity[5], consumption of high calorie energy rich diets[6] and cigarette smoking[7,8], counteracts any potential health advantage of living in a higher income country. This may vary across different migrant communities but where this is the case, adoption of such lifestyles may be particularly harmful to South Asian individuals who for a given body mass index (BMI), have greater total and central adiposity and are known to be at greater risk of type 2 diabetes and cardiovascular disease than European adults[8-11].

Ethnic differences in socioeconomic position and lifestyle that might impact health during pregnancy could contribute to some of the known ethnic differences in pregnancy complications and perinatal outcomes. For example, they could contribute to the established greater risk of gestational diabetes (GDM)[12,13] and small for gestational age (SGA)[14-16] in South Asian compared to White British women. They could also drive ethnic differences in future generations either through intrauterine effects of maternal behaviours on these or as a result of the adoption of parental lifestyles by offspring and a lack of social migration. Previous studies have reported ethnic differences in socioeconomic and lifestyle characteristics between South Asian and White British women during pregnancy. Findings from the Millennium Cohort Study suggest South Asian women, in particular those originating from Pakistan and Bangladesh, are less likely to have formal educational qualifications, more likely to belong to lower socioeconomic groups and more likely to have never worked or be long term unemployed[7,16]. Marked differences in smoking and alcohol consumption between South Asian and White British women have also been reported[7,17]. Whilst outside pregnancy BMI is reportedly higher among South Asian women compared to White British women[18], we have previously reported that BMI is lower among Pakistani origin

For peer review only - http://bmjopen.bmj.com/site/about/guidelines.xhtml

pregnant women in the Born in Bradford (BiB) cohort[17]. Much less is known about maternal blood glucose and insulin in particular whether there are differences in these outcomes across generations of UK South Asian migrants. To our knowledge, no previous studies have examined ethnic differences in all these characteristics (socioeconomic, lifestyle, pregnancy) collectively which is important to identify areas where South Asian women may have better outcomes and those where European women may have better outcomes. This knowledge could support the delivery of appropriate antenatal care aimed at maximising maternal and child health in both White British and South Asian groups.

Furthermore, previous studies have not explored whether any identified ethnic differences during pregnancy are consistent when the mother's, her partner's and both of their parents' country of origin are taken into account. In a previous study, using data from the Born in Bradford cohort, which is used in this paper, we showed that birthweight was lower, but that birth fatness (assessed using skinfold thickness and cord blood leptin) was greater in Pakistani compared to White British infants[17]. We further showed that these differences did not differ by whether both the mother and her partner and all four of their parents were born in the UK, all born in South Asia or there was a mixed pattern between these two extremes[17]. Here, we extend that work to look at a range of socioeconomic position, lifestyle and pregnancy related outcomes, in order to understand whether in the context of place of birth of women and her closest family relatives, there are some ethnic differences that are reduced or some that are enhanced, and if so whether these would be beneficial or detrimental to health.

The aim of this study was to examine differences between Pakistani women and White British women in relation to socioeconomic position (employment status; level of education; receipt of means tested benefits; housing tenure), lifestyle characteristics (BMI at the start of pregnancy; smoking during pregnancy) and health related pregnancy characteristics (hypertensive disorders of pregnancy (HDP); gestational diabetes; fasting glucose, postload glucose and fasting insulin at ~27 weeks gestation), and to determine whether these differences vary depending upon the woman's, her partner's and both of their parents' place of birth.

METHODS

Participants

The Born in Bradford (BiB) study is a largely bi-ethnic prospective birth cohort study that recruited women during pregnancy and has followed them, their infants and their partners into the child's infancy. To be eligible for the study women had to attend booking clinic

For peer review only - http://bmjopen.bmj.com/site/about/guidelines.xhtml

between March 2007 and December 2010 and be booked to give birth in the city of Bradford. Full details of the study methodology have been previously reported[18]. Women were recruited at their oral glucose tolerance test (OGTT) appointment; all women booked for delivery in Bradford are offered a 75g OGTT (comprising fasting and 2 hour postload samples) at around 26 – 28 weeks gestation. Women who attended this appointment and agreed to take part in the study consented to the use of their obstetric medical records, had their height and weight recorded and completed an interviewer administered questionnaire. The questionnaire included questions relating to ethnicity, social and economic circumstances, smoking, alcohol, diet, education and employment and collected place of birth information for both parents and all four grandparents. Interviews were conducted in a range of South Asian languages (including Mirpuri, Bengali, Punjabi). Mirpuri is the most commonly spoken Asian language in Bradford but has no written script therefore questionnaires were transliterated, that is translated verbally to Mirpuri and then written phonetically, precisely as spoken to ensure that all interpreters translated it in the same way. Details of the language used to conduct the questionnaire were recorded. Ethics approval for the study was provided by Bradford Local Research Ethics Committee (ref 06/Q1202/48). Data were available for 11,113 women recruited to the BiB cohort. We excluded stillbirths (n=64) and infants born to parents of ethnic origin other than White British or Pakistani (n=1598). Of the remaining 9451 participants 7159 had complete data for all variables included in all models thus 3656 Pakistani and 3503 White British women are included in these analyses. Women with existing diabetes (0.5% of the BiB cohort) are not invited to attend for the glucose tolerance test as they are treated from the start of their pregnancy by an endocrine physician. As a result these women were not recruited at the same time as other participants and do not have some data, including parental place of birth. These women are therefore not included in these complete case analyses.

Woman's family member's place of birth

Ethnicity was self-reported at interview, with participants given response options based on UK Office of National Statistics guidance[19]. Women completed a detailed ancestry interview, which included details of the place of birth of themselves, their partner and all four parents of themselves and their partner. Family place of birth groups of the Pakistani infants were derived from these data as previously reported[17]. In the previous report, since our outcome of interest was infant birth size the groups were defined in terms of 'parents' and 'grandparents'. As our outcomes here are in pregnant women we have described them in relation to her, but the groups are essentially the same as the previous paper. Our aim in that previous paper, as here, was to examine differences across all possible groups based on place of birth of the woman, her partner and all four parents. Thus, we began by

For peer review only - http://bmjopen.bmj.com/site/about/guidelines.xhtml

determining numbers in all 64 possible combinations of these six family members. Having done that it was apparent that for almost all women, the four parents of the woman and her partner were South Asian born meaning that the analyses were based primarily on the woman's and her partner's place of birth. Overall, 90% of women fell into one of four main categories:

1. Woman and her partner UK born and all four of their parents South Asian born
2. Woman UK born, partner and all four of their parents South Asian born
3. Partner UK born, woman and all four parents South Asian born
4. Woman, her partner and four parents all South Asian born

The remaining 11% (n=345), including those with one or more of the woman's or her partner's parents being UK born or where their parents' place of birth was unknown, was combined to form one 'other' group.

Outcome measures

Socioeconomic

Information on socioeconomic indicators (employment, education, receipt of benefits, housing tenure) was obtained from the interview with the woman at recruitment. We equivalised the mother's highest educational qualifications (based on the qualification received and the country obtained) into one of several categories using UK NARIC (http://www.ecctis.co.uk/naric/default.aspx): *<5 GCSE equivalent, ≥5 GCSE equivalent, 'A' level equivalent, Higher than A-level equivalent, Other qualifications (e.g. City and Guilds, RSA/OCR, BTEC), Don't know, Foreign Unknown.* Don't know relates to the mother responding "don't know" during interview. Foreign Unknown relates to a qualification listed in the free text response but no level of qualification is given or the qualification listed cannot be equivalised to one of the above categories. For these analyses, women were categorised as having been educated beyond the age of 16 or not (i.e. *Higher than A-level equivalent, Other qualifications (e.g. City and Guilds, RSA/OCR, BTEC).* Receipt of means tested benefits was based on the mother or her household receiving any of: Income Support, Job Seekers Allowance, Working Tax Credit or Housing Benefit. Housing tenure was categorised according to whether the woman lived in a household where the home was either part-owned (i.e. mortgaged) or owned outright, or not (i.e. rented).

Lifestyle

BMI is used in these analyses as a proxy marker of lifestyle as it is an outcome that can potentially be influenced by changes or differences in lifestyle (in particular dietary choices

For peer review only - http://bmjopen.bmj.com/site/about/guidelines.xhtml

and levels of physical activity). At recruitment, women were weighed and their height measured (unshod and in light clothing) using SECA digital scales and a Leicester Height Measure respectively. Weight at first antenatal clinic assessment when women were around 12 weeks gestation (median 12 weeks, IQR 11, 14), was abstracted from the antenatal records and this weight together with height measured at recruitment, was used to calculate the woman's BMI so that this reflected early pregnancy BMI before substantial contribution from pregnancy and the growing fetus. Information on smoking was obtained at the questionnaire interview, with women categorised as having smoked cigarettes at any stage of their pregnancy or not. As none of the Pakistani origin women reported drinking alcohol, we were unable to include alcohol consumption as an outcome.

Health related pregnancy characteristics

Women were classified as hypertensive in pregnancy if they had a systolic measure ≥140 and a diastolic ≥90mmHg on 2 or more occasions after 20 weeks gestation; information on this was obtained from the antenatal records. Fasting and postload glucose and fasting insulin were obtained from the OGTT plasma samples which were assayed immediately after sampling at the biochemistry department of Bradford Royal Infirmary using the glucose oxidase method on Siemen's Advia 2400 chemistry autoanalysers. GDM was defined using the fasting and postload glucose according to WHO criteria[20] at the time these women were pregnant as either a fasting glucose ≥6.1mmol/L or a two-hour postload glucose ≥7.8mmol/L. Women with existing diabetes prior to pregnancy did not complete an OGTT and are not included in this sample.

Statistical analyses

All analyses were performed using Stata (version 12.1). We used univariable regression to examine the association of ethnicity and family place of birth group with outcomes. Included predictor variables were decided a priori based on existing evidence and knowledge. Logistic regression was used for binary outcomes and linear regression for continuous outcomes, with the White British group used as the reference for all analyses, i.e. we compared outcomes in 'all' Pakistani women and then each of the five family place of birth subgroups of Pakistani women to outcomes in White British women. The rationale for this is because our aim is primarily to compare all Pakistani origin women with White British women and then to compare subgroups based on place of birth with the same reference group of White British women to see if place of birth of the Pakistani women influences the extent to which they differ or not from the indigenous population. In all adjusted analyses we adjusted for maternal age and parity (Model 1). For the lifestyle outcomes (early pregnancy BMI; smoking) we also adjusted for each of the indicators of socioeconomic position in order

For peer review only - http://bmjopen.bmj.com/site/about/guidelines.xhtml

to explore the extent of any differences in these lifestyles might reflect ethnic differences in socioeconomic position (Model 2). For the health related pregnancy characteristics we also adjusted for socioeconomic indicators (Model 2) and also for the lifestyle characteristics (BMI; smoking) (Model 3), to explore whether these explained any of the differences. When age and BMI were included in models as covariables they were used as continuous variables. Existing literature supports their linear associations with outcomes and we confirmed this graphically. For all multivariable models we examined the residuals and these were all found to be approximately normal. Further, we checked potential problems with collinearity in each model by assessing variance inflation and found that this was lower than 2 for all independent variables in all models.

RESULTS

The characteristics of White British and Pakistani origin women are shown in Table 1. There was little difference between the two ethnic groups in mean gestation, premature births and infant sex. As reported in our previous paper[17], birthweight of their infant was markedly lower in Pakistani compared to White British women when all Pakistani origin women were combined and also when compared by subgroups based on place of birth. On average, Pakistani origin women were slightly older, in particular when both parents were South Asian born, markedly more likely to be married and lived within larger households than White British women. These differences were similar across all generation groups. Pakistani women were shorter than White British women but the difference was less when women were UK born. There were also some differences in parity across Pakistani generation groups, for example, parity was on average lowest when both parents were UK born and highest when both parents were born in South Asia.

The odds of being in employment for Pakistani women were 83% lower than White British women (adjusted OR 0.17 95% CI 0.15, 0.19), but there were differences by family place of birth (Table 2). These odds were 94% less for those who were South Asian born but this difference reduced to 60% for Pakistani women when both they and their partner were UK born. Following adjustment for maternal age and parity, Pakistani women as a whole were more likely to be educated beyond the age of 16 than White British women (OR 1.15 95% CI 1.04, 1.27), however there were marked differences across family place of birth groups with women who were South Asian born being less likely, and those who were UK born being more likely compared to White British women, to be educated beyond 16 years. Being in receipt of means tested benefits was similar in both ethnic groups when Pakistani women were assessed as a whole (adjusted OR 0.97 95% CI 0.87, 1.09) although for Pakistani women who were UK born with a South Asian partner there were increased odds of

For peer review only - http://bmjopen.bmj.com/site/about/guidelines.xhtml

receiving benefits. Compared to White British women, Pakistani women were considerably more likely to own or part own their home (adjusted OR 2.30 95% CI 2.07, 2.56) and this was consistent across all family place of birth groups.

Table 3 shows the unadjusted and adjusted (Models 1 and 2) ethnic difference in lifestyle characteristics. Pakistani women had a lower BMI than White British women (adjusted [Model 2] mean difference -1.12 95% CI -1.43, -0.81) but the difference was much greater when the woman's partner was UK born irrespective of where the woman herself was born (Figure 1). The odds of smoking for Pakistani women were around 94% less and this was similar across generation groups other than when both the woman and her partner were UK born in which case they were 85% less. None of the Pakistani women reported drinking any alcohol during pregnancy (0%), whereas 8% of White British women drank during pregnancy.

In Table 4 the unadjusted and adjusted (Models 1-3) ethnic difference in pregnancy characteristics is shown. Fewer Pakistani women in general had HDP (adjusted [Model 3] OR 0.87 95% CI 0.67, 1.13), although this result was imprecisely estimated with wide confidence intervals that included the null. This was not consistent across all family place of birth groups for example, women who were South Asian born were slightly more likely to have HDP than White British women and this was the case in all 3 adjusted models. Pakistani women were more likely to have GDM and higher fasting and postload glucose and fasting insulin than White British women and these differences were broadly similar across all 3 models of adjustment. There were some differences by family place of birth group, for example, the difference in postload glucose between Pakistani and White British women was far greater when the woman and her partner were born in South Asia than when both were UK born (adjusted mean difference [Model 3] 0.57 95% CI 0.45, 0.69 and 0.18 95% CI 0.02, 0.34 respectively and Figure 2).

DISCUSSION

We have shown differences across a range of socioeconomic, lifestyle and pregnancy characteristics between Pakistani and White British origin women and that these vary depending on whether Pakistani women are born in the UK or South Asia. We have for the first time, been able to consider not only the woman's place of birth, but also her partner's and both of their parents' place of birth; though after preliminary analyses it was clear that for the majority of women and their partners, all four of their parents were South Asian born. This provides important information about how these differences might be reduced or even enhanced with greater acculturation over generations. For example, the odds of Pakistani

For peer review only - http://bmjopen.bmj.com/site/about/guidelines.xhtml

women as a whole being in employment, were 83% less than White British women, but across generation groups this difference varied from 60% when both the woman and her partner were born in the UK, to 94% when both the woman and her partner were South Asian born. Likewise, we found interesting differences in education attainment between Pakistani and White British women. Overall, Pakistani women were slightly more likely to have been educated beyond the age of 16, but this was driven by UK born Pakistani women, especially those with a UK born partner who were twice as likely as White British women to have completed education beyond age 16. By contrast, South Asian born Pakistani women, irrespective of their partner's place of birth, were less likely than White British women to have been educated beyond the age of 16. This could reflect a positive effect of migration and acculturation on social mobility which likely plays a part in the employment differences described above and is consistent with previous reports[7,21]. Whilst differences in employment and education by place of birth suggest the adoption of some British lifestyle characteristics, the tendency of Pakistani women to live within larger households and to be more likely to own or part-own their own home, suggests that the traditional culture of living within extended families has been maintained across all place of birth sub-groups of Pakistani women.. Living with an extended family could have considerable benefits for the mother and her offspring, such as childcare support and greater social capital, but could also result in overcrowding and potential detrimental impacts of this on health[22]. Early analyses using data from BiB suggests that living with more family members does not lead to greater family social capital (Cabieses B, unpublished data 2013). Pakistani women who were born in the UK but had a South Asian born partner, were more likely to claim benefits compared to White British women than those who were South Asian born which is surprising given that they tend to be more likely to be in employment. This might reflect a tendency for South Asian born partners to be in lower paid employment reducing total household income, or that poorer command of the English language (likely amongst those Pakistani women who were South Asian born and were less likely to claim benefits compared to White British women) is a barrier to accessing services and social support.

Greater social migration, for example coming to the UK for social reasons, has been associated with increased uptake of lifestyle characteristics of the host country such as smoking and alcohol consumption[7]. We report a similar trend in that UK born Pakistani origin women were more likely to smoke than South Asian born women, but smoking was still uncommon among all Pakistani women compared to White British women and none of them reported any alcohol consumption during pregnancy. Thus, the increase in these harmful health behaviours over generations in some migrant groups, whilst showing some signs of change, appears minimal among Pakistani women. This may reflect persisting cultural or

For peer review only - http://bmjopen.bmj.com/site/about/guidelines.xhtml

religious influences[23,24] and could be related to the fact that for the majority of women, both of their parents and their partners parents were South Asian born We found BMI to be slightly lower among Pakistani origin women compared to White British women although there were interesting differences across family place of birth groups. The finding that the difference in BMI between Pakistani and White British women was markedly greater for Pakistani women with a UK born partner, irrespective of their own place of birth, than for women with a South Asian born partner is particularly striking. One possible explanation is that within this population, partners/husbands have a particularly dominant role[25]. Thus, the lifestyle choices of the family or household will be driven mostly by the social norms and habits of the partner. In the case of men born in the UK, these are likely to be influenced by western culture which promotes a lower BMI as both healthy and attractive. Similarly, having been brought up and educated in the UK, they may be more likely to participate in organised physical activity and also may be more receptive to UK public health campaigns.

Health related pregnancy characteristics may be the most important to the long-term health of South Asian migrants in the UK, particularly in relation to the association of these characteristics with cardiovascular disease and type 2 diabetes[26]. We report a number of differences between Pakistani women and White British women in HDP, glucose tolerance, fasting insulin and GDM. Pakistani women as a whole group were less likely to have HDP, although this was not consistent across family subgroups, but they were more than twice as likely to have GDM. Consistent with these higher rates of GDM, Pakistani women had higher fasting and postload glucose and higher fasting insulin than White British women. These findings are similar to those from previous studies showing that South Asian women are more likely to have GDM than White European women[12,13] . They are also consistent with considerable evidence that adult non-pregnant women and men have a higher risk of insulin resistance and type 2 diabetes[9-11,26]. We found that the increased likelihood of Pakistani women having GDM compared to White British women was greatest for South Asian born women. We also found that the mean difference in fasting and postload glucose and fasting insulin relative to White British women was substantively greater when the woman and her partner were both born in South Asia. This is somewhat surprising as evidence suggests that the increased risk of insulin resistance and type 2 diabetes in South Asian adults compared to White Europeans is largely amongst those in urban (rather than rural) areas of South Asia[27], or in those who have migrated to Western countries[9,28]. We might therefore have expected the increase to be greater amongst those who were UK born. The difference between our findings and these previous studies of non-pregnant migrants[,9,26,27], might be explained by differences in the population studied, with many of these previous studies being of Indian, or mixed rather than Pakistani origin. Pakistani

For peer review only - http://bmjopen.bmj.com/site/about/guidelines.xhtml

migrants in general tend to be poorer, shorter and weigh less, and the Pakistani women in this study have lower BMI than the White British women. For religious and cultural reasons Pakistani women remain unlikely to smoke or drink alcohol which could influence their glucose tolerance, although smoking is related to lower BMI and therefore would be expected to reduce glucose tolerance[29]. It might also be that whilst insulin resistance and diabetes in the general population are enhanced in those who migrate and particularly with greater duration of migration, in pregnancy the impact of place of birth or time since migration differs. We are not aware of other studies with equivalent data to explore this further, but it would be interesting to see if this finding is replicated.

The key strengths of this study are the large sample size, range of outcomes we have been able to examine, including OGTT data, and the detailed information on place of birth. To our knowledge this is the first study to examine differences between Pakistani and White British women in relation to socioeconomic, lifestyle and pregnancy characteristics using detailed information on the place of birth of women and their partners. We had hoped to explore three generations of Pakistani migrants to Bradford, but for almost all the women in this study, their parents and the parents of their partner were born in South Asia. However, this is in itself an interesting finding and useful for meeting future health needs in the city. It might also explain some of our findings in relation to the persistence of some characteristics across family place of birth subgroups. A potential limitation of our study was the inability to include other South Asian groups in our analyses (Indian and Bangladeshi) due to small numbers within our cohort. On the one hand examining a specific South Asian population (Pakistani) reduces the problem of heterogeneity between South Asian groups but at the same time it may limit the generalisability of our results to other South Asian populations. Our analyses have not accounted for South Asians who migrate to the UK in childhood and may be resident in the UK for much of their development and education, which could potentially dilute any differences between the Pakistani place of birth groups. Within BiB information regarding the age at which an individual migrated to the UK is only available for women (not their partner or parents) therefore we were not able to account for this in our family place of birth groups. We were not able to validate self-report of smoking or alcohol consumption in pregnancy for either the Pakistani or White British women. If reporting bias, which might occur because of the stigma associated with these behaviours in pregnancy, is similar in each ethnic group it should not bias the comparisons that are the main focus of this paper. Many of the researchers who collected interview data were of Pakistani origin and it is possible that this may have resulted in greater under-reporting in the Pakistani origin women. However, the prevalence of these behaviours in this study is similar to those in other studies of Pakistani women[7].

For peer review only - http://bmjopen.bmj.com/site/about/guidelines.xhtml

In summary, we have found some evidence that the difference in some of these characteristics between Pakistani and White British women may be changing in response to migration to the UK, in that differences were seen most often in those where the woman or her partner were UK born. Several of these differences would be beneficial to health and wellbeing. For example, Pakistani women born in the UK were more likely than White British women to be educated beyond age 16. UK born Pakistani women were also more similar to White British women in terms of employment and there was no evidence that being UK born increased their risk of GDM or glucose intolerance. On the other hand, whilst overall prevalence of smoking in Pakistani women in all groups was very small, the difference between them and White British women was least when they were UK born. We have also identified differences that vary according to the woman's partner's place of birth, for example BMI is lower among Pakistani women with a UK born partner. Further work is needed that continues to track these important ethnic differences over future generations to support the delivery of appropriate antenatal care.

Words: Abstract:300 ; Main text: 4800

Acknowledgements

Born in Bradford is only possible because of the enthusiasm and commitment of the children and parents in Born in Bradford. We are grateful to all participants, health professionals and researchers who have made Born in Bradford happen.

Funding

J West was funded by a UK Medical Research Council (MRC) Special Training Fellowship in Health of the Public and Health Services Research (MRC GO601712). DA Lawlor works in a unit that receives UK MRC funding (MC_UU_12013/5). BiB receives core infrastructure funding from the Wellcome Trust (WT101597MA).

Competing interests

All authors declare no competing interests

Author contributions

J West, DA Lawlor and J Wright conceived the study idea, obtained funds, developed the statistical analysis plan, were involved in managing the data collection and wrote the initial drafts of the paper; J West undertook the main analysis with input from L Fairley and supervision from DA Lawlor and J Wright. J West acts as guarantor.

For peer review only - http://bmjopen.bmj.com/site/about/guidelines.xhtml

Declaration of transparency

J West affirms that the manuscript is an honest, accurate, and transparent account of the study being reported; that no important aspects of the study have been omitted; and that any discrepancies from the study as planned (and, if relevant, registered) have been explained.

This is an Open Access article distributed in accordance with the Creative Commons Attribution Non Commercial (CC BY-NC 3.0) license, which permits others to distribute, remix, adapt, build upon this work non-commercially, and license their derivative works on different terms, provided the original work is properly cited and the use is non-commercial. See:http://creativecommons.org/licenses/by-nc/3.0/.

Data sharing

Scientists are encouraged and able to use BiB data. Data requests are made to the BiB executive using the form available from the study website www.borninbradford.nhs.uk (please click on "Science and Research" to access the form). Guidance for researchers and collaborators, the study protocol and the data collection schedule are all available via the website. All requests are carefully considered and accepted where possible.

Figure 1 Adjusted mean differences in BMI for Pakistani women relative to White British women

*Model 2: Adjusted for maternal age; parity; employment; post-16 education; receipt of means tested benefits; housing tenure

Figure 2 Adjusted mean differences in postload glucose for Pakistani women relative to White British women

**Model 3: Adjusted for maternal age; parity; employment; post-16 education; receipt of means tested benefits; housing tenure; early pregnancy BMI; smoking in pregnancy

References

1. Saving Mothers' Lives: Reviewing maternal deaths to make motherhood safer: 2006-2008. *BJOG* 2011 Mar 1;118 Suppl 1:1–203

2. Office for National Statistics (ONS). Gestation-specific infant mortality in England and Wales, 2010. London, ONS 2012. At http://www.ons.gov.uk/ons/dcp171778_282579.pdf accessed on 18/03/2014.

3. Bhala N, Zaman MS. Preventing premature mortality in chronic diseases for South Asians in the UK and beyond. *J R Soc Med* 2009;102(11): 459–463.

4. Nazroo, J. Ethnicity, class and health. London, Policy Studies Institute, 2001.

5. Williams ED, Stamatakis E, Chandola T et al. Assessment of physical activity levels in South Asians in the UK: findings from the Health Survey for England. *JECH* 2011;65:517-21.

6. Patel JV, Vyas A, Cruickshank JK et al. Impact of migration on coronary heart disease risk factors: comparison of Gujaratis in Britain and their contemporaries in villages of origin in India. *Atherosclerosis* 2006;185:297-306.

7. Hawkins SS, Lamb K, Cole TJ et al. Influence of moving to the UK on maternal health behaviours: prospective cohort study. *BMJ* 2008; 336:1052.

8. Sproston, K; Mindell, JE. Health Survey for England 2004. The health of minority ethnic groups. HSE 2006. Information Centre, London.
9. Barnett AH, Dixon AN, Bellary S, et al. Type 2 diabetes and cardiovascular risk in the UK south Asian community. *Diabetologia* 2006;49(10):2234-46.
10. Mather HM, Keen H. The Southall Diabetes survey: prevalence of known diabetes in Asians and Europeans. *BMJ* 1985;291:1081-4.

11. Gholap N, Davies M, Patel K et al. Type 2 diabetes and cardiovascular disease in South Asians. *Prim Care Diabetes* 2011;5(1):45-56. Epub 2010/09/28.

12. Dornhurst A, Pateson CM, Nicholls JS et al. High prevalence of gestational diabetes mellitus in women from ethnic minority groups. *Diabet Med* 1992; Nov;9(9):820-5

13. Makgoba M, Savvidou MD, Steer PJ. An analysis of the interrelationship between maternal age, body mass index and racial origin in the development of gestational diabetes mellitus. *BJOG* 2012; Feb;119(3):276-82.

14. Margetts BM, Mohd Yusof S, Al Dallal Z et al. Persistence of lower birth weight in second generation South Asian babies born in the United Kingdom. *J Epidemiol Community Health* 2002 Sep;56(9):684-7.

15. Leon DA, Moser K. Low birth weight persists in South Asian babies born in England & Wales regardless of maternal country of birth. Slow pace of acculteration, physiological constraint or both? Analysis of routine data. *J Epidemiol Community Health* 2010; Published online 30 Nov 2010. doi:10.1136/jech2010.112516

16. Kelly Y, Panico L, Bartlet M et al. Why does birthweight vary among ethnic groups in the UK? Findings from the Millennium Cohort Study. *J Public Health (Oxf)* 2008;31(1):131-137.

17. West J; Lawlor DA; Fairley L et al. UK born Pakistani origin infants are relatively more adipose than White British infants: findings from 8704 mother-offspring pairs in the Born in Bradford prospective birth cohort. *J Epidemiol Community Health* 2013; doi:10.1136/jech-2012-20189.1

18. WrightJ, Small N, Raynor P et al. Cohort profile: the Born in Bradford multi-ethnic family cohort study. *Int J Epidemiol* 2013; Aug;42(4):978-91.

19. Office for National Statistics (ONS). Ethnic group statistics: a guide for the collection and classification of ethnicity data. London: Stationary Office; 2003.

20. World Health Organisation. Definition, diagnosis and classification of diabetes mellitus and its complications. Report of a WHO consultation. Part 1: diagnosis and

For peer review only - http://bmjopen.bmj.com/site/about/guidelines.xhtml

classification of diabetes mellitus. WHO 1999 at http://whqlibdoc.who.int/hq/1999/who_ncd_ncs_99.2.pdf accessed on 18/03/2014.

21. Berthaud R. Family formation in multicultural Britain: diversity and change. In Ethnicity, social mobility and public policy, Loury GC, Modood T, Teles SM (Eds).Cambridge: Cambridge University Press; 2005.

22. Galobardes B, Shaw M, Lawlor DA et al. Indicators of socioeconomic position (Part 1). *J Epidemiol Community Health* 2006; 60(1):7-12.

23. Bradby H, Williams R. Is religion or culture the key feature in changes in substance use after leaving school? Young Punjabis and a comparison group in Glasgow. *Ethn and Health* 2006;11(3):307-324.

24. Hurcombe R, Bayley M, Goodman A. Ethnicity and alcohol. A review of the UK literature. Joseph Rowntree Foundation 2010.

25. Beishon S, Modood T, Virdee S. Ethnic minority families. London : Policy Studies Institute, 1998.

26. McKeigue PM, Pierpoint T, Ferrie JE et al. Relationship of glucose intolerance and hyperinsulinaemia to body fat pattern in South Asians and Europeans. *Diabetologia* 1992;35(8):785-91.

27. Ebrahim S, Kinra S, Bowen L et al. The effect of rural-to-urban migration on obesity and diabetes in India: a cross-sectional study. *PLoS Med* 2010 Apr 27;7(4).

28. Landman J, Cruickshank JK. A review of ethnicity, health and nutrition-related diseases in relation to migration in the United Kingdom. *Public Health Nutr* 2001;4 (2b):647-657.

29. Freathy RM, Kazeem GR, Morris RW et al. Genetic variation at *CHRNA5-CHRNA3-CHRNB4* interacts with smoking status to influence BMI. *Int J Epidemiol* 2011;40:1617-1628.

For peer review only - http://bmjopen.bmj.com/site/about/guidelines.xhtml

Table 1 Characteristics of women and infants (N=9450) by ethnic and generation group

	White British (UK & Ireland)	All Pakistani births	Pakistani sub-groups defined by place of birth of parents				
			Pakistani: Woman & partner UK born†	Pakistani: Woman UK born, partner SA born†	Pakistani: Partner UK born, woman SA born†	Pakistani: Woman & partner SA born†	Pakistani: Other
**Number**	3503	3656	383	992	876	1060	345
**Gestation at delivery (weeks)** Mean (sd)	39.0 (1.9)	39.0 (1.8)	39.0 (1.8)	38.9 (1.9)	39.0 (1.9)	39.1 (1.7)	39.1 (1.6)
**Births before 37 weeks N (%)**	209 (6.0)	204 (5.6)	22 (5.7)	63 (6.4)	50 (5.7)	52 (4.9)	17 (4.9)
**Mean birth weight in gm (sd)**	3346 (568)	3124(540)	3114 (538)	3100 (549)	3101 (537)	3160 (547)	3158 (497)
**Sex N (%)**							
**Male**	1808(52)	1851(51)	200(52)	504(51)	420(48)	535(51)	192(56)
**Female**	1695(48)	1805(49)	183(48)	488(49)	456(52)	525(49)	153(44)
**Maternal age** Mean (sd)	27 (6)	28 (5)	28 (5)	28 (5)	27 (5)	30 (5)	26 (5)
**Maternal height (m) Mean (sd)**	1.64 (0.06)	1.60 (0.06)	1.61 (0.05)	1.60 (0.06)	1.59 (0.05)	1.59 (0.05)	1.61 (0.06)
**Parity N (%)**							
0	1688 (48)	1157 (32)	155 (40)	331 (33)	253 (29)	254 (24)	164 (47)
1	1122 (32)	986 (26)	105 (27)	261 (26)	253 (29)	265 (25)	102 (30)
2	454 (13)	754 (21)	76 (20)	194 (20)	199 (23)	233 (22)	52 (15)
3	139 (4)	462 (13	34 (9)	125 (13)	111 (12)	178 (17)	14 (4)
4 or more	100 (3)	297 (8)	13 (4)	81 (8)	60 (7)	130 (12)	13 (4)
**Married N (%)**	1149 (33)	3571 (98)	364 (95)	974 (98)	862 (98)	1051 (99)	320 (93)
**Living with a partner N (%)**	2518 (72)	4702 (93)	352 (92)	898 (91)	852 (97)	1001 (95)	303 (88)
**Consumed alcohol during pregnancy N (%)**	266 (8)	0 (0)	0 (0)	0 (0)	0 (0)	0 (0)	0 (0)
**Total number of household members** Mean (sd)	3 (1)	5 (3)	5 (3)	5 (2)	6 (3)	5 (2)	5 (3)

†All four parents of the woman & her partner South Asian (SA) born

For peer review only - http://bmjopen.bmj.com/site/about/guidelines.xhtml

Table 2 Unadjusted and adjusted* odds ratios (95% CI) for socioeconomic characteristics for ethnic and generation groups

	White British N=3503	All Pakistani births N=3656	Pakistani sub-groups defined by place of birth of parents				
			Pakistani: Woman & partner UK born† N=383	Pakistani: Woman UK born, partner SA born† N=992	Pakistani: Partner UK born, woman SA born† N=876	Pakistani: Woman &partner SA born† N=1060	Pakistani: Other N=345
**In employment**							
Number (%)	**2272 (65)**	**881 (24)**	175 (46)	388 (39)	81 (9)	132 (12)	105 (30)
Unadjusted OR	**1**	**0.17 (0.16, 0.19)**	0.46 (0.37, 0.56)	0.35 (0.30, 0.40)	0.06 0.04, 0.07)	0.08 (0.06, 0.09)	0.24 (0.19, 0.30)
Adjusted OR*	**1**	**0.17 (0.15, 0.19)**	0.40 (0.32, 0.51)	0.38 (0.32, 0.44)	0.06 (0.04, 0.07)	0.06 (0.05, 0.08)	0.25 (0.19, 0.32)
**Educated post 16**							
Number (%)	**1601 (46)**	**1578 (43)**	235 (1)	473 (48)	306 (35)	401 (38)	163 (47)
Unadjusted OR	1	**0.90 (0.82, 0.99)**	1.89 (1.52, 2.34)	1.08 (0.94, 1.25)	0.64 (0.55, 0.74)	0.72 (0.63, 0.83)	1.06 (0.85, 1.33)
Adjusted OR*	1	**1.15 (1.04, 1.27)**	2.14 (1.70, 2.68)	1.37 (1.18, 1.59)	0.86 (0.73, 1.02)	0.88 (0.75, 1.03)	1.39 (1.11, 1.76)
**In receipt of means tested benefits****							
Number (%)	**1334 (38)**	**1742 (48)**	163 (43)	534 (54)	387 (44)	523 (49)	135 (39)
Unadjusted OR	**1**	**1.48 (1.35, 1.63)**	1.20 (0.97, 1.49)	1.90 (1.64, 2.19)	1.29 (1.11, 1.49)	1.58 (1.38, 1.89)	1.05 (0.83, 1.31)
Adjusted OR*	**1**	**0.97 (0.87, 1.09)**	1.02 (0.79, 1.30)	1.42 (1.20, 1.67)	0.71 (0.60, 0.84)	0.91 (0.78, 1.08)	0.84 (0.65, 1.09)
**Housing tenure: owns/part-owns (mortgage)**							
Number (%)	**1875 (54)**	**2600 (71)**	283 (74)	730 (74)	669 (76)	698 (66)	220 (64)
Unadjusted OR	**1**	**2.14 (1.94, 2.36)**	2.46 (1.94, 3.12)	2.42 (2.07, 2.83)	2.81 (2.37, 3.32)	1.67 (1.45, 1.93)	1.53 (1.21, 1.92)
Adjusted OR*	**1**	**2.30 (2.07, 2.56)**	2.49 (1.95, 3.18)	2.60 (2.20, 3.06)	3.35 (2.80, 3.99)	1.55 (1.32, 1.80)	2.02 (1.60, 2.57)

†All four parents of the woman & her partner South Asian (SA) born

*Adjusted for maternal age; parity

****** Any of: Income Support; Job Seekers Allowance; Working Tax Credit; Housing Benefits

Table 3 Unadjusted and adjusted* mean difference / odds ratios (95% CI) for lifestyle characteristics for ethnic and generation groups

	White British N=3503	All Pakistani births N=3656	Pakistani sub-groups defined by place of birth of parents				
			Pakistani: Woman & partner UK born† N=383	Pakistani: Woman UK born, partner SA born† N=992	Pakistani: Partner UK born, woman SA born† N=876	Pakistani: Woman & partner SA born† N=1060	Pakistani: Other N=345
**BMI at start of pregnancy** Mean (sd)	**26.8 (5.9)**	**25.7 (5.4)**	24.3 (4.6)	26.7 (5.7)	24.4 (4.7)	26.4 (5.6)	25.4 (5.3)
Unadjusted mean difference	**0**	**-1.15 (-1.41, -0.88)**	-2.53 (-3.13, -1.94)	-0.15 (-0.55, 0.25)	-2.44 (-2.86, -2.02)	-0.43 (-0.82, -0.04)	-1.40 (-2.02, -0.77)
Adjusted mean difference: Model 1*	**0**	**-1.75 (-2.01, -1.49)**	-2.84 (-3.41, -2.26)	-0.73 (-1.12, -0.34)	-2.95 (-3.36, -2.54)	-1.49 (-1.88, -1.10)	-1.22 (-1.83, -0.62)
Adjusted mean difference: Model 2**	**0**	**-1.12 (-1.43, -0.81)**	-2.32 (-2.92, -1.72)	-0.35 (-0.76, 0.07)	-2.22 (-2.69, -1.75)	-0.99 (-1.43, -0.57)	-0.77 (-1.39, -0.15)
**Smoked during pregnancy** Number (%)	**1183 (34)**	123 (3)	25 (7)	47 (5)	7 (0.8)	18 (2)	26 (8)
Unadjusted OR	**1**	**0.07 (0.06, 0.08)**	0.14 (0.09, 0.21)	0.09 (0.07, 0.13)	0.02 (0.01, 0.03)	0.03 (0.02, 0.05)	0.16 (0.11, 0.24)
Adjusted OR: Model 1*	**1**	**0.06 (0.05, 0.07)**	0.13 (0.09, 0.20)	0.09 (0.06, 0.12)	0.01 (0.01, 0.03)	0.03 (0.02, 0.05)	0.12 (0.08, 0.19)
Adjusted OR: Model 2**	**1**	**0.06 (0.05, 0.08)**	0.15 (0.09, 0.23)	0.09 (0.07, 0.13)	0.01 (0.01, 0.03)	0.03 (0.02, 0.05)	0.13 (0.08, 0.19)

†All four parents of the woman & her partner South Asian (SA) born

*Adjusted for maternal age; parity
**Adjusted for maternal age; parity; employment status; level of education, receipt of means tested benefits; housing tenure

For peer review only - http://bmjopen.bmj.com/site/about/guidelines.xhtml

Table 4 Unadjusted and adjusted* mean difference / odds ratios (95% CI) for health related pregnancy characteristics for ethnic and generation groups

	White British N=3503	All Pakistani births N=3656	Pakistani sub-groups defined by place of birth of parents				
			Pakistani: Woman & partner UK born† N=383	Pakistani: Woman UK born, partner SA born† N=992	Pakistani: Partner UK born, woman SA born† N=876	Pakistani: Woman & partner SA born† N=1060	Pakistani: Other N=345
**Hypertensive disorders of pregnancy** Number (%)	**239 (7)**	**188 (5)**	16 (4)	51 (5)	43 (5)	66 (6)	12 (3)
Unadjusted OR	**1**	**0.74 (0.61, 0.90)**	0.59 (0.35, 0.99)	0.74 (0.54, 1.01)	0.70 (0.51, 0.98)	0.91 (0.68, 1.20)	0.49 (0.27, 0.89)
Adjusted OR: Model 1*	**1**	**0.82 (0.67, 1.01)**	0.62 (0.37, 1.04)	0.82 (0.59, 1.12)	0.85 (0.61, 1.19)	0.99 (0.74, 1.33)	0.56 (0.31, 1.01)
Adjusted OR: Model 2**	**1**	**0.82 (0.64, 1.04)**	0.62 (0.36, 1.06)	0.81 (0.58, 1.13)	0.87 (0.59, 1.29)	1.01 (0.73, 1.40)	0.56 (0.31, 1.03)
Adjusted OR: Model 3***	**1**	**0.87 (0.67, 1.13)**	0.78 (0.45, 1.35)	0.80 (0.56, 1.14)	1.06 (0.70, 1.61)	1.06 (0.75, 1.49)	0.57 (0.31, 1.06)
**Gestational diabetes** Number (%)	**172 (5)**	406 (11)	30 (8)	96 (10)	92 (11)	159 (15)	29 (8)
Unadjusted OR	**1**	2.42 (2.01, 2.91)	1.65 (1.09, 2.46)	2.07 (1.59, 2.69)	2.27 (1.74, 2.96)	3.42 (2.72, 4.29)	1.78 (1.18, 2.68)
Adjusted OR: Model 1*	**1**	2.41 (1.98, 2.94)	1.66 (1.10, 2.49)	2.07 (1.58, 2.71)	2.54 (1.92, 3.35)	3.01 (2.36, 3.83)	2.24 (1.47, 3.41)
Adjusted OR: Model 2**	**1**	2.28 (1.82, 2.86)	1.66 (1.09, 2.53)	1.98 (1.49, 2.64)	2.47 (1.79, 3.39)	2.89 (2.20, 3.82)	2.21 (1.44, 3.40)
Adjusted OR: Model 3***	**1**	2.38 (1.86, 3.03)	1.89 (1.23, 2.92)	1.98 (1.46, 2.67)	2.82 (2.01, 3.97)	3.04 (2.27, 4.08)	2.29 (1.47, 3.56)

For peer review only - http://bmjopen.bmj.com/site/about/guidelines.xhtml

**Fasting glucose** Mean (sd)	**4.41 (0.41)**	**4.62 (0.62)**	4.54 (0.47)	4.58 (0.64)	4.54 (0.48)	4.73 (0.76)	4.60 (0.53)
Unadjusted mean difference	**0**	**0.20 (0.18, 0.23)**	0.13 (0.08, 0.19)	0.17 (0.14, 0.21)	0.13 (0.09, 0.17)	0.32 (0.29, 0.36)	0.19 (0.13, 0.25)
Adjusted mean difference: Model 1*	**0**	**0.18 (0.16, 0.21)**	0.12 (0.06, 0.17)	0.15 (0.11, 0.19)	0.12 (0.09, 0.16)	0.27 (0.24, 0.31)	0.22 (0.16, 0.27)
Adjusted mean difference: Model 2**	**0**	**0.18 (0.15, 0.21)**	0.12 (0.06, 0.18)	0.15 (0.11, 0.19)	0.12 (0.07, 0.16)	0.27 (0.23, 0.31)	0.22 (0.16, 0.27)
Adjusted mean difference: Model 3***	**0**	**0.20 (0.17, 0.24)**	0.17 (0.12, 0.23)	0.16 (0.12, 0.19)	0.17 (0.12, 0.21)	0.29 (0.25, 0.33)	0.23 (0.17, 0.29)
**Postload glucose** Mean (sd)	**5.47 (1.30)**	**5.89 (1.68)**	5.59 (1.35)	5.81 (1.58)	5.82 (1.50)	6.12 (2.02)	5.73 (1.45)
Unadjusted mean difference	**0**	**0.42 (0.35, 0.49)**	0.12 (-0.04, 0.28)	0.34 (0.23, 0.45)	0.35 (0.24, 0.46)	0.72 (0.62, 0.83)	0.26 (0.09, 0.42)
Adjusted mean difference: Model 1*	**0**	**0.37 (0.29, 0.44)**	0.08 (-0.07, 0.24)	0.29 (0.18, 0.39)	0.35 (0.24, 0.46)	0.58 (0.48, 0.69)	0.35 (0.19, 0.51)
Adjusted mean difference: Model 2**	**0**	**0.35 (0.27, 0.43)**	0.10 (-0.06, 0.26)	0.28 (0.17, 0.39)	0.33 (0.20, 0.46)	0.56 (0.44, 0.68)	0.34 (0.18, 0.51)
Adjusted mean difference: Model 3***	**0**	**0.37 (0.28, 0.45)**	0.18 (0.02, 0.34)	0.27 (0.16, 0.38)	0.39 (0.26, 0.52)	0.57 (0.45, 0.69)	0.35 (0.18, 0.52)
**Fasting insulin** Mean (sd)	**81.40 (46.72)**	**100.28 (62.76)**	92.66 (65.59)	100.76 (56.46)	91.75 (49.04)	106.11 (68.84)	111.09 (81.89)
Unadjusted mean difference	**0**	**18.88 (16.31, 21.45)**	11.26 (5.42, 17.09)	19.36 (15.46, 23.26)	10.36 (6.26, 14.45)	24.71 (20.91, 28.51)	29.69 (23.58, 35.81)
Adjusted mean difference: Model 1*	**0**	**18.08 (15.42, 20.74)**	10.98 (5.13, 16.82)	18.59 (14.64, 22.54)	9.67 (5.51, 13.83)	23.36 (19.43, 27.30)	29.69 (23.55, 35.82)
Adjusted mean difference: Model 2**	**0**	**21.29 (18.13, 24.45)**	14.01 (7.95, 20.08)	20.62 (16.40, 24.83)	13.53 (8.73, 18.34)	25.24 (20.89, 29.59)	32.01 (25.72, 38.31)
Adjusted mean difference: Model 3***	**0**	**25.71 (22.73, 28.69)**	24.44 (19.03, 29.86)	21.29 (17.47, 25.13)	23.27 (18.86, 27.68)	29.03 (25.04, 33.02)	34.79 (29.18, 40.39)

†All four parents of the woman & her partner South Asian (SA) born
*Adjusted for maternal age; parity
**Adjusted for maternal age; parity; employment status; level of education, receipt of means tested benefits; housing tenure
*** Adjusted for maternal age; parity; employment status; level of education, receipt of means tested benefits; housing tenure; early pregnancy BMI; smoking in pregnancy

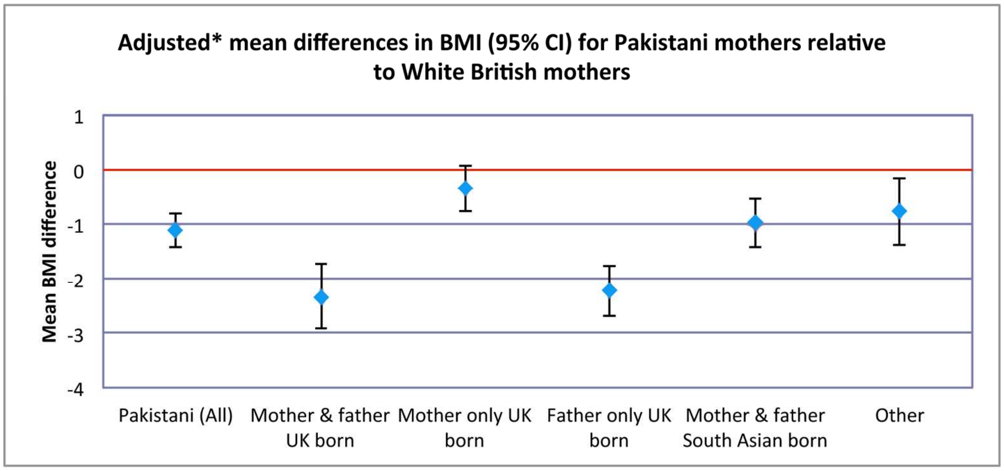

168x78mm (300 x 300 DPI)

For peer review only

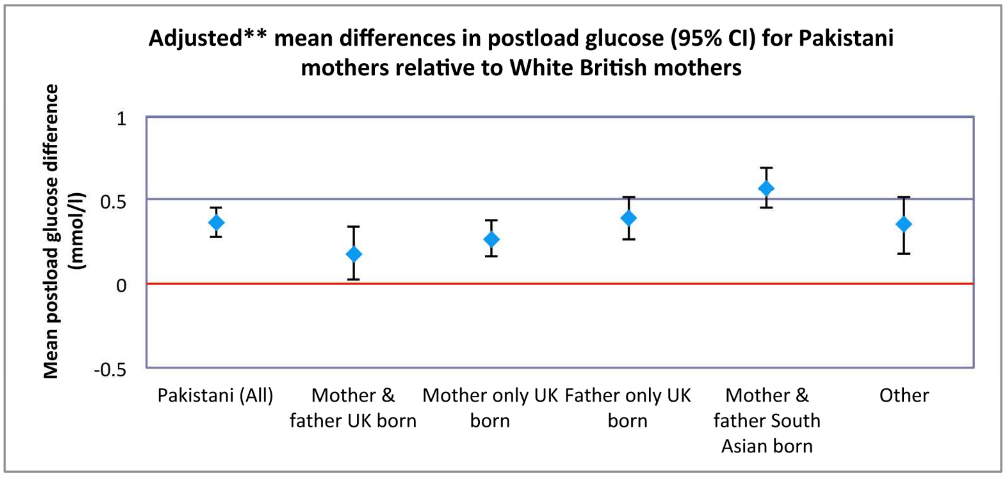

166x78mm (300 x 300 DPI)

For peer review only

STROBE Statement—Checklist of items that should be included in reports of ***cohort studies***

	Item No	Recommendation
**Title and abstract**	1	(*a*) The study's design is indicated in the title or the abstract (page 2)
		(*b*) Informative and balanced summary provided in abstract (page 2)
**Introduction**		
Background/rationale	2	Scientific background and rationale for the investigation being reported explained (page 4-6)
Objectives	3	Specific objectives stated (page 5)
**Methods**		
Study design	4	Key elements of study design presented (pages 6 & 7)
Setting	5	The setting, locations, and relevant dates, including periods of recruitment, exposure, follow-up, and data collection described (pages 6 & 7)
Participants	6	(*a*) Eligibility criteria and methods of follow-up given (page 6)
		(*b*) For matched studies, give matching criteria and number of exposed and unexposed N/A
Variables	7	All outcomes, exposures, predictors, potential confounders, and effect modifiers clearly defined (page 6 & 7)
Data sources/ measurement	8*	Sources of data and details of methods of assessment given. (pages 6 & 7)
Bias	9	Potential sources of bias discussed (page 12)
Study size	10	Study size described (page 6)
Quantitative variables	11	Means and sd /medians IQR were reported for continuous variables (pages 8 & 9)
Statistical methods	12	(*a*) All statistical methods, including those used to control for confounding described (page 7)
		(*b*) Describe any methods used to examine subgroups and interactions N/A
		(*c*) Explain how missing data were addressed : N/A
		(*d*) If applicable, explain how loss to follow-up was addressed N/A
		(*e*) Describe any sensitivity analyses N/A
**Results**		
Participants	13*	(a) Report numbers of individuals at each stage of study—eg numbers potentially eligible, examined for eligibility, confirmed eligible, included in the study, completing follow-up, and analysed (page 6)
		(b) Give reasons for non-participation at each stage N/A
		(c) Consider use of a flow diagram – described in methods
Descriptive data	14*	(a) Give characteristics of study participants (eg demographic, clinical, social) and information on exposures and potential confounders: included on page 6
		(b) Indicate number of participants with missing data for each variable of interest: N/A
		(c) Summarise follow-up time (eg, average and total amount) N/A (birth data)
Outcome data	15*	Report numbers of outcome events or summary measures over time: outcomes reported in results pages 8 & 9
Main results	16	(*a*) Give unadjusted estimates and, if applicable, confounder-adjusted estimates and their precision (eg, 95% confidence interval). Make clear which confounders were adjusted for and why they were included: included in main manuscript and tables
		(*b*) Report category boundaries when continuous variables were categorized: N/A
		(*c*) If relevant, consider translating estimates of relative risk into absolute risk for a

For peer review only - http://bmjopen.bmj.com/site/about/guidelines.xhtml

		meaningful time period N/A
Other analyses	17	Report other analyses done—eg analyses of subgroups and interactions, and sensitivity analyses N/A
**Discussion**		
Key results	18	Key results with reference to study objectives summarised (page 10)
Limitations	19	Limitations of the study, taking into account sources of potential bias or imprecision discussed. Limitations discussed (page 12)
Interpretation	20	Give a cautious overall interpretation of results considering objectives, limitations, multiplicity of analyses, results from similar studies, and other relevant evidence: included in discussion (pages10-12)
Generalisability	21	Discuss the generalisability (external validity) of the study results: included in discussion (page 12)
**Other information**		
Funding	22	Sources of funding and the role of the funders for the present study included (at end of manuscript)

For peer review only

For peer review only - http://bmjopen.bmj.com/site/about/guidelines.xhtml
